# Iron-Catalyzed Intermolecular N–H Insertion Using Acceptor–Acceptor Carbenes Derived from Iodonium Ylides

**DOI:** 10.1021/acs.orglett.5c02000

**Published:** 2025-07-15

**Authors:** Àlex Díaz-Jiménez, Nil Insa-Carreras, Anna Roglans, Anna Pla-Quintana, Miquel Costas

**Affiliations:** Institut de Química Computacional i Catàlisi (IQCC) and Departament de Química, 16738 Universitat de Girona, M. Aurèlia Capmany, 69, 17003 Girona, Catalonia, Spain

## Abstract

In this study, we report a general and efficient method for iron-catalyzed intermolecular N–H insertion at the intercarbonylic position of malonate reagents. Using iodonium ylides and simple iron­(II) triflate as the catalyst, the reaction enables the functionalization of a wide range of primary and secondary aromatic and aliphatic amines in excellent yields. The reaction operates under exceptionally mild conditions, without the need for an inert atmosphere or anhydrous solvents, and proceeds in remarkably short reaction times. Mechanistic studies support a nonradical pathway in which an iron­(II)-stabilized intermediate having hybrid carbene/carbocationic character undergoes nucleophilic attack by the amine.

## Graphical abstract

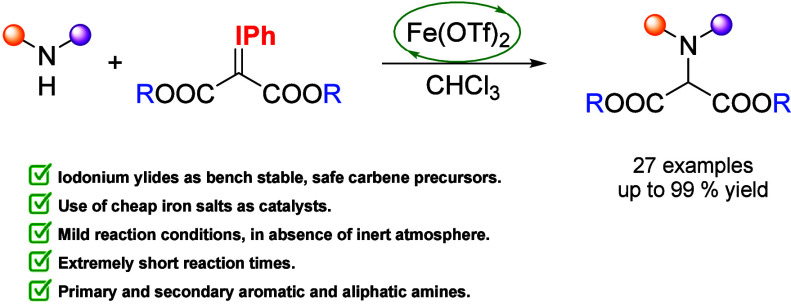

Metal carbenes are regarded among the most versatile intermediates in organic chemistry, given their ability to react with a wide range of functional groups, thus enabling the efficient and straightforward construction of diverse chemical bonds.^1,2^ Among these, N–H insertion has emerged as especially appealing given the ubiquity of nitrogen heterocycle-containing drugs and small-molecule drugs derived from amino acid derivatives.^3^

Traditionally, diazo compounds have been employed as precursors for generating metal carbenes, with particular emphasis on the generation of donor–acceptor metal carbenes.^4^ Instead, the use of acceptor–acceptor carbenes, both in general and particularly in N–H bond insertion reactions, has been largely overlooked in the literature. In the few reported cases, the successful insertion of acceptor–acceptor carbenes into N–H bonds typically requires the use of precious metals—Rh­(II) or Ir­(I)—catalysts (Scheme 1a). The groups of Livant^5a^ and Moody^5b^ reported N–H insertions of diazomalonates under Rh_2_(OAc)_4_ catalysis in refluxing toluene. These transformations are limited to the use of aniline derivatives or sterically hindered secondary alkylamines. Alternatively, the groups of Sivasankar and Lacour employed the [Ir­(COD)­Cl]_2_ catalyst at room temperature. The substrate scope described by Sivasankar et al.^6^ was limited to aromatic amines, while that of Lacour et al.^7^ extended to a broader array of aromatic and aliphatic amines.

**Scheme 1:**
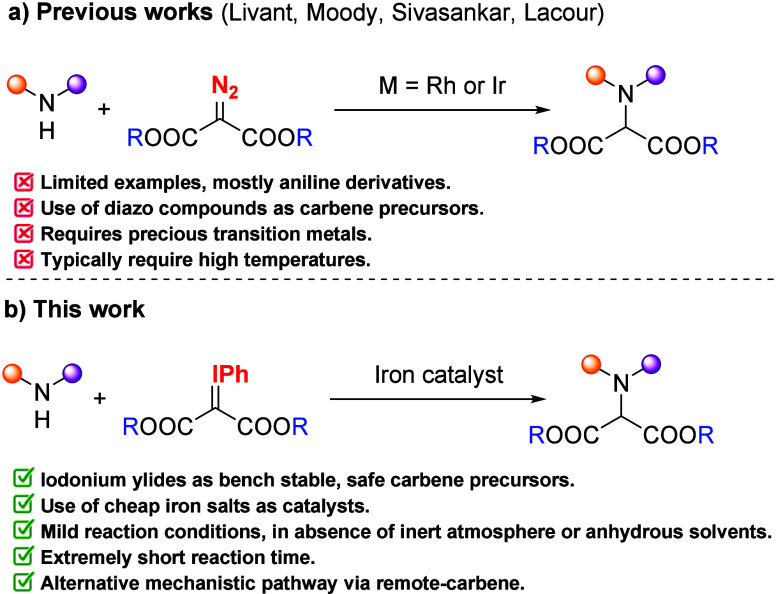
N–H Insertion Reactions with Acceptor–Acceptor Carbenes

With these precedents in hand, we envisioned that a methodology that avoids the use of diazo compounds as carbene precursors and precious metals as catalysts would represent a significant advance in the field of acceptor–acceptor carbene N–H insertion. First, iodonium ylides have emerged as promising alternatives to diazo compounds for the generation of acceptor–acceptor carbenes under mild conditions.^8^ These reagents are regarded as easily preparable, nontoxic, and bench stable solids that bypass the safety issues associated with diazo derivatives.^9^ Second, iron constitutes an excellent alternative to precious metals, due to its low toxicity, high natural abundance, and affordability.^10^ Nonetheless, iron-catalyzed carbene transfer reactions are underexplored and reports remain scarce.^11^ In the particular case of iron-catalyzed carbene N–H insertion reactions, the few reported cases using acceptor carbenes rely on the use of porphyrin-derived complexes or highly engineered metalloenzymes.^12^

Herein, we report the N–H insertion of acceptor–acceptor carbenes using iodonium ylides as carbene precursors and simple, commercially available iron catalysts. This highly efficient and straightforward methodology enables the insertion of carbenes into both primary and secondary aromatic and aliphatic amines in high yields and under exceptionally mild conditions, without the need for an inert atmosphere or anhydrous solvents and in short reaction times.

We began our studies by testing the viability of our hypothesis using iodonium ylide **1a** and 2 equiv of *p*-toluidine **2a** in the presence of iron­(II) acetylacetonate in anhydrous dichloromethane under inert conditions inside a glovebox for 15 min (entry 1, Table 1). This initial experiment yielded desired product **3a** in 54% yield. Encouraged by this result, we repeated the reaction outside the glovebox without employing anhydrous solvents (entry 2, Table 1). Remarkably, **3a** was obtained after 45 min in an improved yield of 74%, highlighting the high tolerance and robustness of our transformation. We next evaluated the use of various commercially available iron­(II) salts as catalysts. The use of iron­(II) acetate resulted in a significantly diminished yield of 17% after 14 h (entry 3, Table 1). In contrast, iron­(II) bromide and chloride afforded **3a** in 77% and 64% yields, respectively (entries 4–5, Table 1). Strikingly, when iron­(II) triflate was used, immediate solubilization of the iodonium ylide **1a** was observed, with the reaction finished within the mixing time, delivering **3a** in 88% yield (entry 6, Table 1). Further optimization revealed that the reaction could be carried out with equimolar amounts of the amine partner, affording **3a** in 92% yield (entry 7, Table 1). Switching the solvent to chloroform further improved the yield to 99% (entry 8, Table 1).^13^ We explored the impact of reduction of the catalyst loading to 2.5 mol %, leading to a slightly decreased yield of 84% (entry 9, Table 1). Control experiments were conducted to clarify the observed unique reactivity. The use of Fe­(OTf)_3_ or FeCl_3_ under the optimized conditions afforded **3a** in 54% and 30% yield, respectively. A blank experiment performed in the absence of any iron salt provided **3a** in only 12% yield after 14 h. Replacement of **1a** with dimethyl 2-diazomalonate led to recovery of starting materials, even upon extending the reaction time to 15 min. Collectively, these results underscore the catalytic role of iron, the superior performance of iron­(II) salts, and the essential role of the iodonium ylide as a carbene surrogate in this transformation. Finally, addition of 10 equiv of H_2_O under the optimized conditions afforded **3a** in 96% yield, with no detectable water-insertion byproducts, confirming the reaction’s tolerance to moisture.

**Table 1:**
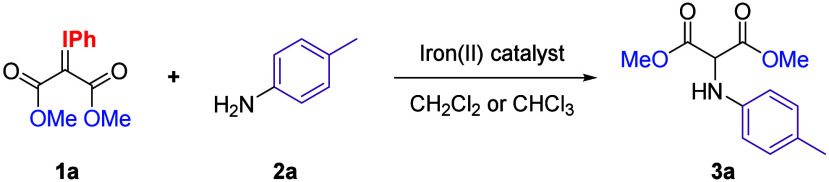
Optimization of the Reaction Conditions^a^

Entry	Catalyst	Reaction time	Yield % (**3a**)
1^b^	Fe(acac)_2_	15 min	54
2	Fe(acac)_2_	45 min	74
3	Fe(OAc)_2_	14 h	17
4	FeBr_2_	45 min	77
5	FeCl_2_	75 min	64
6	Fe(OTf)_2_	Mixing time	88
7^c^	Fe(OTf)_2_	Mixing time	92
8^c^ ^,^ d	Fe(OTf)_2_	Mixing time	99 (99)^e^
9^c^ ^,^ ^f^	Fe(OTf)_2_	Mixing time	84^e^

a Reactions were carried out with 0.08 mmol of iodonium ylide **1a**, 0.16 mmol of *p*-toluidine **2a**, and 5 mol % catalyst loading, at room temperature in 1 mL of CH_2_Cl_2_ for the time stated (until complete solubilization of the iodonium ylide). NMR yields calculated via ^1^H NMR spectroscopy using CH_2_Br_2_ as internal standard.

b Reaction carried out inside of the glovebox using anhydrous solvent.

c Reaction carried out with 0.08 equiv of *p*-toluidine **2a**.

d Reaction carried out in CHCl_3_ as the solvent.

e Isolated yield.

f Reaction carried out with 2.5 mol % catalyst loading.

With the optimized conditions in hand, we next explored the scope of the reaction (Scheme 2). We began by evaluating the effect of substitution on the aniline moiety. Both *meta*- and *ortho*-toluidine afforded the corresponding insertion products **3b** and **3c** in excellent yields of 90% and 92%, respectively. Notably, the sterically hindered 1,3,5-trimethylaniline also reacted smoothly, delivering **3d** in 89% yield. Unsubstituted aniline could also react efficiently, affording **3e** in an excellent 96% yield. After assessing the tolerance to steric bulk, we next investigated the electronic factors on the aniline ring. *Para*-substituted anilines bearing electron-donating methoxy or electron-withdrawing chlorine groups afforded **3f** and **3g** in quantitative yields. The strongly electron-withdrawing (and highly coordinating) nitrile-substituted aniline afforded **3h** in a slightly reduced 82% NMR yield. However, purification was unsuccessful due to coelution with unreacted *p*-cyanoaniline. Alternatively, we employed a *tert*-butyl-substituted iodonium ylide, yielding **3h′** in a moderate but easily isolable 56% yield. Unfortunately, the reaction with *p*-aminophenol and 2-aminoethanethiol did not proceed and led to only decomposition products. Besides monosubstituted anilines, the reaction was also compatible with aromatic secondary amines bearing methyl and phenyl groups, furnishing **3i** and **3j** in good yields. We then turned our attention to aliphatic amines. Benzylamine reacted cleanly to produce **3k** in a 94% yield. Notably, allylamine and propargylamine afforded exclusively the desired insertion products **3l** and **3m**, without any trace of cyclopropanation or cyclopropenation byproducts. Furthermore, the reaction with diallylamine proceeded efficiently, affording the N–H insertion product **3l′** in 99% yield. Interestingly, reaction of geranylamine—a more sterically and electronically demanding substrate bearing both internal and terminal trisubstituted alkenes—also showed excellent selectivity, furnishing the N–H insertion product **3l″** in 97% yield. In contrast, reactions with bulkier aliphatic amines such as cyclohexylamine and diethylamine afforded modest 18% yields of **3n** and **3o** along with the carbene dimerization product (**4**) formed as the major product. We hypothesized that the increased steric bulk of the amines slowed N–H insertion, thereby allowing the competing iodonium ylide dimerization to dominate. To suppress dimerization, a more sterically hindered *tert*-butyl-derived iodonium was employed. Gratifyingly, the reaction proceeded with significantly improved yields of **3n′** (87%) and **3o′** (77%). Following the same strategy, the reaction with an acetal-derived amine provided **3p′** in an excellent 97% yield.

**Scheme 2:**
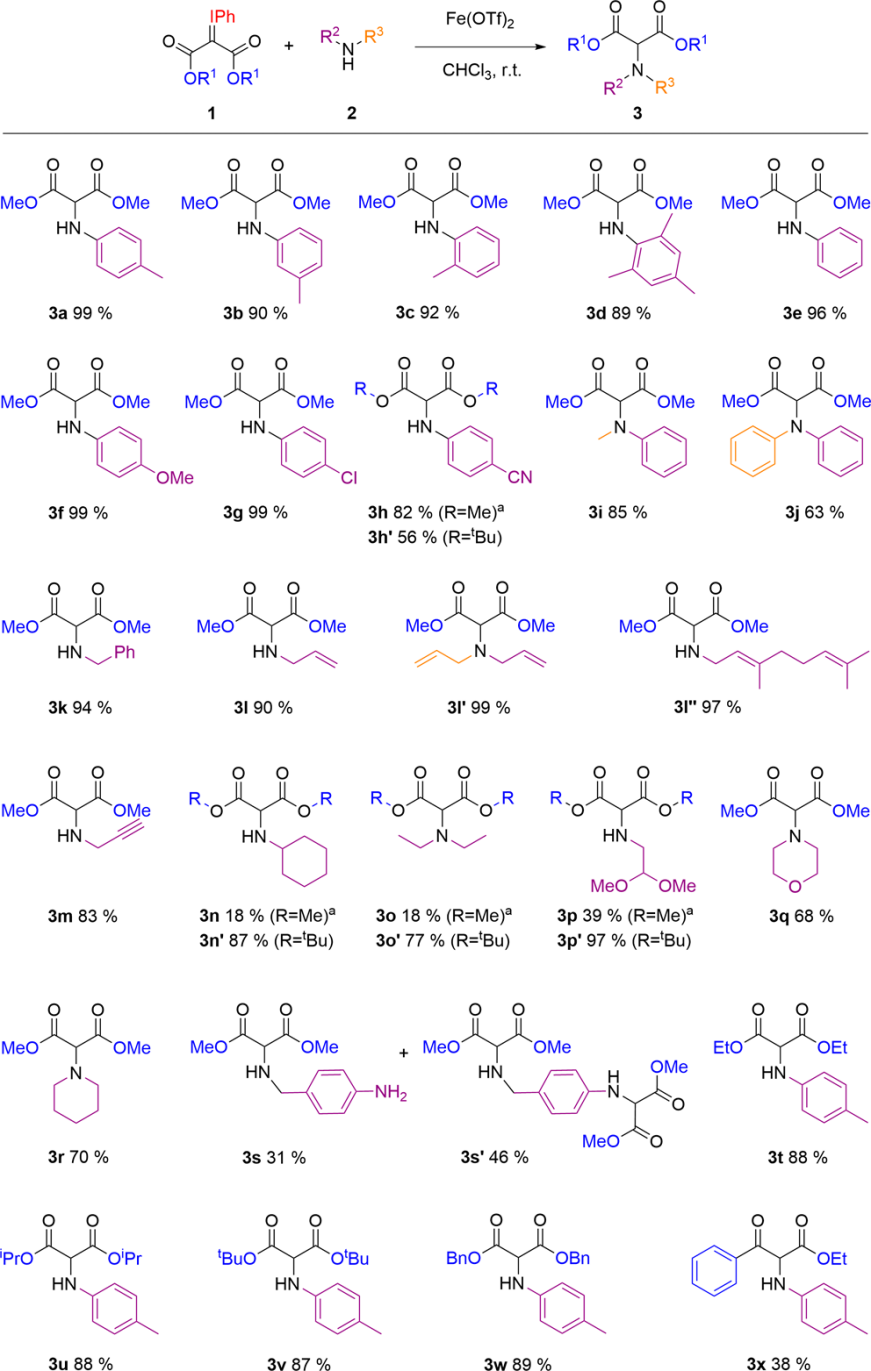
Scope of the Reaction

General conditions: Iodonium ylide **1** (0.16 mmol), amine derivative **2** (0.16 mmol, 1.0 equiv), and Fe­(OTf)_2_ (5 mol %) in CHCl_3_ (2.0 mL) at room temperature. The reactions were complete after the mixing time. Isolated yields.
a NMR yield.

In contrast, when N-Boc-protected propargylamine was tested with both methyl and *tert*-butyl iodonium ylides, no N–H insertion was observed in either case, and the reaction resulted predominantly in dimerization of the iodonium ylide. Cyclic secondary amines, such as morpholine and piperidine, were compatible with the reaction, affording **3q** and **3r** in good yields. To further evaluate the chemoselectivity of the system, we tested *p*-(*N*-benzylamino)­aniline. The reaction yielded a mixture of monoinserted **3s** and doubly functionalized **3s′,** in 31% and 46% yields, respectively, indicating a preference for the reaction to occur at the benzylic amine over the aromatic one. Finally, we examined the scope of the iodonium ylide partner. Substituents such as ethyl (**3t**), isopropyl (**3u**), *tert*-butyl (**3v**), and benzyl (**3w**) were all well tolerated, affording the corresponding insertion products in high yields. To evaluate the generality of the method beyond diester-containing substrates, we tested the iodonium ylide derived from methyl 3-oxo-3phenylpropanoate. The reaction proceeded smoothly, affording the desired product **3x** in 38% yield. Scaling up the reaction for the synthesis of **3a** to 1 mmol, mixing the solid reagents prior to solvent addition, lead to a spontaneous exothermic event;^14^ thus, the reagents were dissolved in chloroform before adding the iron catalyst, providing the product in a 71% yield. During the preparation of this manuscript, the group of Song^15^ reported the iron-catalyzed insertion of iodonium ylides into B–H and N–H bonds. Unlike in the current report, the reaction relies on a sophisticated iron catalyst, requires strictly inert and anhydrous conditions and significantly longer reaction times, and provided lower yields compared to our system, clearly highlighting the advantages of our approach.

To gain a deeper understanding, several mechanistic experiments were conducted (Scheme 3). First, we subjected **1a** to the optimized reaction conditions in the absence of any amine partner, leading to dimerized product **4** in a 64% NMR yield (Scheme 3A). Previous studies by Betley^16^ and Groysman^17^ have proposed the involvement of vinyl radical intermediates in reactions involving 1,3-dicarbonyl-derived carbene precursors and iron catalysts. DFT calculations by Groysman et al.^17^ suggest that the reaction of iodonium ylides with iron­(II) alkoxide proceeds via κ^2^ coordination through both carbonyl esters and concurrent oxidation, which activates the C–I bond, resulting in the formation of an iron­(III) remote carbene/vinyl radical intermediate **A** (Scheme 4). This intermediate reacts with alkenes in a stepwise manner to undergo cyclopropanation. On the other hand, the mechanism that Betley et al.^16^ described for their intramolecular C–H alkoxylation of α-diazo-β-ketoesters using Fe­(acac)_2_ as catalyst also involves a radical intermediate. Again, via κ^2^ coordination through the keto and ester carbonyls, a single electron transfer from iron to the substrate generates vinyl carbon radical intermediate **B** (Scheme 4), which further evolves via hydrogen atom transfer (HAT). However, several experiments argue against the implication of such a carbon-centered radical intermediate in our system. First, the reaction is not perturbed by the presence of O_2_. Moreover, when the reaction was carried out in the presence of 3 equiv of radical trapping agents such as TEMPO (Scheme 3B), the insertion product **3a** was obtained in 87% yield. Furthermore, the addition of BHT or 1,4-cyclohexadiene, well-established hydrogen atom donors, had no apparent effect on the reaction outcome (Scheme 3C), suggesting the absence of a competitive hydrogen atom transfer process typically associated with radical species. Altogether, these results point to a mechanistic pathway in which intermediates with a radical character are not implicated.

**Scheme 3:**
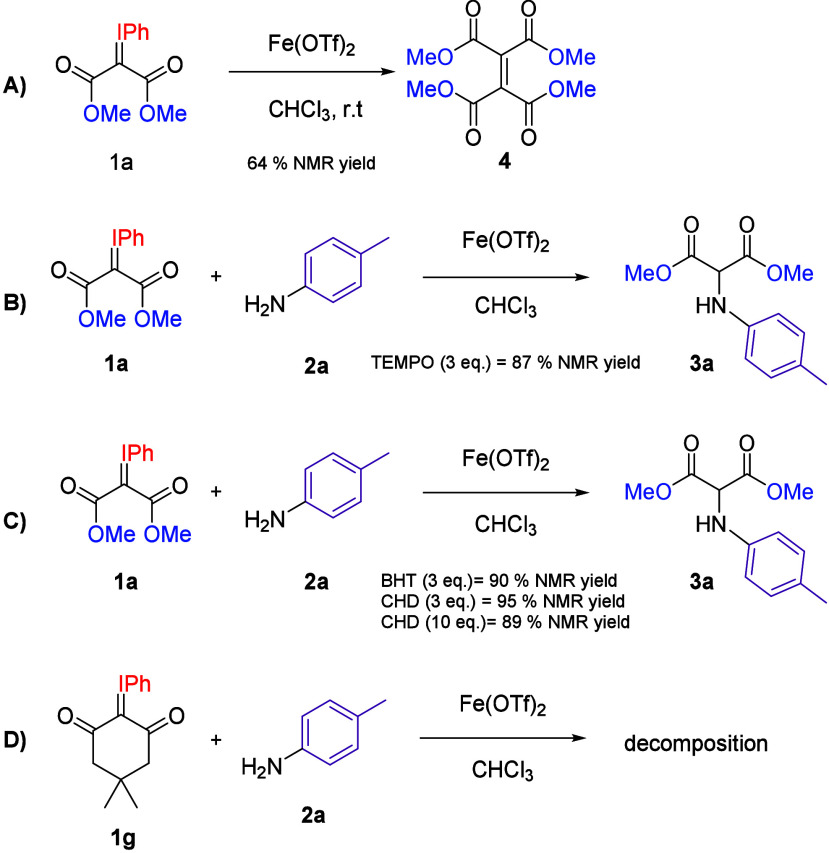
Mechanistic Experiments

**Scheme 4:**
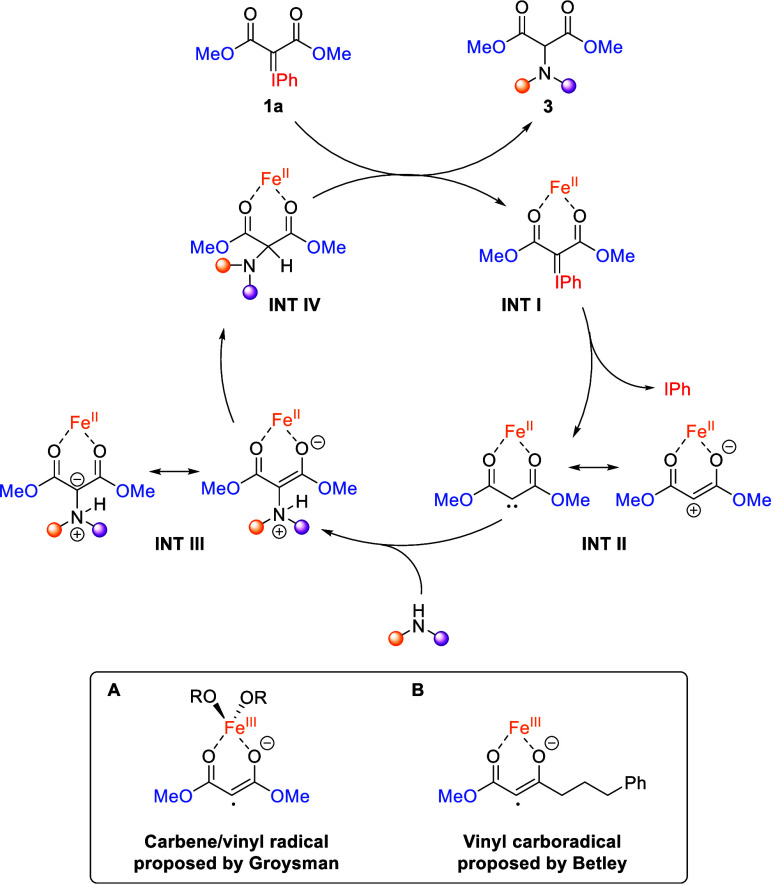
Proposed Mechanism

The fast reaction rates of the current catalytic system, combined with the low solubility of the iodonium ylides, prevented the identification of reaction intermediates that could support the characterization of the actual catalytic species or provide meaningful guidance to enable computational studies. Under these conditions, we propose a tentative alternative pathway—compatible with the available mechanistic information—in which the catalyst remains in the iron­(II) oxidation state (Scheme 4). Consistent with the precedents reported by Groysman and Betley, the iron center is κ^2^-coordinated to the two ester carbonyl groups. Supporting this coordination-based mechanism, the reaction with dimedone-derived iodonium ylide **1g** (Scheme 3D) failed and led exclusively to decomposition, likely due to the rigid cyclic structure preventing proper coordination to the iron center. This coordination, instead of promoting oxidation, stabilizes an intermediate that can be viewed as a hybrid between a vinyl carbocation and a “remote” carbene (**INT II**). Differences in the ligand set, which in the current case is less donating that in Groysman and Betley’s catalysts, may upshift the Fe­(III)/Fe­(II) red-ox potential, favoring the ferrous state. To the best of our knowledge, this would represent the first example of a remote carbene intermediate operating in a metal-carbene-type transformation. The carbocationic character of this intermediate accounts for its high reactivity toward nucleophilic amines, as well as the chemoselectivity observed with substrates bearing alkenes (**3l**) and alkynes (**3m**). This hypothesis was further supported by a control experiment using styrene under otherwise analogous conditions, which showed no evidence of cyclopropanation product formation, but only dimerization of the iodonium ylide.

To sum up, the proposed reaction mechanism (Scheme 4) begins with the initial coordination of the iron catalyst to iodonium ylide **1a**, forming intermediate **INT I**. This intermediate undergoes extrusion of iodobenzene to generate the iron remote carbene (**INT II**). Nucleophilic attack by the amine at the carbenic/carbocationic carbon yields ylide intermediate **INT III**, which undergoes a proton transfer followed by dissociation of final product **3a** from the iron center.

In conclusion, we have developed a novel method for the N–H insertion of acceptor–acceptor carbenes, utilizing iodonium ylides as carbene precursors and readily available, inexpensive iron­(II) triflate as the catalyst. The reaction proceeds efficiently under mild conditions, without the need for an inert atmosphere or anhydrous solvents, in remarkably short reaction times. Mechanistic studies support a nonradical pathway in which an iron­(II)-stabilized intermediate having hybrid carbene/carbocationic character undergoes nucleophilic attack by the amine.

## Supplementary Material



## Data Availability

The data underlying this study are available in the published article and its Supporting Information.

## Notes

Experimental details and spectral data for all unknown compounds (PDF)
