# Supplementary material for: Iron-Catalyzed Intermolecular N–H Insertion Using Acceptor–Acceptor Carbenes Derived from Iodonium Ylides
Source: Org Lett. 2025 Jul 15;27(29):7804–9. doi: 10.1021/acs.orglett.5c02000 (PMC12305656; doi:10.1021/acs.orglett.5c02000)

Supplementary Information

Iron-Catalyzed Intermolecular N-H Insertion Using Acceptor-Acceptor Carbenes Derived from Iodonium Ylides

Àlex Díaz-Jiménez, Nil Insa-Carreras, Anna Roglans, Anna Pla-Quintana,*

and Miquel Costas*

Institut de Quimica Computacional i Catàlisi (IQCC) and Departament de Química, Universitat de Girona, M. Aurèlia Capmany, 69, 17003 Girona, Catalonia, Spain. E-mail: miquel.costas@udg.edu

TABLE OF CONTENTS

General materials and methods 3

S1. Starting materials 4

S2. General procedure for the iron catalyzed intermolecular N-H insertion 5

S3. Mechanistic studies 17

a) Reaction in absence of the amine 17

S4. References 17

S5. NMR SPECTRA 19

General materials and methods

Unless otherwise noted, materials were obtained from commercial suppliers and used without further purification. Reaction progress during the preparation of all compounds was monitored using thin layer chromatography on Macherey-Nagel Xtra SIL G/UV254 silica gel plates. Solvents were removed under reduced pressure with a rotary evaporator. Reaction mixtures were chromatographed on silica gel using an automated purification instrument Interchim PuriFlash XS 520 Plus equipped with a quaternary gradient pump (up to 300 ml/min, 20 bar) and an UV-Vis 200-800 nm diode array detector. All ^{1}H and ^{13}C NMR spectra were recorded on a Bruker ASCEND 400 spectrometer equipped with a 5 mm BBFO probe using $CDCl_3$ a deuterated solvent. Chemical shifts for ^{1}H and ^{13}C NMR are reported in ppm (δ) relative to residual solvent signals ($CDCl_3$: 7.26 ppm for ^{1}H, 77,16 ppm for ^{13}C). Coupling constants are given in Hertz (Hz). Electrospray ionization high-resolution mass spectrometry was performed using a Bruker microTOF-Q II instrument operated in the positive ESI(+) ion mode. IR spectra were recorded on an Agilent Cary 630 FT-IR spectrometer equipped with an ATR sampling accessory. Melting points were measured in a SMP10 apparatus from Stuart without any correction.

S1. Starting materials

All iodonium ylides were prepared following the procedure reported by de Bruin *et al.*[1] The spectroscopic data of iodonium ylides **1** matches the one previously reported in the literature.[1,2]

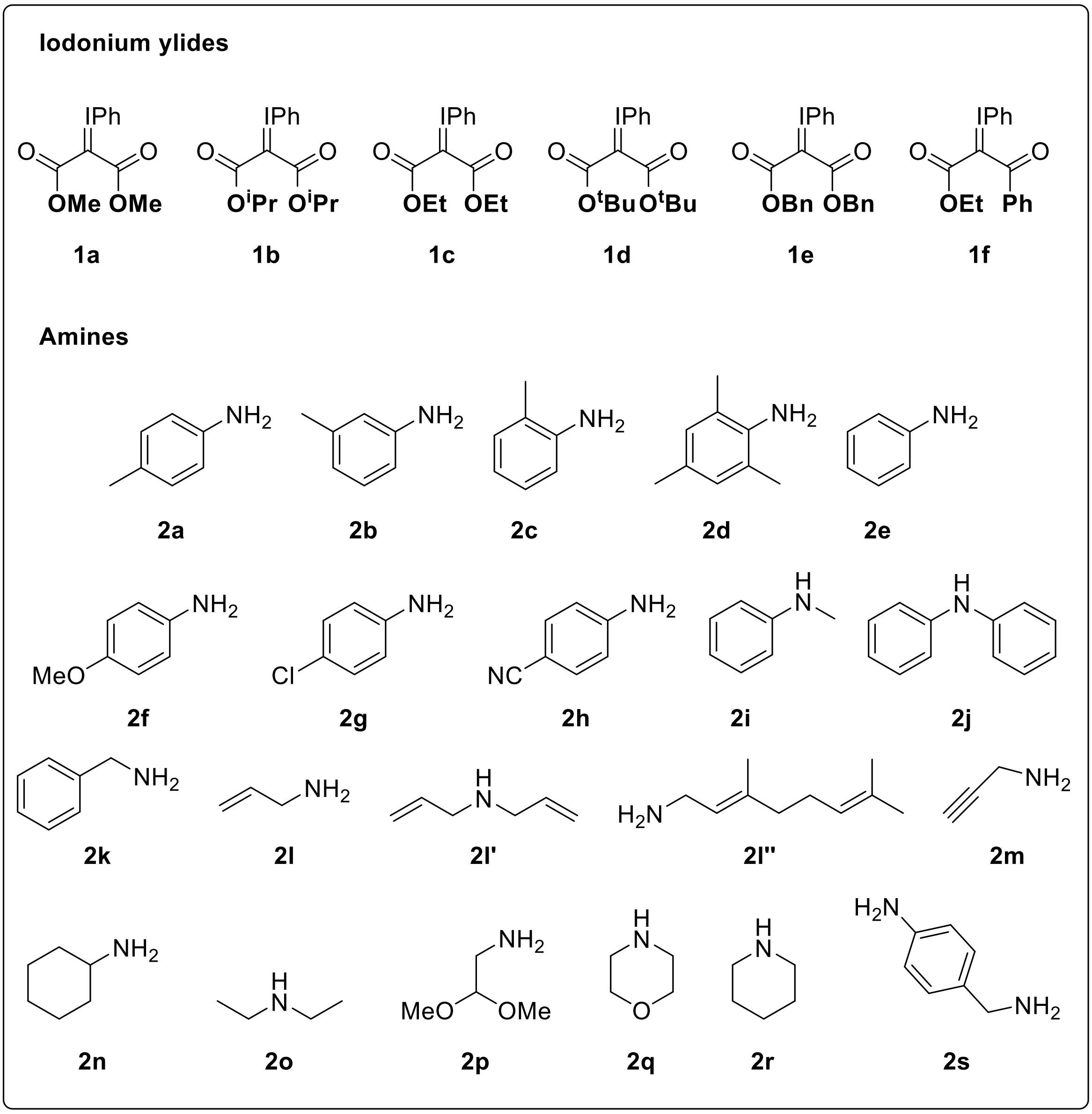

S2. General procedure for the iron catalyzed intermolecular N-H insertion

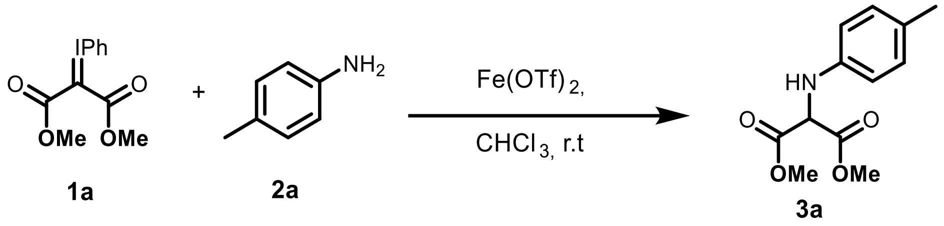

A mixture of iodonium ylide **1a** (53.4 mg, 0.16 mmol, 1 equiv.), $Fe(OTf)_2$ (2.8 mg, 0.008 mmol, 5 mol%) and *p*-toluidine **2a** (17.1 mg, 0.16 mmol, 1 equiv.) was placed into a 10-mL vial, and chloroform (2 mL, [M] = 0.08 M) was then added. Upon addition of the chloroform, immediate solubilization of all solids indicated the termination of the reaction. The crude mixture was then filtered through a silica plug to remove impurities and the solvent was evaporated under reduced pressure. The crude reaction mixture was then purified by column chromatography on silica gel using hexane/EtOAc mixtures (94:6 to 86:14) as the eluent to afford compound **3a** (37.6 mg, 99% yield) as a colourless solid.

Large scale reaction:

A mixture of iodonium ylide **1a** (335.3 mg, 1 mmol, 1 equiv.) and *p*-toluidine **2a** (107.2 mg, 1 mmol, 1 equiv.) was placed into a 10-mL vial, and chloroform (5 mL, [M] = 0.2 M) was added. $Fe(OTf)_2$ (17.7 mg, 0.05 mmol, 5 mol%) was then added to the reaction mixture. Upon addition of the iron catalyst, immediate solubilization of all solids indicated the termination of the reaction. The crude mixture was then filtered through a silica plug to remove impurities and the solvent was evaporated under reduced pressure. The crude reaction mixture was then purified by column chromatography on silica gel using hexane/EtOAc mixtures (94:6 to 86:14) as the eluent to afford compound **3a** (169.3 mg, 71% yield) as a colourless solid.

MW ($C_{12}H_{15}NO_4$): 237.10 g/mol; **Rf:** 0.57 (Hexanes/EtOAc, 8:2); **IR (ATR) ν (cm^{-1}):** 3360, 1736, 1155; **MP (ºC):** 73 – 76; **^{1}H NMR ($CDCl_3$, 400 MHz):** δ_H 7.01 (d, *J* = 8.3 Hz, 2H), 6.58 (d, *J* = 8.3 Hz, 2H), 4.78 (d, *J* = 7.9 Hz, 1H), 4.69 (d, *J* = 7.9 Hz, 1H), 3.81 (s, 6H), 2.24 (s, 3H); **^{13}C{H} NMR ($CDCl_3$, 101 MHz):** δ_C 168.4, 143.1, 130.1, 128.6, 113.8, 61.0, 53.4, 20.6; **HRMS (ESI) m/z:** $[M+H]^+$ calcd. for $C_{12}H_{16}NO_4$: 238.1074; Found: 238.1075.

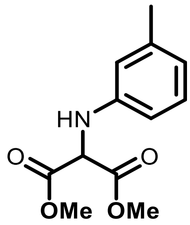

3b

Starting from iodonium ylide **1a** (53.3 mg, 0.16 mmol) and aniline **2b** (17.1 µL, 0.16 mmol) in $CHCl_3$ (2 mL, [M] = 0.08 mol/l) using $Fe(OTf)_2$ (2.8 mg, 0.08 mmol, 5 mol%), the crude reaction mixture was purified by column chromatography on silica gel using hexane/EtOAc mixtures (94:6 to 90:10) as the eluent to afford compound **3b** as a colourless solid (33.9 mg, 90% yield).

MW ($C_{12}H_{15}NO_4$): 237.10 g/mol; **Rf:** 0.53 (Hexanes/EtOAc, 8:2); **IR (ATR) *ν* (cm^{-1}):** 3394, 1730, 1216; **MP (ºC):** 65 – 68; **^{1}H NMR ($CDCl_3$, 400 MHz):** δ_H 7.08 (t, *J* = 7.6 Hz, 1H), 6.62 (d, *J* = 7.6 Hz, 1H), 6.52 – 6.42 (m, 2H), 4.79 (bs, 2H), 3.82 (s, 6H), 2.28 (s, 3H); **^{13}C{H} NMR ($CDCl_3$, 101 MHz):** δ_C 168.4, 145.4, 139.5, 129.5, 120.3, 114.6, 110.6, 60.7, 53.4, 21.7; **HRMS (ESI) m/z:** $[M+H]^+$ calcd. for $C_{12}H_{16}NO_4$: 238.1074; Found: 238.1079.

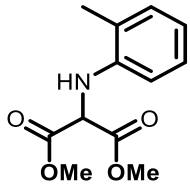

3c

Starting from iodonium ylide **1a** (53.2 mg, 0.16 mmol) and aniline **2c** (17.0 µL, 0.16 mmol) in $CHCl_3$ (2 mL, [M] = 0.08 mol/l) using $Fe(OTf)_2$ (2.8 mg, 0.08 mmol, 5 mol%), the crude reaction mixture was purified by column chromatography on silica gel using hexane/EtOAc mixtures (94:6 to 90:10) as the eluent to afford compound **3c** as a yellow oil (34.8 mg, 92% yield).

MW ($C_{12}H_{15}NO_4$): 237.10 g/mol; **Rf:** 0.50 (Hexanes/EtOAc, 8:2); **IR (ATR) *ν* (cm^{-1}):** 3404, 1729, 1224; **^{1}H NMR ($CDCl_3$, 400 MHz):** δ_H 7.14 – 7.06 (m, 2H), 6.74 (t, *J* = 7.6 Hz, 1H), 6.52 (d, *J* = 7.6 Hz, 1H), 4.82 (s, 1H), 4.76 (bs, 1H), 3.83 (s, 6H), 2.26 (s, 3H); **^{13}C{H} NMR ($CDCl_3$, 101 MHz):** δ_C 168.5, 143.6, 130.7, 127.3, 123.2, 118.9, 110.6, 60.8, 53.5, 17.5; **HRMS (ESI) m/z:** $[M+H]^+$ calcd. for $C_{12}H_{16}NO_4$: 238.1074; Found: 238.1078.

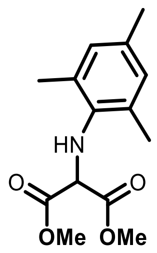

3d

Starting from iodonium ylide **1a** (53.4 mg, 0.16 mmol) and aniline **2d** (22.4 µL, 0.16 mmol) in $CHCl_3$ (2 mL, [M] = 0.08 mol/l) using $Fe(OTf)_2$ (2.8 mg, 0.08 mmol, 5 mol%), the crude reaction mixture was purified by column chromatography on silica gel using hexane/EtOAc mixtures (94:6 to 90:10) as the eluent to afford compound **3d** as a yellow solid (37.6 mg, 89% yield).

MW ($C_{14}H_{19}NO_4$): 265.13 g/mol; **Rf:** 0.70 (Hexanes/EtOAc, 8:2); **IR (ATR) *ν* (cm^{-1}):** 3386, 1737, 1219; **MP (ºC):** 72 – 75; **^{1}H NMR ($CDCl_3$, 400 MHz):** δ_H 6.79 (s, 2H), 4.65 (d, *J* = 9.1 Hz, 1H), 4.30 (d, *J* = 9.1 Hz, 1H), 3.75 (s, 6H), 2.29 (s, 6H), 2.20 (s, 3H); **^{13}C{H} NMR ($CDCl_3$, 101 MHz):** δ_C 169.0, 140.2, 132.0, 129.8, 129.3, 63.6, 53.1, 20.7, 18.6; **HRMS (ESI) m/z:** $[M+H]^+$ calcd. for $C_{14}H_{20}NO_4$: 266.1387; Found: 266.1382.

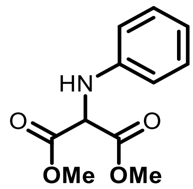

3e

Starting from iodonium ylide **1a** (53.3 mg, 0.16 mmol) and aniline **2e** (14.6 µL, 0.16 mmol) in $CHCl_3$ (2 mL, [M] = 0.08 mol/l) using $Fe(OTf)_2$ (2.8 mg, 0.08 mmol, 5 mol%), the crude reaction mixture was purified by column chromatography on silica gel using hexane/EtOAc mixtures (94:6 to 90:10) as the eluent to afford compound **3e** was obtained as a yellow oil (34.2 mg, 96% yield).

MW ($C_{11}H_{13}NO_4$): 223.08 g/mol; **Rf:** 0.25 (Hexanes/EtOAc, 8:2); **^{1}H NMR ($CDCl_3$, 400 MHz):** δ_H 7.24 – 7.13 (m, 2H), 6.80 (t, *J* = 7.6 Hz, 1H), 6.66 (d, *J* = 7.6 Hz, 2H), 4.80 (bs, 2H), 3.82 (s, 6H); **^{13}C{H} NMR ($CDCl_3$, 101 MHz):** δ_C 168.3, 145.4, 129.6, 119.3, 113.6, 60.6, 53.5.

The spectroscopic data of **3e** agrees with those previously reported in the literature.[4]

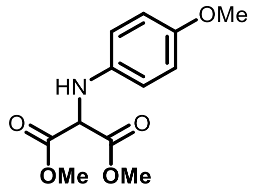

3f

Starting from iodonium ylide **1a** (53.9 mg, 0.16 mmol) and aniline **2f** (19.7 mg, 0.16 mmol) in $CHCl_3$ (2 mL, [M] = 0.08 mol/l) using $Fe(OTf)_2$ (2.8 mg, 0.08 mmol, 5 mol%), the crude reaction mixture was purified by column chromatography on silica gel using hexane/EtOAc mixtures (94:6 to 86:14) as the eluent to afford compound **3f** as a colourless solid (40.5 mg, 99% yield).

MW ($C_{12}H_{15}NO_5$): 253.10 g/mol; **Rf:** 0.40 (Hexanes/EtOAc, 8:2); **^{1}H NMR ($CDCl_3$, 400 MHz):** δ_H 6.83 – 6.74 (m, 2H), 6.68 – 6.59 (m, 2H), 4.73 (d, J = 6.8 Hz, 1H), 4.55 (d, J = 6.8 Hz, 1H), 3.81 (s, 6H), 3.74 (s, 3H); **^{13}C{H} NMR ($CDCl_3$, 101 MHz):** δ_C 168.5, 153.4, 139.5, 115.3, 115.1, 61.7, 55.8, 53.4.

The spectroscopic data of **3f** agrees with those previously reported in the literature.[4]

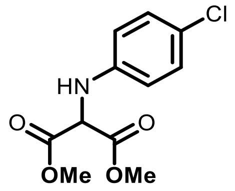

3g

Starting from iodonium ylide **1a** (53.0 mg, 0.16 mmol) and aniline **2g** (20.4 mg, 0.16 mmol) in $CHCl_3$ (2 mL, [M] = 0.08 mol/l) using $Fe(OTf)_2$ (2.8 mg, 0.08 mmol, 5 mol%), the crude reaction mixture was purified by column chromatography on silica gel using hexane/EtOAc mixtures (94:6 to 90:10) as the eluent to afford compound **3g** as a colourless solid (40.5 mg, 99% yield).

MW ($C_{11}H_{12}ClNO_4$): 257.05 g/mol; **Rf:** 0.60 (Hexanes/EtOAc, 8:2); **IR (ATR) ν (cm^{-1}):** 3398, 1733, 1209; **MP (ºC):** 84 – 86; **^{1}H NMR ($CDCl_3$, 400 MHz):** δ_H 7.19 – 7.10 (m, 2H), 6.62 – 6.54 (m, 2H), 4.83 (d, J = 7.8 Hz, 1H), 4.74 (d, J = 7.8 Hz, 1H), 3.82 (s, 6H); **^{13}C{H} NMR ($CDCl_3$, 101 MHz):** δ_C 168.0, 144.0, 129.5, 124.1, 114.8, 60.6, 53.6; **HRMS (ESI) m/z:** $[M+H]^+$ calcd. for $C_{11}H_{13}ClNO_4$: 258.0528; Found: 258.0522.

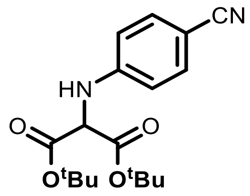

3h'

Starting from iodonium ylide **1d** (68.0 mg, 0.16 mmol) and aniline **2h** (18.9 mg, 0.16 mmol) in $CHCl_3$ (2 mL, [M] = 0.08 mol/l) using $Fe(OTf)_2$ (2.8 mg, 0.08 mmol, 5 mol%), the crude reaction mixture was purified by column chromatography on silica gel using hexane/EtOAc mixtures (80:20 to 60:40) as the eluent to afford compound **3h’** as a colourless solid (32.9 mg, 56% yield).

MW ($C_{18}H_{24}N_2O_4$): 332.17 g/mol; **Rf:** 0.67 (Hexanes/EtOAc 8:2); **IR (ATR) *ν* (cm^{-1}):** 3399, 1738, 1134; **MP (ºC):** 105 - 108; **^{1}H NMR ($CDCl_3$, 400 MHz):** **δ_H** 7.45 (d, *J* = 8.8 Hz, 2H), 6.62 (d, *J* = 8.8 Hz, 2H), 5.28 (d, *J* = 6.8 Hz, 1H), 4.54 (d, *J* = 6.8 Hz, 1H), 1.48 (s, 18H); **^{13}C{H} NMR ($CDCl_3$, 101 MHz):** δ_C 166.1, 149.1, 133.9, 113.2, 120.1, 100.7, 83.9, 61.2, 28.0; **HRMS (ESI) m/z:** [M+Na]$^+$ calcd. for $C_{18}H_{24}N_2O_4Na$: 355.1628; Found: 355.1629.

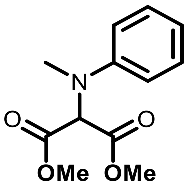

3i

Starting from iodonium ylide **1a** (53.0 mg, 0.16 mmol) and aniline **2i** (17.3 µL, 0.16 mmol) in $CHCl_3$ (2 mL, [M] = 0.08 mol/l) using $Fe(OTf)_2$ (2.8 mg, 0.08 mmol, 5 mol%), the crude reaction mixture was purified by column chromatography on silica gel using hexane/EtOAc mixtures (97:3 to 86:14) as the eluent to afford compound **3i** as an orange oil (32.0 mg, 85% yield).

MW ($C_{12}H_{15}NO_4$): 237.10 g/mol; **Rf:** 0.38 (Hexanes/EtOAc, 8:2); **IR (ATR) *ν* (cm^{-1}):** 3391, 1726, 1207; **^{1}H NMR ($CDCl_3$, 400 MHz):** δ_H 7.29 – 7.23 (m, 2H), 6.87 – 6.77 (m, 3H), 5.17 (s, 1H), 3.81 (s, 6H), 3.05 (s, 3H); **^{13}C{H} NMR ($CDCl_3$, 101 MHz):** δ_C 168.2, 148.9, 129.4, 118.9, 113.6, 65.8, 52.9, 35.8; **HRMS (ESI) m/z:** [M+H]$^+$ calcd. for $C_{12}H_{16}NO_4$: 238.1074; Found: 238.1075.

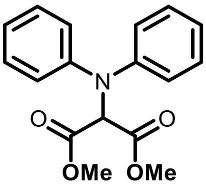

3j

Starting from iodonium ylide **1a** (53.1 mg, 0.16 mmol) and aniline **2j** (27.1 mg, 0.16 mmol) in $CHCl_3$ (2 mL, [M] = 0.08 mol/l) using $Fe(OTf)_2$ (2.8 mg, 0.08 mmol, 5 mol%), the crude reaction mixture was purified by column chromatography on silica gel using hexane/EtOAc mixtures (97:3 to 86:14) as the eluent to afford compound **3j** as a colourless oil (30.0 mg, 63% yield).

MW ($C_{17}H_{17}NO_4$): 299.12 g/mol; **Rf:** 0.54 (Hexanes/EtOAc, 8:2); **^{1}H NMR ($CDCl_3$, 400 MHz):** δ_H 7.30 – 7.25 (m, 4H), 7.07 – 6.97 (m, 6H), 5.39 (s, 1H), 3.71 (s, 6H); **^{13}C{H} NMR ($CDCl_3$, 101 MHz):** δ_C 167.9, 146.3, 129.4, 123.1, 122.2, 67.7, 53.0.

The spectroscopic data of **3j** agrees with those previously reported in the literature.[5]

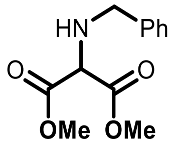

3k

Starting from iodonium ylide **1a** (53.7 mg, 0.16 mmol) and benzylamine **2k** (17.5 µL, 0.16 mmol) in $CHCl_3$ (2 mL, [M] = 0.08 mol/l) using $Fe(OTf)_2$ (2.8 mg, 0.08 mmol, 5 mol%), the crude reaction mixture was purified by column chromatography on silica gel using hexane/EtOAc mixtures (94:6 to 86:14) as the eluent to afford compound **3k** as a yellow oil (35.9 mg, 94% yield).

MW ($C_{12}H_{15}NO_4$): 237.10 g/mol; **Rf:** 0.37 (Hexanes/EtOAc, 8:2); **^{1}H NMR ($CDCl_3$, 400 MHz):** δ_H 7.35 – 7.29 (m, 4H), 7.29 – 7.23 (m, 1H), 4.10 (s, 1H), 3.81 (s, 2H), 3.77 (s, 6H), 2.22 (bs, 1H); **^{13}C{H} NMR ($CDCl_3$, 101 MHz):** δ_C 169.1, 138.7, 128.6, 128.5, 127.5, 63.9, 52.9, 51.8.

The spectroscopic data of **3k** agrees with those previously reported in the literature.[4]

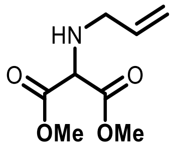

3l

Starting from iodonium ylide **1a** (53.0 mg, 0.16 mmol) and allylamine **2l** (12 µL, 0.16 mmol) in $CHCl_3$ (2 mL, [M] = 0.08 mol/l) using $Fe(OTf)_2$ (2.8 mg, 0.08 mmol, 5 mol%), the crude reaction mixture was purified by column chromatography on silica gel using hexane/EtOAc mixtures (86:14 to 60:40) as the eluent to afford compound **3l** as a yellow oil (26.7 mg, 90% yield).

MW ($C_8H_{13}NO_4$): 187.08 g/mol; **Rf:** 0.25 (Hexanes/EtOAc, 8:2); **IR (ATR) ν (cm^{-1}):** 3409, 1728; **^{1}H NMR ($CDCl_3$, 400 MHz):** δ_H 5.84 (ddt, J = 17.2, 10.2, 6.2 Hz, 1H), 5.21 (dq, J = 17.2, 1.5 Hz, 1H), 5.14 (dq, J = 10.2, 1.5 Hz, 1H), 4.10 (s, 1H), 3.78 (s, 6H), 3.27 (dt, J = 6.2, 1.5 Hz, 2H), 1.80 (bs, 1H); **^{13}C{H} NMR ($CDCl_3$, 101 MHz):** δ_C 169.1, 135.4, 117.6, 63.8, 53.0, 50.6; **HRMS (ESI) m/z:** $[M+H]^+$ calcd. for $C_8H_{14}NO_4$: 188.0917; Found: 188.0917.

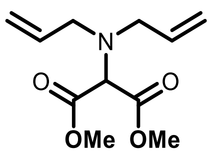

3l'

Starting from iodonium ylide **1a** (53.5 mg, 0.16 mmol) and allylamine **2l’** (20.0 µL, 0.16 mmol) in $CHCl_3$ (2 mL, [M] = 0.08 mol/l) using $Fe(OTf)_2$ (2.8 mg, 0.08 mmol, 5 mol%), the crude reaction

mixture was purified by column chromatography on silica gel using hexane/EtOAc mixtures (95:5) as the eluent to afford compound **3l'** as a colourless oil (36.8 mg, 99% yield).

MW ($C_{11}H_{17}NO_4$): 227.26 g/mol; **Rf:** 0.55 (Hexanes/EtOAc, 9:1); **IR (ATR) ν (cm^{-1}):** 1733,1115; **^{1}H NMR ($CDCl_3$, 400 MHz):** δ_H 5.81 (ddt, *J* = 16.8, 10.0, 6.4 Hz, 2H), 5.20 (dq, *J* = 16.8, 1.4 Hz, 2H), 5.14 (dq, *J* = 10.0, 1.4 Hz, 2H), 4.34 (s, 1H), 3.75 (s, 6H), 3.32 (dt, *J* = 6.4, 1.4 Hz, 4H); **^{13}C{H} NMR ($CDCl_3$, 101 MHz):** δ_C 168.8, 135.7, 118.1, 65.7, 54.6, 52.3; **HRMS (ESI) m/z:** [M+H]$^+$ calcd. for $C_{11}H_{18}NO_4$: 228.1230; Found: 228.1227.

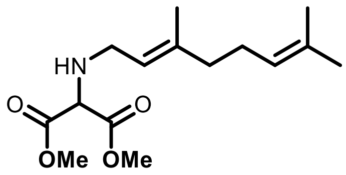

3l''

Starting from iodonium ylide **1a** (53.5 mg, 0.16 mmol) and allylamine **2l''** (30.0 µL, 0.16 mmol) in $CHCl_3$ (2 mL, [M] = 0.08 mol/l) using $Fe(OTf)_2$ (2.8 mg, 0.08 mmol, 5 mol%), the crude reaction mixture was purified by column chromatography on silica gel using hexane/EtOAc mixtures (95:5 to 90:10) as the eluent to afford compound **3l''** as a colourless oil (43.9 mg, 97% yield).

MW ($C_{15}H_{25}NO_4$): 283.37 g/mol; **Rf:** 0.17 (Hexanes/EtOAc, 9:1); **IR (ATR) ν (cm^{-1}):** 1736,1218; **^{1}H NMR ($CDCl_3$, 400 MHz):** δ_H 5.21 (t, *J* = 6.4 Hz, 1H), 5.06 (t, *J* = 6.4 Hz, 1H), 4.08 (s, 1H), 3.85 (s, 1H), 3.76 (s, 6H), 3.23 (s, 1H), 3.2 (s, 1H), 2.09 – 1.99 (m, 4H), 1.66 (s, 3H), 1.59 (d, *J* = 7.9 Hz, 6H); **^{13}C{H} NMR ($CDCl_3$, 101 MHz):** δ_C 169.3, 139.8, 131.8, 124.1, 121.3, 63.9, 52.9, 45.2, 39.7, 26.5, 25.8, 17.8, 16.3; **HRMS (ESI) m/z:** [M+H]$^+$ calcd. for $C_{15}H_{26}NO_4$: 284.1856; Found: 284.1857.

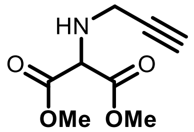

3m

Starting from iodonium ylide **1a** (53.5 mg, 0.16 mmol) and propargylamine **2m** (10.2 µL, 0.16 mmol) in $CHCl_3$ (2 mL, [M] = 0.08 mol/l) using $Fe(OTf)_2$ (2.8 mg, 0.08 mmol, 5 mol%), the crude reaction mixture was purified by column chromatography on silica gel using hexane/EtOAc mixtures (86:14 to 70:30) as the eluent to afford compound **3m** as a yellow oil (24.5 mg, 83% yield).

MW ($C_8H_{11}NO_4$): 185.07 g/mol; **Rf:** 0.40 (Hexanes/EtOAc, 7:3); **IR (ATR) ν (cm^{-1}):** 3276, 1732; **^{1}H NMR ($CDCl_3$, 400 MHz):** δ_H 4.28 (s, 1H), 3.79 (s, 6H), 3.51 (d, *J* = 2.4 Hz, 2H), 2.25 (t, *J* = 2.4

Hz, 1H), 2.14 (bs, 1H); **^{13}C{H} NMR ($CDCl_3$, 101 MHz):** δ_C 168.7, 80.3, 72.7, 63.2, 53.1, 36.8; **HRMS (ESI) m/z:** $[M+H]^+$ calcd. for $C_8H_{12}NO_4$:186.0761; Found: 186.0760.

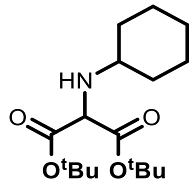

3n'

Starting from iodonium ylide **1d** (66.7 mg, 0.16 mmol) and amine **2n** (18.2 µL, 0.16 mmol) in $CHCl_3$ (2 mL, [M] = 0.08 mol/l) using $Fe(OTf)_2$ (2.8 mg, 0.08 mmol, 5 mol%), the crude reaction mixture was purified by column chromatography on silica gel using hexane/EtOAc mixtures (100:0 to 90:10) as the eluent to afford compound **3n'** as a yellow oil (43.9 mg, 87% yield).

MW ($C_{17}H_{31}NO_4$): 313.23 g/mol; **Rf:** 0.34 (Hexanes/EtOAc, 9:1); **IR (ATR) ν (cm^{-1}):** 2926, 1728; **^{1}H NMR ($CDCl_3$, 400 MHz):** δ_H 3.99 (s, 1H), 2.43 (bs, 1H), 1.86 – 1.80 (m, 2H), 1.76 – 1.68 (m, 2H), 1.62 – 1.55 (m, 1H), 1.47 (s, 18H), 1.23 – 1.10 (m, 5H);**^{13}C{H} NMR ($CDCl_3$, 101 MHz):** δ_C 168.4, 82.3, 64.0, 55.1, 33.2, 28.1, 26.1, 24.9; **HRMS (ESI) m/z:** $[M+H]^+$ calcd. for $C_{17}H_{32}NO_4$: 314.2326; Found: 314.2329.

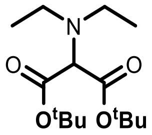

3o'

Starting from iodonium ylide **1d** (65.7 mg, 0.16 mmol) and amine **2o** (16.6 µL, 0.16 mmol) in $CHCl_3$ (2 mL, [M] = 0.08 mol/l) using $Fe(OTf)_2$ (2.8 mg, 0.08 mmol, 5 mol%), the crude reaction mixture was purified by column chromatography on silica gel using hexane/EtOAc mixtures (100:0 to 90:10) as the eluent to afford compound **3o'** as a yellow oil (35.1 mg, 77% yield).

MW ($C_{15}H_{29}NO_4$): 287.21 g/mol; **Rf:** 0.80 (Hexanes/EtOAc, 8:2); **IR (ATR) ν (cm^{-1}):** 2973, 1723; **^{1}H NMR ($CDCl_3$, 400 MHz):** δ_H 4.08 (s, 1H), 2.74 (q, *J* = 6.9 Hz, 4H), 1.48 (s, 18H), 1.10 (t, *J* = 7.1 Hz, 6H); **^{13}C{H} NMR ($CDCl_3$, 101 MHz):** δ_C 167.8, 81.9, 68.5, 45.9, 28.2, 13.9; **HRMS (ESI) m/z:** $[M+Na]^+$ calcd. for $C_{15}H_{29}NO_4Na$: 310.1989; Found: 310.1987.

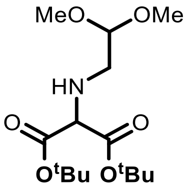

3p'

Starting from iodonium ylide **1d** (65.9 mg, 0.16 mmol) and amine **2p** (17.4 µL, 0.16 mmol) in $CHCl_3$ (2 mL, [M] = 0.08 mol/l) using $Fe(OTf)_2$ (2.8 mg, 0.08 mmol, 5 mol%), the crude reaction mixture was purified by column chromatography on silica gel using hexane/EtOAc mixtures (86:14 to 65:35) as the eluent to afford compound **3p'** as a yellow oil (43.8 mg, 97% yield).

MW ($C_{15}H_{29}NO_6$): 319.40 g/mol; **Rf:** 0.38 (Hexanes/EtOAc, 8:2); **IR (ATR) ν (cm^{-1}):** 2931, 1729; **^{1}H NMR ($CDCl_3$, 400 MHz):** δ_H 4.50 (t, J = 5.5 Hz, 1H), 3.88 (s, 1H), 3.38 (s, 6H), 2.73 (d, J = 5.5 Hz, 2H), 1.47 (s, 18H); **^{13}C{H} NMR ($CDCl_3$, 101 MHz):** δ_C 167.8, 103.9, 82.4, 66.4, 54.0, 48.7, 28.1; **HRMS (ESI) m/z:** $[M+Na]^+$ calcd. for $C_{15}H_{29}NO_6Na$: 342.1887; Found: 342.1890.

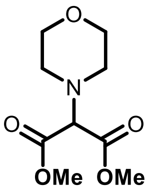

3q

Starting from iodonium ylide **1a** (52.6 mg, 0.16 mmol) and morpholine **2q** (14.0 µL, 0.16 mmol) in $CHCl_3$ (2 mL, [M] = 0.08 mol/l) using $Fe(OTf)_2$ (2.8 mg, 0.08 mmol, 5 mol%), the crude reaction mixture was purified by column chromatography on silica gel using hexane/EtOAc mixtures (90:10 to 70:30) as the eluent to afford compound **3q** as a yellow oil (23.4 mg, 68% yield).

MW ($C_9H_{15}NO_5$): 217.10 g/mol; **Rf:** 0.35 (Hexanes/EtOAc, 1:1); **^{1}H NMR ($CDCl_3$, 400 MHz):** 4.04 (s, 1H), 3.78 (s, 6H), 3.77 – 3.72 (m, 4H), 2.79 – 2.72 (m, 4H); **^{13}C{H} NMR ($CDCl_3$, 101 MHz):** δ_C 167.4, 70.9, 67.2, 52.5, 50.7.

The spectroscopic data of **3q** agrees with those previously reported in the literature.[4]

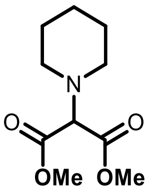

3r

Starting from iodonium ylide **1a** (53.1 mg, 0.16 mmol) and amine **2r** (15.8 µL, 0.16 mmol) in $CHCl_3$ (2 mL, [M] = 0.08 mol/l) using $Fe(OTf)_2$ (2.8 mg, 0.08 mmol, 5 mol%), the crude reaction mixture

was purified by column chromatography on silica gel using hexane/EtOAc mixtures (90:10 to 70:30) as the eluent to afford compound **3r** as a yellow oil (23.9 mg, 70% yield).

MW ($C_{10}H_{17}NO_4$): 215.12 g/mol; **Rf:** 0.33 (Hexanes/EtOAc, 1:1); **^{1}H NMR ($CDCl_3$, 400 MHz):** 4.05 (s, 1H), 3.77 (s, 6H), 2.71 – 2.61 (m, 4H), 1.68 – 1.58 (m, 4H), 1.48 – 1.42 (m, 2H); **^{13}C{H} NMR ($CDCl_3$, 101 MHz):** δ_C 167.9, 71.8, 52.3, 51.7, 26.3, 24.1.

The spectroscopic data of **3r** agrees with those previously reported in the literature.[4]

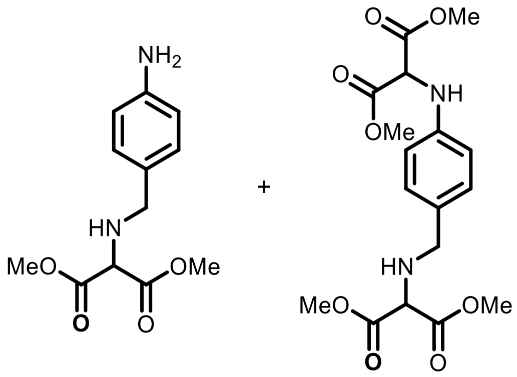

3s 3s'

Starting from iodonium ylide **1a** (54.0 mg, 0.16 mmol) and diamine **2s** (18.2 µL, 0.16 mmol) in $CHCl_3$ (2 mL, [M] = 0.08 mol/l) using $Fe(OTf)_2$ (2.8 mg, 0.08 mmol, 5 mol%), the crude reaction mixture was purified by column chromatography on silica gel using hexane/EtOAc mixtures (100:0 to 55:45) as the eluent to afford compound **3s** (9.6 mg, 31% yield) and compound **3s'** (28.3 mg, 46% yield) as yellow oils.

Compound 3s: MW ($C_{12}H_{16}N_2O_4$): 252.11 g/mol; **Rf:** 0.40 (Hexanes/EtOAc, 4:6); **IR (ATR) ν (cm^{-1}):** 3340, 2920, 1731; **^{1}H NMR ($CDCl_3$, 400 MHz):** δ_H 7.10 (d, *J* = 8.3 Hz, 2H), 6.64 (d, *J* = 8.3 Hz, 2H), 4.08 (s, 1H), 3.76 (s, 6H), 3.68 (s, 2H); **^{13}C{H} NMR ($CDCl_3$, 101 MHz):** δ_C 169.2, 145.9, 129.8, 128.6, 115.2, 63.8, 52.9, 51.4; **HRMS (ESI) m/z:** $[M+Na]^+$ calcd. for $C_{12}H_{16}N_2O_4Na$: 275.1002; Found 275.1006.

Compound 3s': MW ($C_{17}H_{22}N_2O_8$): 382.14 g/mol; **Rf:** 0.57 (Hexanes/EtOAc, 4:6); **IR (ATR) ν (cm^{-1}):** 2919, 1730; **^{1}H NMR ($CDCl_3$, 400 MHz):** δ_H 7.15 (d, *J* = 8.3 Hz, 2H), 6.61 (d, *J* = 8.3 Hz, 2H), 4.87 – 4.75 (m, 2H), 4.07 (s, 1H), 3.82 (s, 6H), 3.76 (s, 6H), 3.68 (s, 2H); **^{13}C{H} NMR ($CDCl_3$, 101 MHz):** δ_C 169.1, 168.3, 144.8, 129.9, 129.2, 113.8, 113.7, 63.8, 60.7, 53.5, 52.9, 51.3; **HRMS (ESI) m/z:** $[M+Na]^+$ calcd. for $C_{17}H_{22}N_2O_8Na$: 405.1268; Found: 405.1263.

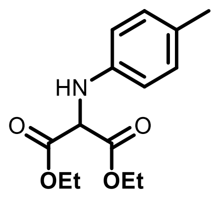

3t

Starting from iodonium ylide **1c** (47.1 mg, 0.13 mmol) and aniline **2a** (13.9 mg, 0.13 mmol) in $CHCl_3$ (2 mL, [M] = 0.08 mol/l) using $Fe(OTf)_2$ (2.8 mg, 0.08 mmol, 5 mol%), the crude reaction mixture was purified by column chromatography on silica gel using hexane/EtOAc mixtures (94:6 to 86:14) as the eluent to afford compound **3t** as a colourless solid (30.4 mg, 88% yield).

MW ($C_{14}H_{19}NO_4$): 265.13 g/mol; **Rf:** 0.71 (Hexanes/EtOAc, 8:2); **^{1}H NMR ($CDCl_3$, 400 MHz):** δ_H 7.00 (d, J = 8.3 Hz, 2H), 6.59 (d, J = 8.3 Hz, 2H), 4.76 – 4.68 (m, 2H), 4.27 (q, J = 7.1 Hz, 4H), 2.24 (s, 3H), 1.28 (t, J = 7.1 Hz, 6H); **^{13}C{H} NMR ($CDCl_3$, 101 MHz):** δ_C 168.0, 143.3, 130.0, 128.5, 113.9, 62.5, 61.4, 20.6, 14.2.

The spectroscopic data of **3t** agrees with those previously reported in the literature.[3]

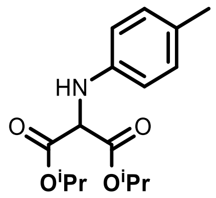

3u

Starting from iodonium ylide **1b** (62.6 mg, 0.16 mmol) and aniline **2a** (17.2 mg, 0.16 mmol) in $CHCl_3$ (2 mL, [M] = 0.08 mol/l) using $Fe(OTf)_2$ (2.8 mg, 0.08 mmol, 5 mol%), the crude reaction mixture was purified by column chromatography on silica gel using hexane/EtOAc mixtures (94:6) as the eluent to afford compound **3u** as a colourless solid (41.5 mg, 88% yield).

MW ($C_{16}H_{23}NO_4$): 293.16 g/mol; **Rf:** 0.76 (Hexanes/EtOAc, 8:2); **IR (ATR) ν (cm^{-1}):** 3394, 1723, 1100; **MP (ºC):** 62 – 65; **^{1}H NMR ($CDCl_3$, 400 MHz):** δ_H 6.99 (d, J = 8.2 Hz, 2H), 6.58 (d, J = 8.2 Hz, 2H), 5.11 (hept, J = 6.3 Hz, 2H), 4.72 – 4.63 (m, 2H), 2.23 (s, 3H), 1.28 (d, J = 6.3 Hz, 6H), 1.24 (d, J = 6.3 Hz, 6H); **^{13}C{H} NMR ($CDCl_3$, 101 MHz):** δ_C 167.6, 143.4, 130.0, 128.3, 113.9, 70.2, 61.6, 21.8, 21.7, 20.6; **HRMS (ESI) m/z:** $[M+H]^+$ calcd. for $C_{16}H_{24}NO_4$: 294.1700; Found: 294.1702.

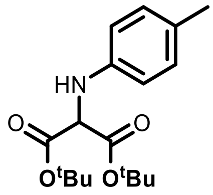

3v

Starting from iodonium ylide **1d** (66.6 mg, 0.16 mmol) and aniline **2a** (17.2 mg, 0.16 mmol) in $CHCl_3$ (2 mL, [M] = 0.08 mol/l) using $Fe(OTf)_2$ (2.8 mg, 0.08 mmol, 5 mol%), the crude reaction mixture was purified by column chromatography on silica gel using hexane/EtOAc mixtures (97:3) as the eluent to afford compound **3v** as a colourless solid (41.5 mg, 87% yield).

MW ($C_{18}H_{27}NO_4$): 321.19 g/mol; **Rf:** 0.53 (Hexanes/EtOAc, 9:1); **IR (ATR) ν (cm^{-1}):** 3400, 1716, 1142; **MP (ºC):** 71 – 74; **^{1}H NMR ($CDCl_3$, 400 MHz):** δ_H 6.98 (d, J = 8.3 Hz, 2H), 6.58 (d, J = 8.3 Hz, 2H), 4.66 (bs, 1H), 4.51 (s, 1H), 2.23 (s, 3H), 1.47 (s, 18H); **^{13}C{H} NMR ($CDCl_3$, 101 MHz):** δ_C 167.3, 143.7, 129.9, 127.9, 113.9, 82.9, 62.6, 28.0, 20.6; **HRMS (ESI) m/z:** $[M+H]^+$ calcd. for $C_{18}H_{28}NO_4$: 322.2013; Found: 322.2019.

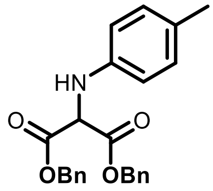

3w

Starting from iodonium ylide **1e** (78.0 mg, 0.16 mmol) and aniline **2a** (17.2 mg, 0.16 mmol) in $CHCl_3$ (2 mL, [M] = 0.08 mol/l) using $Fe(OTf)_2$ (2.8 mg, 0.08 mmol, 5 mol%), the crude reaction mixture was purified by column chromatography on silica gel using hexane/EtOAc mixtures (97:3 to 94:6) as the eluent to afford compound **3w** as a colourless solid (55.5 mg, 89% yield).

MW ($C_{24}H_{23}NO_4$): 389.16 g/mol; **Rf:** 0.60 (Hexanes/EtOAc 8:2); **IR (ATR) ν (cm^{-1}):** 3381, 1750, 1164; **MP (ºC):** 89 – 92; **^{1}H NMR ($CDCl_3$, 400 MHz):** δ_H 7.38 – 7.30 (m, 6H), 7.28 – 7.24 (m, 4H), 7.04 – 6.96 (m, 2H), 6.63 – 6.55 (m, 2H), 5.20 (s, 4H), 4.88 (s, 1H), 4.74 (bs, 1H), 2.26 (s, 3H); **^{13}C{H} NMR ($CDCl_3$, 101 MHz):** δ_C 167.7, 143.1, 135.0, 130.0, 128.8, 128.7, 128.6, 128.4, 114.0, 68.1, 61.4, 20.6; **HRMS (ESI) m/z:** $[M+H]^+$ calcd. for $C_{24}H_{24}NO_4$: 390.1700; Found: 390.1700.

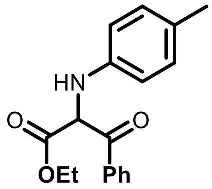

3x

Starting from iodonium ylide **1f** (63.0 mg, 0.16 mmol) and aniline **2a** (17.4 mg, 0.16 mmol) in $CHCl_3$ (2 mL, [M] = 0.08 mol/l) using $Fe(OTf)_2$ (2.8 mg, 0.08 mmol, 5 mol%), the crude reaction mixture was purified by column chromatography on silica gel using hexane/EtOAc mixtures (95:5) as the eluent to afford compound **3x** as a colourless solid (18.3 mg, 38% yield).

MW ($C_{18}H_{19}NO_3$): 297.35 g/mol; **Rf:** 0.37 (Hexanes/EtOAc 9:1); **IR (ATR) ν (cm^{-1}):** 1737,1263; **MP (ºC):** 80-82; **^{1}H NMR ($CDCl_3$, 400 MHz):** δ_H 8.16 – 8.09 (m, 2H), 7.66 – 7.57 (m, 1H), 7.55 – 7.46 (m, 2H), 7.05 – 6.96 (m, 2H), 6.69 – 6.63 (m, 2H), 5.62 (d, J = 6.2 Hz, 1H), 5.11 (d, J = 6.2 Hz, 1H), 4.14 (q, J = 7.2 Hz, 2H), 2.24 (s, 3H), 1.10 (t, J = 7.2 Hz, 3H).; **^{13}C{H} NMR ($CDCl_3$, 101 MHz):** δ_C 192.5, 168.6, 143.5, 134.5, 134.3, 130.1, 129.3, 128.9, 128.3, 114.1, 64.1, 62.4, 20.6, 14.0; **HRMS (ESI) m/z:** $[M+H]^+$ calcd. for $C_{18}H_{20}NO_3$: 298.1438; Found: 298.1438.

S3. Mechanistic studies

a) Reaction in absence of the amine

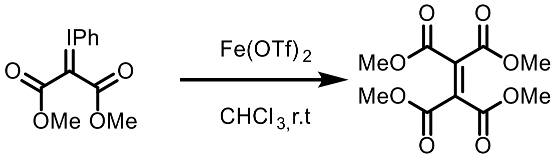

1a 64 % NMR yield **4**

A mixture of iodonium ylide **1a** (53.4 mg, 0.16 mmol, 1 equiv.) and $Fe(OTf)_2$ (2.8 mg, 0.08 mmol, 5 mol%) was placed into a 10-mL vial, and chloroform (2 mL, [M] = 0.08 mol/l) was then added. Upon addition of the chloroform, immediate solubilization of all solids indicated the termination of the reaction. The crude mixture was then filtered through a silica plug to remove impurities and the solvent was evaporated under reduced pressure. The crude reaction mixture was then analyzed by ^{1}H-NMR, using 1,3,5-trimethoxybenzene as internal standard, giving a 64% NMR yield.

^{1}H NMR ($CDCl_3$, 400 MHz): δ_H 3.89 (s, 12H).

S4. References

[1] Epping, R.F.J.; Hoeskma, M.M.; Bobylev, E.O.; Mathew, S.; de Bruin, B. *Nat. Chem*. **2022**, *14*, 550-557.
[2] Goudreau, S.R.; Marcoux, D.; Charette, A.B. *J. Org. Chem.* **2009**, *74*, 470–473.
[3] Ramakrishna, K.; Sivasankar, C. *Org. Biomol. Chem.* **2017**,*15*, 2392-2396.
[4] Zhong, Z.; Besnard, C.; Lacour, J. *Org. Lett*. **2024**, *26*, 983–987.
[5] Yang, M.; Wang, X.; Li, H.; Livant, P. *J. Org. Chem.* **2001**, *66*, 6729–6733.

S5. NMR SPECTRA

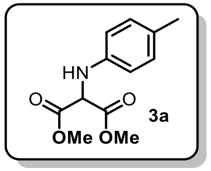

^{1}H NMR (400 MHz, $CDCl_3$)

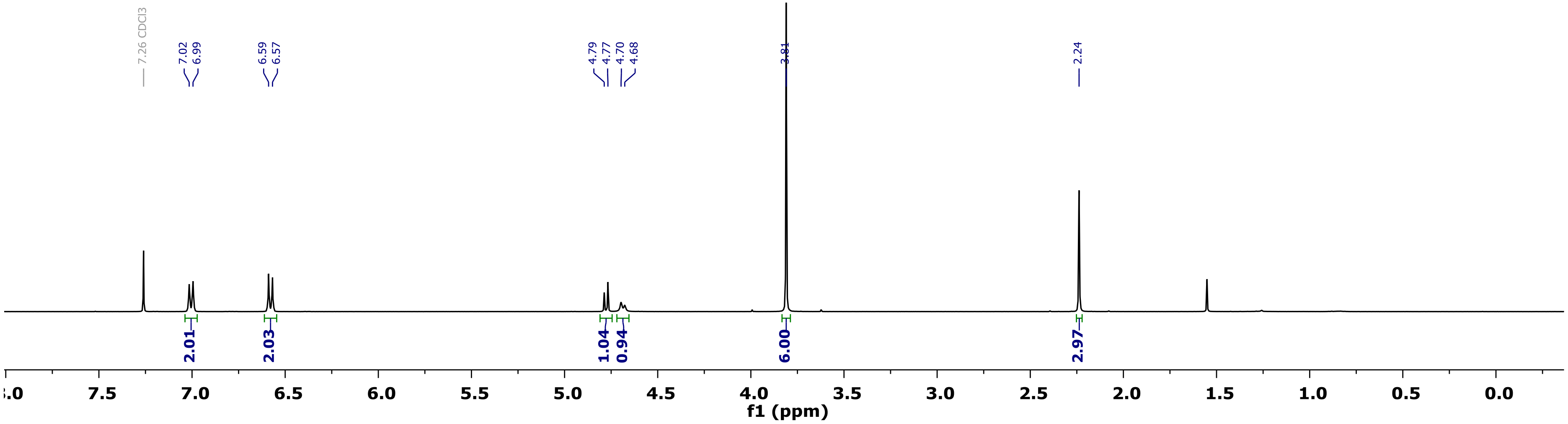

$^{13}C\{H\}$ NMR (101 MHz, $CDCl_3$)

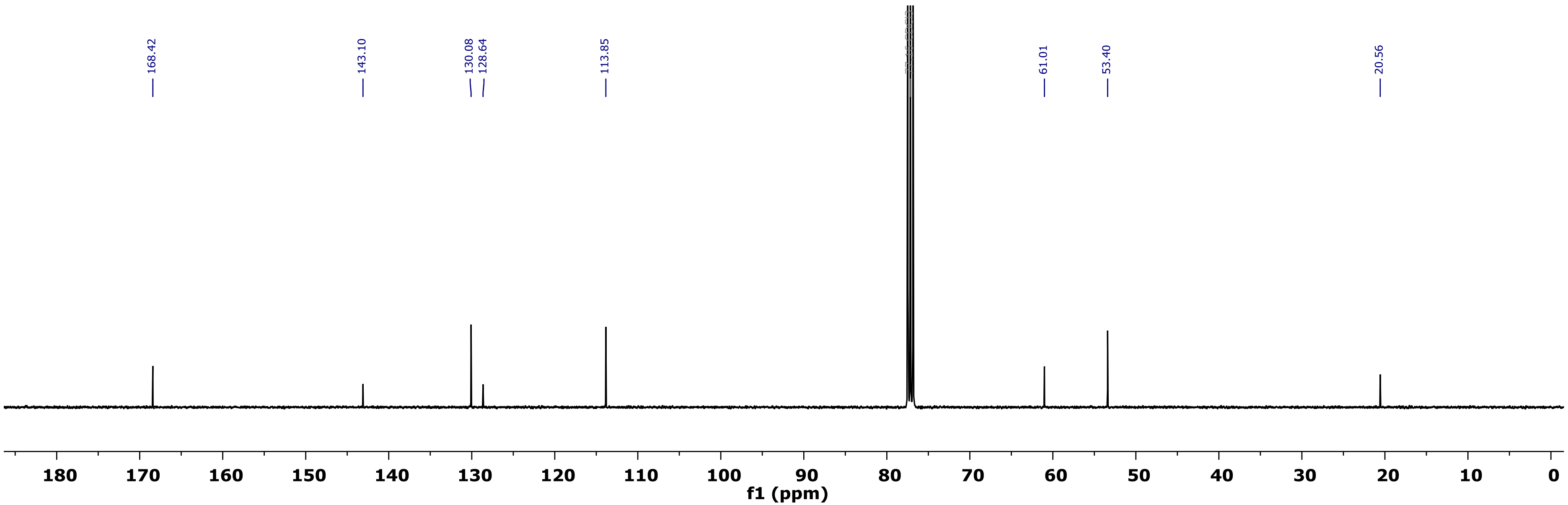

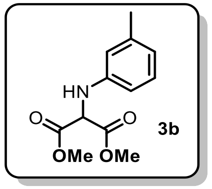

3b

^{1}H NMR (400 MHz, $CDCl_3$)

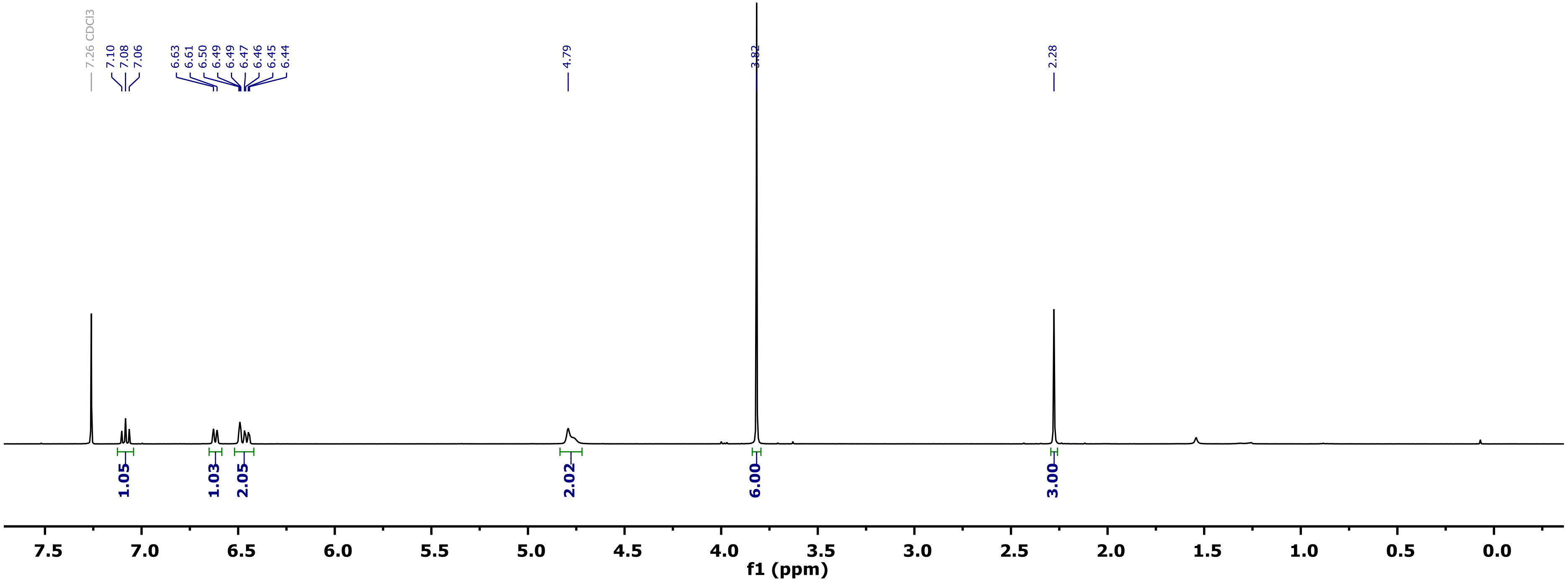

$^{13}C\{H\}$ NMR (101 MHz, $CDCl_3$)

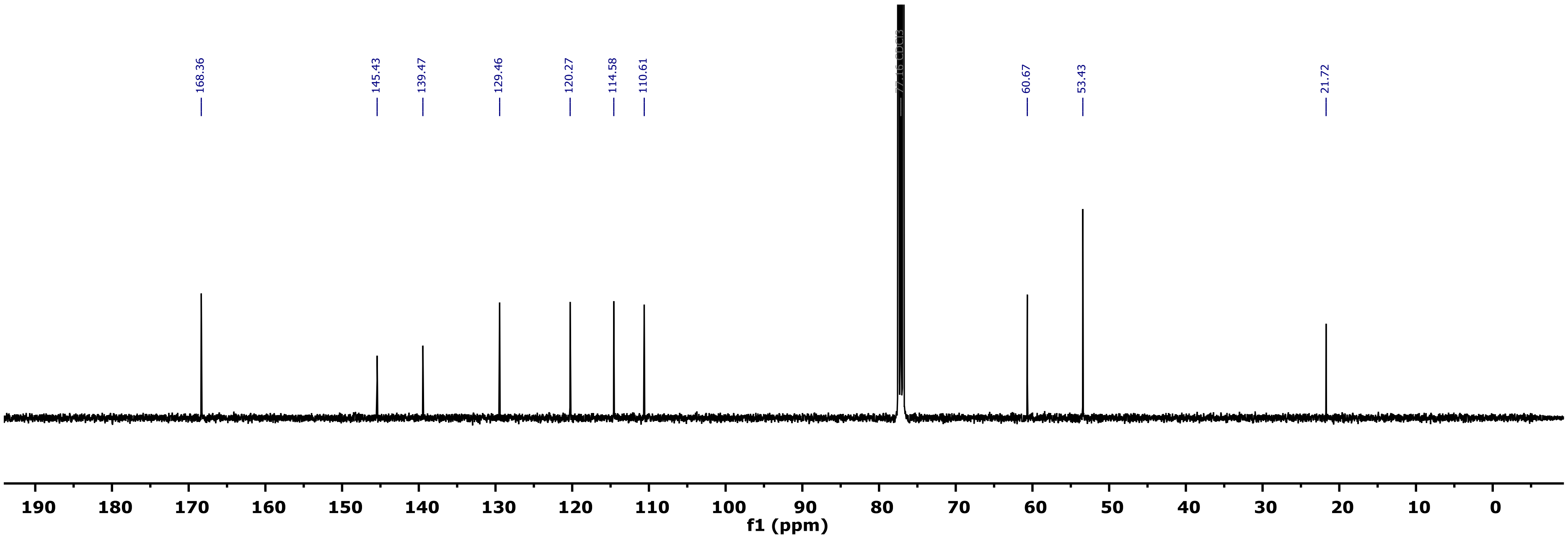

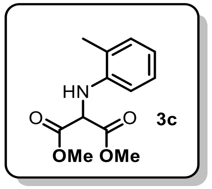

^{1}H NMR (400 MHz, $CDCl_3$)

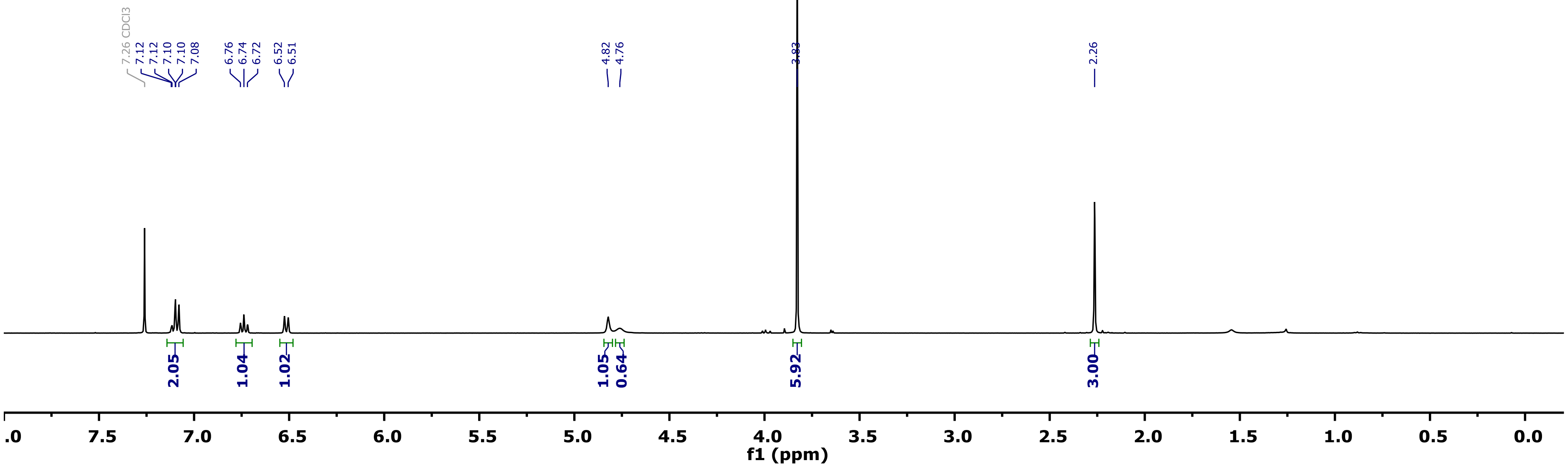

$^{13}C\{H\}$ NMR (101 MHz, $CDCl_3$)

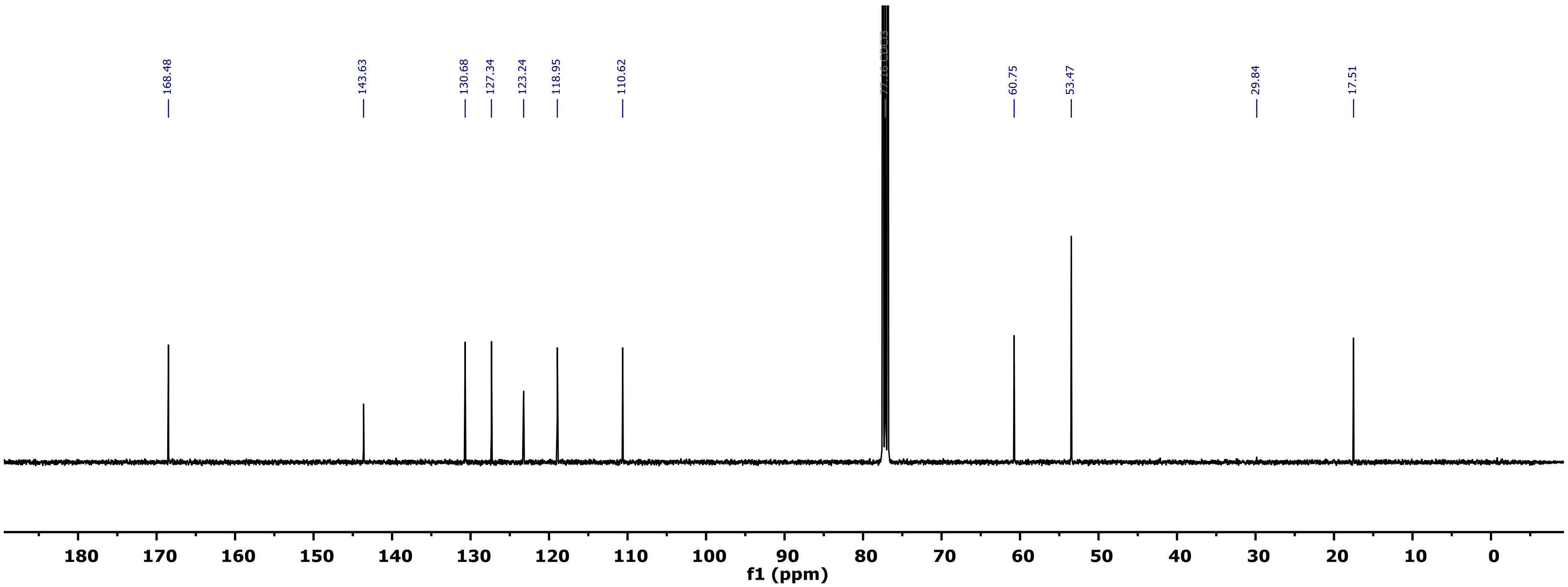

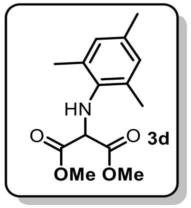

^{1}H NMR (400 MHz, $CDCl_3$)

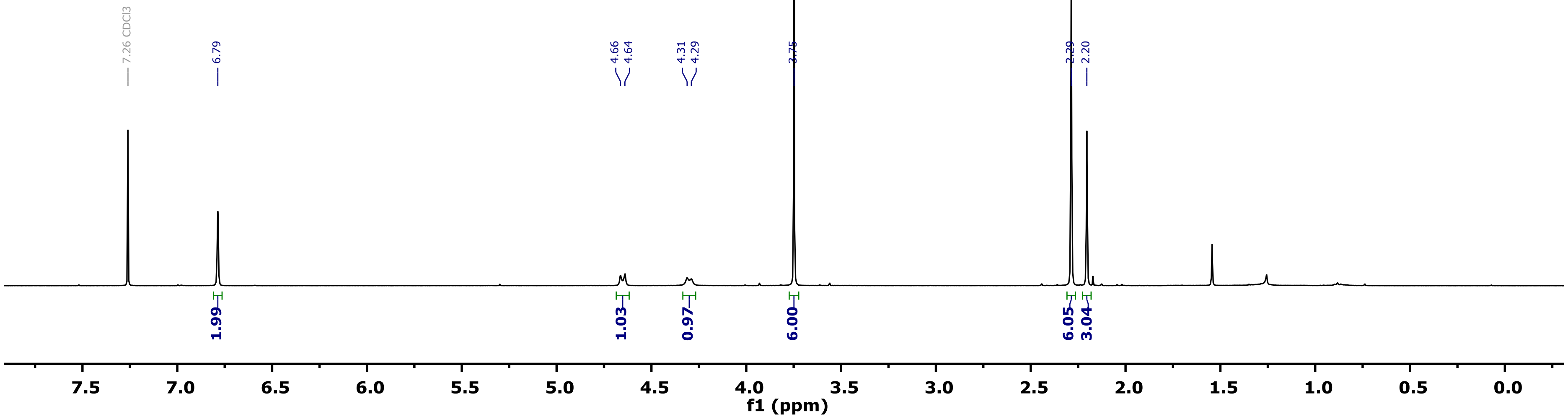

$^{13}C\{H\}$ NMR (101 MHz, $CDCl_3$)

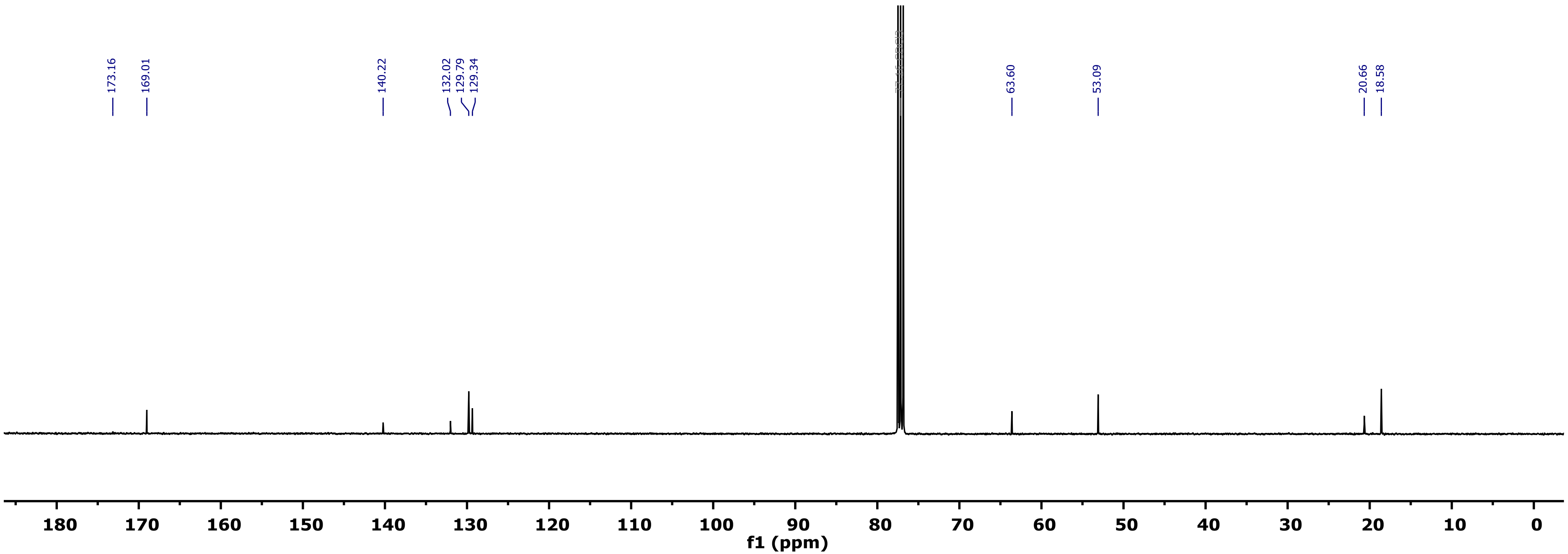

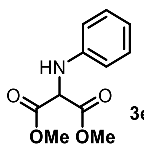

3e

^{1}H NMR (400 MHz, CDCl$_3$)

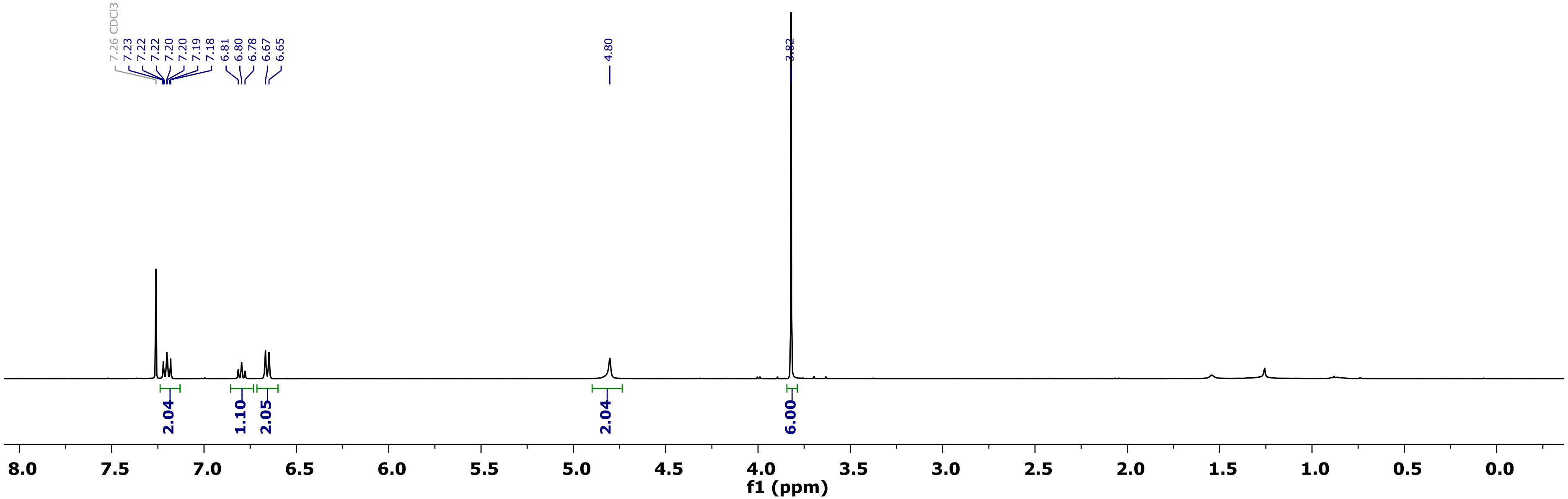

$^{13}C\{H\}$ NMR (101 MHz, $CDCl_3$)

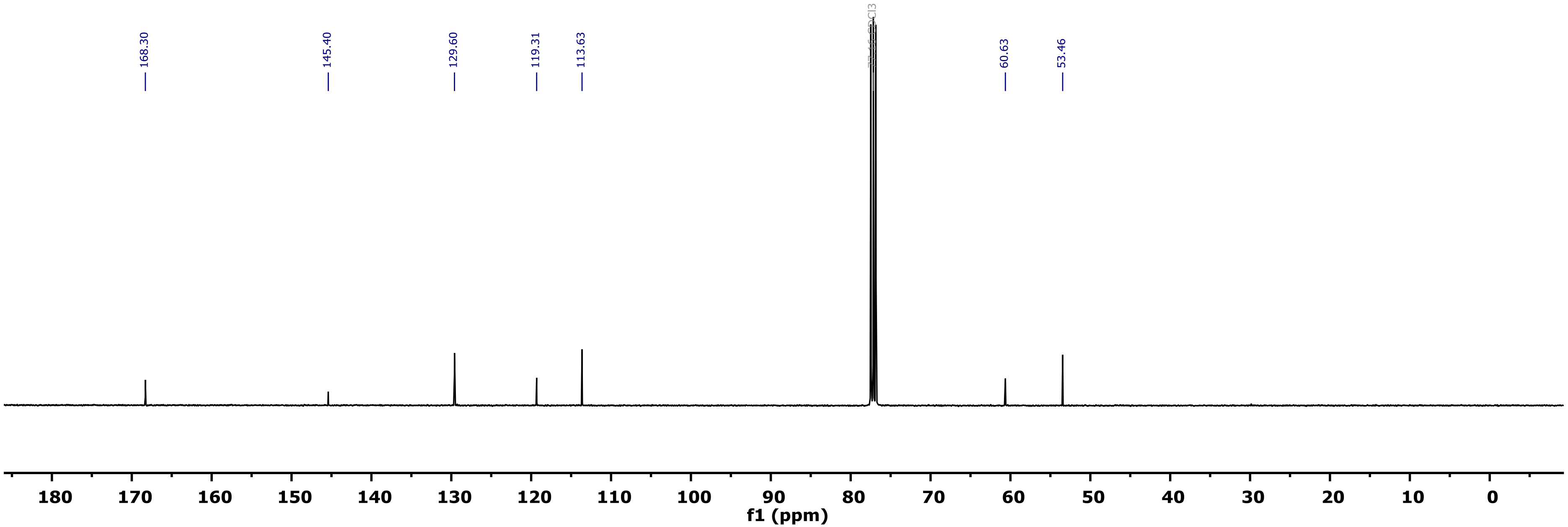

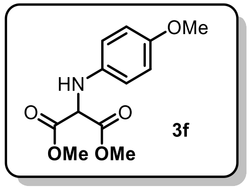

^1H NMR (400 MHz, $CDCl_3$)

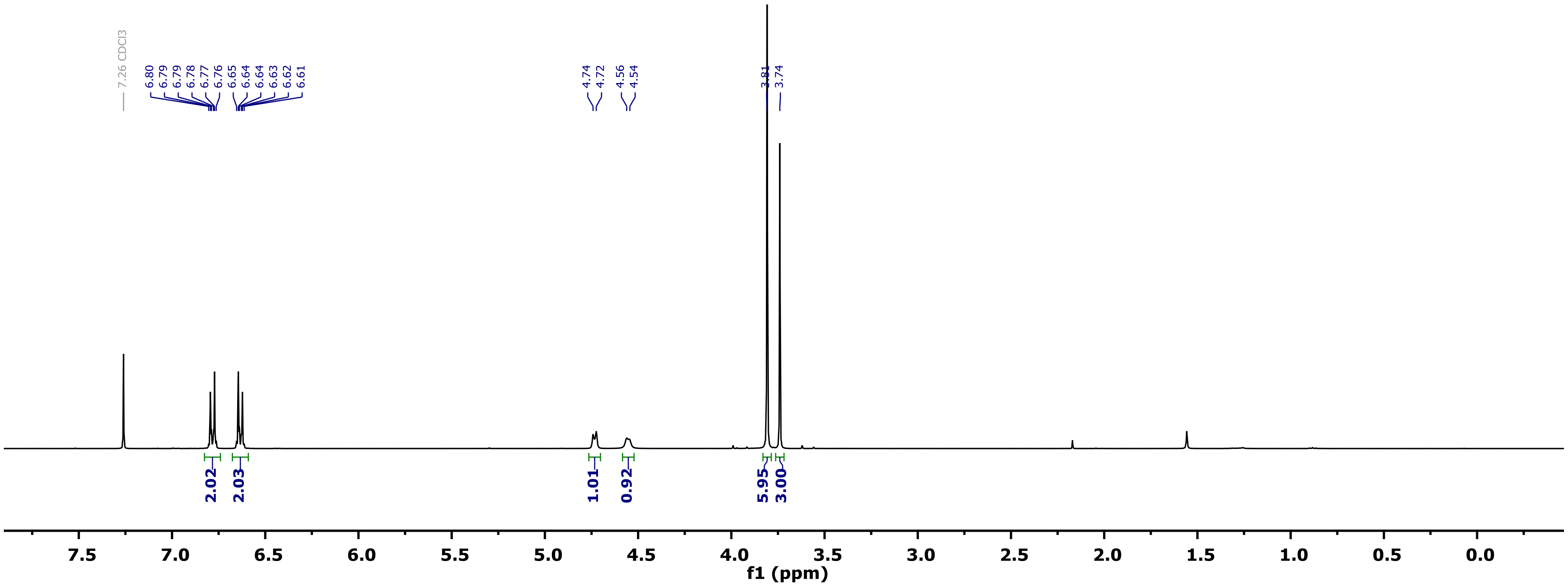

$^{13}C\{H\}$ NMR (101 MHz, $CDCl_3$)

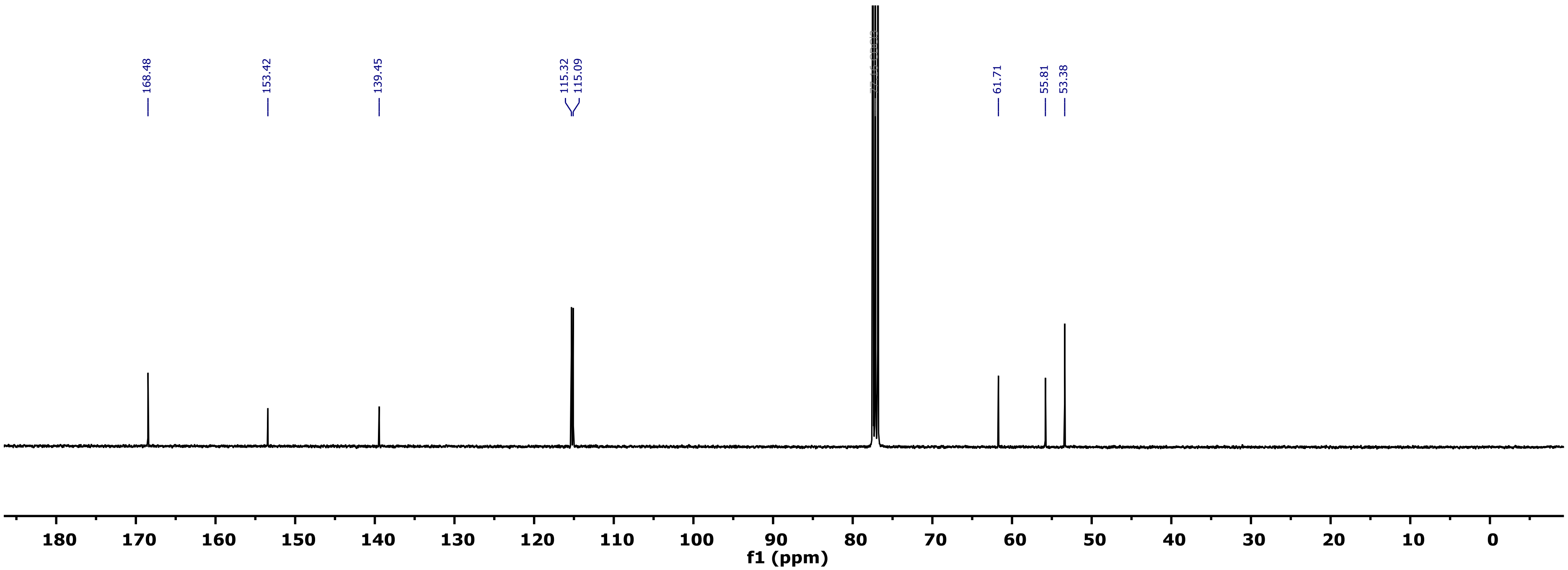

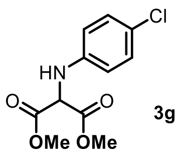

3g

^{1}H NMR (400 MHz, $CDCl_3$)

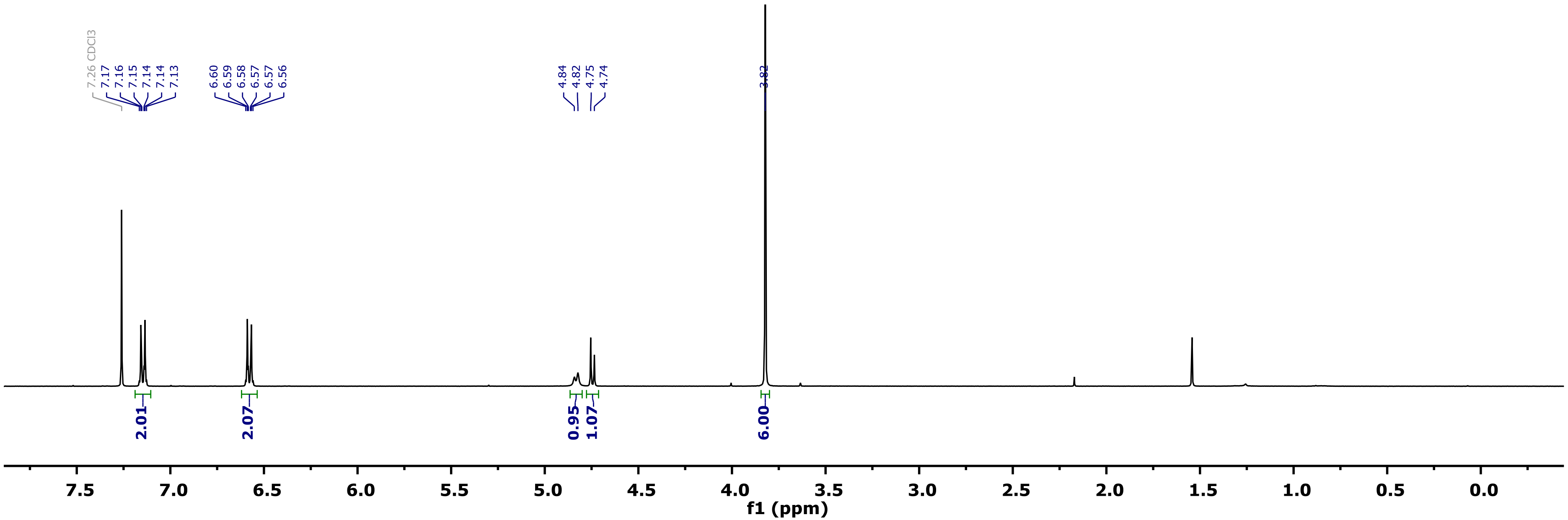

$^{13}C\{H\}$ NMR (101 MHz, $CDCl_3$)

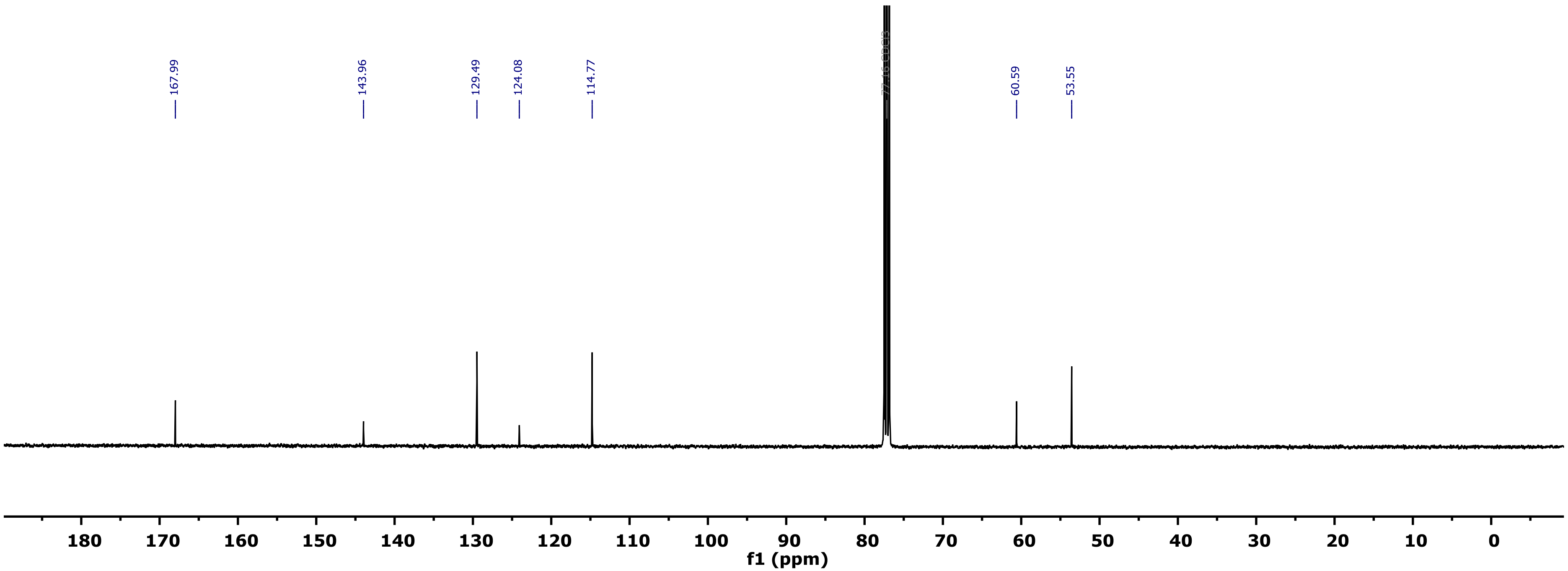

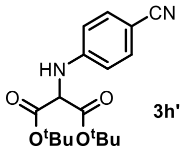

3h'

^{1}H NMR (400 MHz, $CDCl_3$)

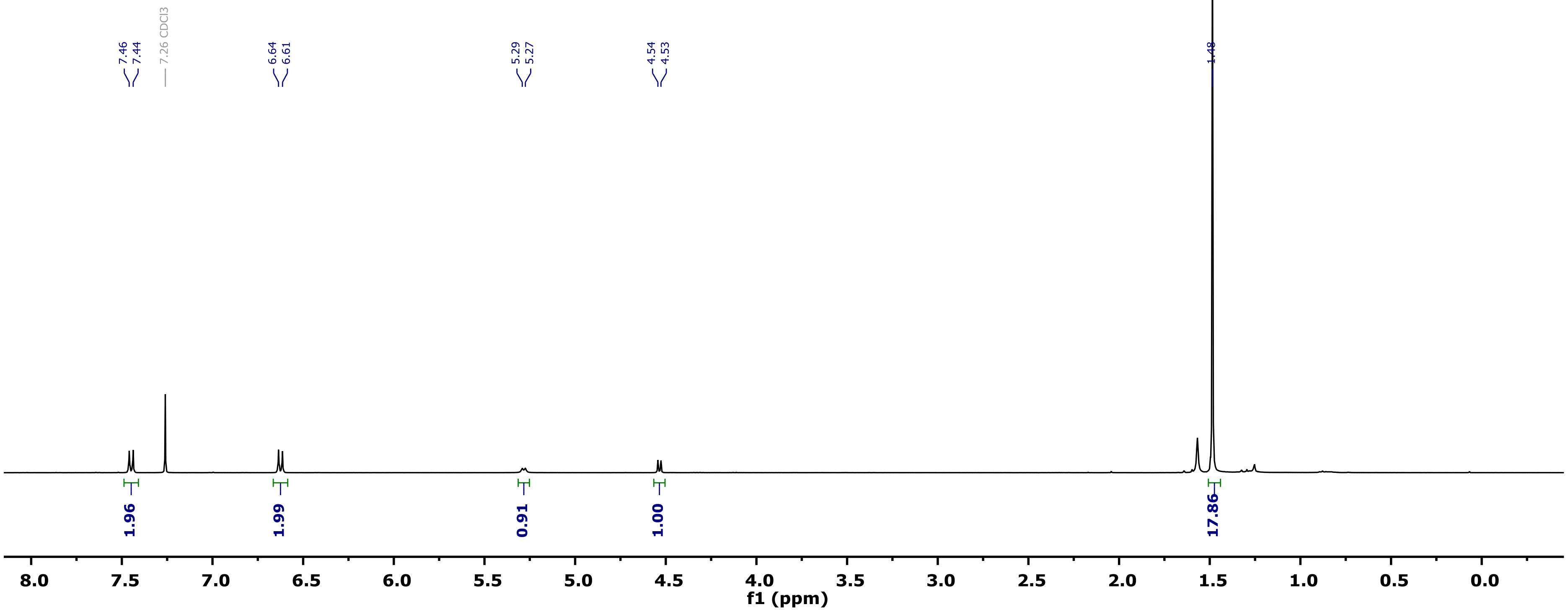

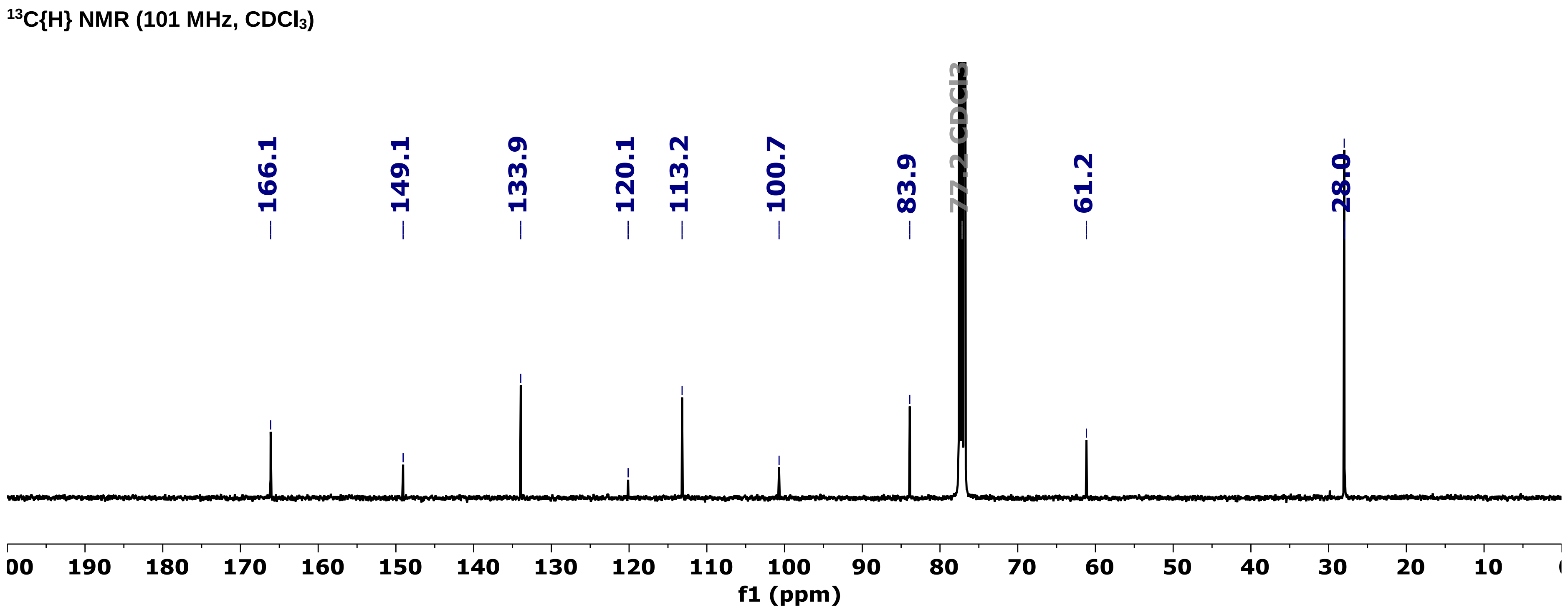

^{13}C{H} NMR (101 MHz, CDCl$_3$)
166.1
149.1
133.9
120.1
113.2
100.7
83.9
77.2 CDCl3
61.2
28.0
200
190
180
170
160
150
140
130
120
110
100
90
80
70
60
50
40
30
20
10
0
f1 (ppm)

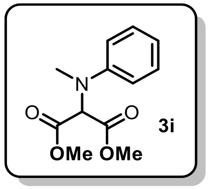

^{1}H NMR (400 MHz, $CDCl_3$)

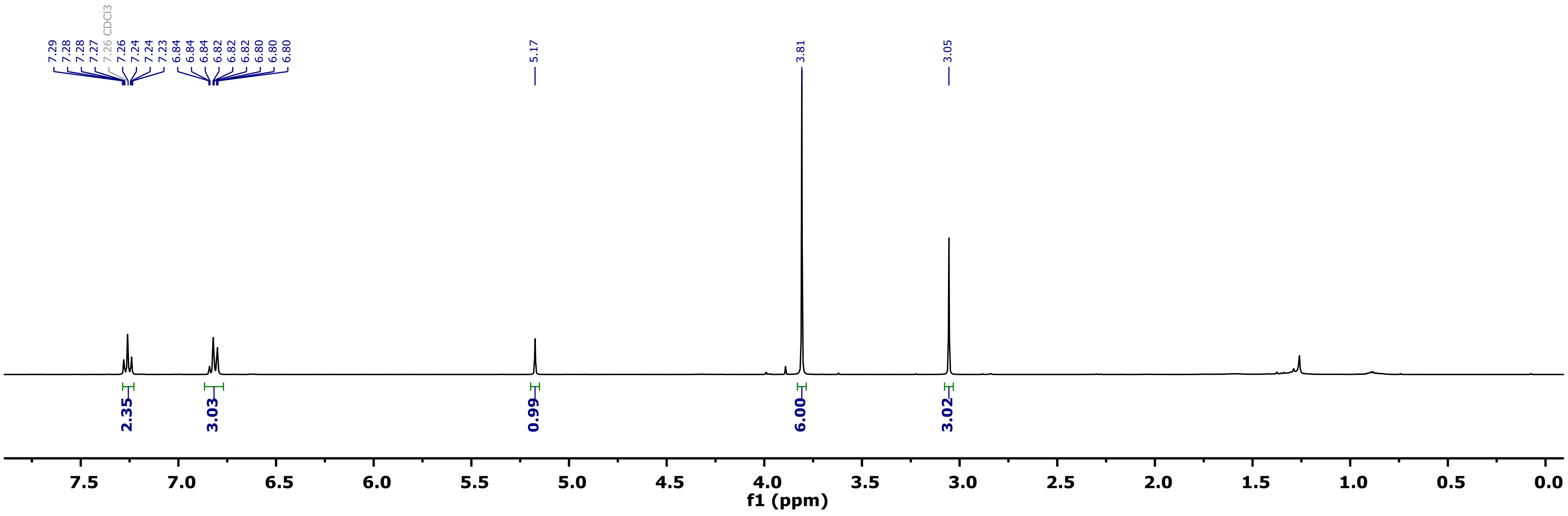

$^{13}C\{H\}$ NMR (101 MHz, $CDCl_3$)

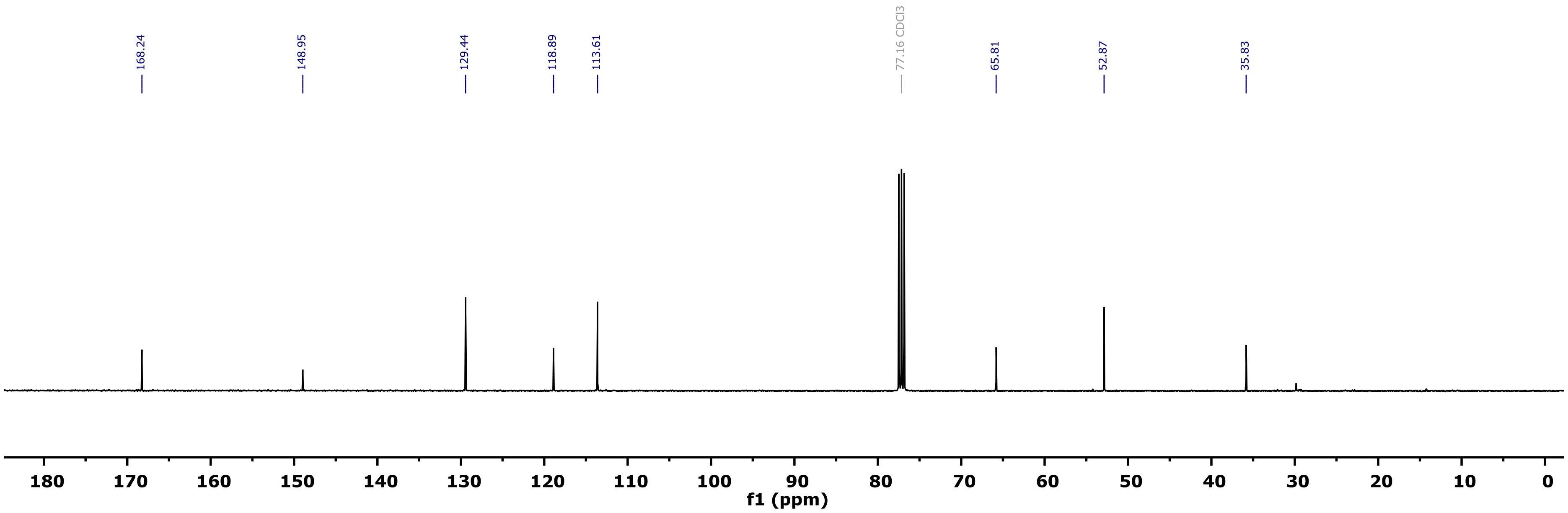

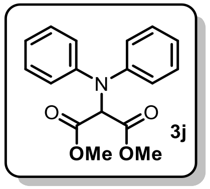

3j

^{1}H NMR (400 MHz, $CDCl_3$)

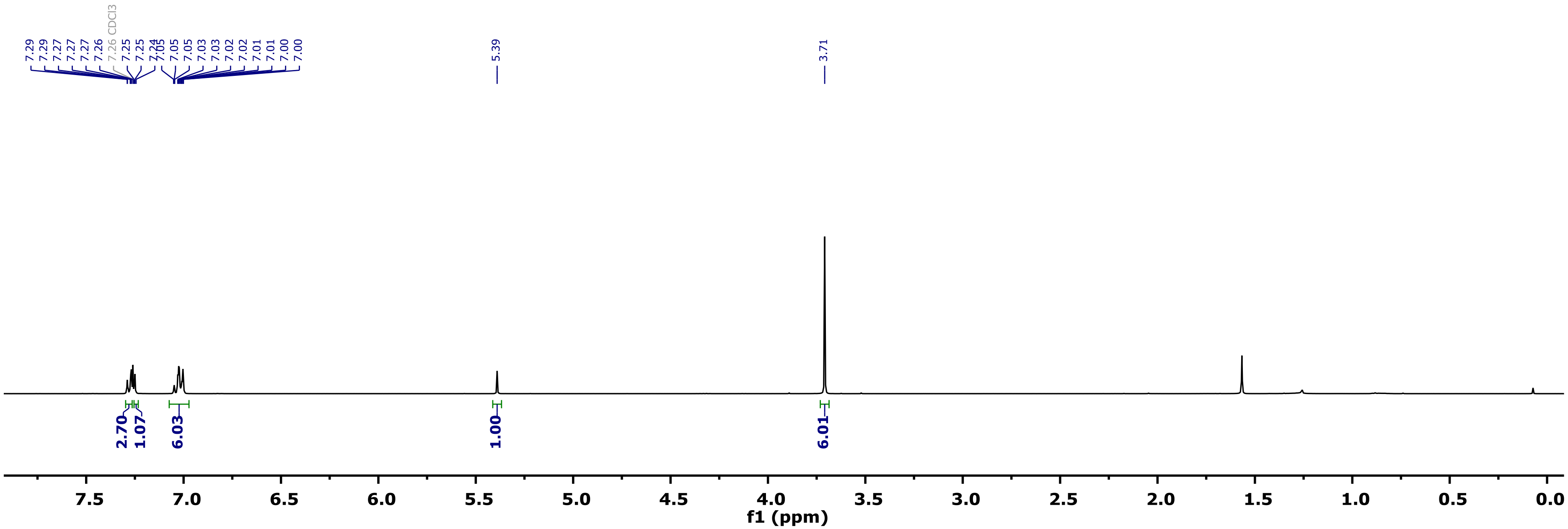

$^{13}C\{H\}$ NMR (101 MHz, $CDCl_3$)

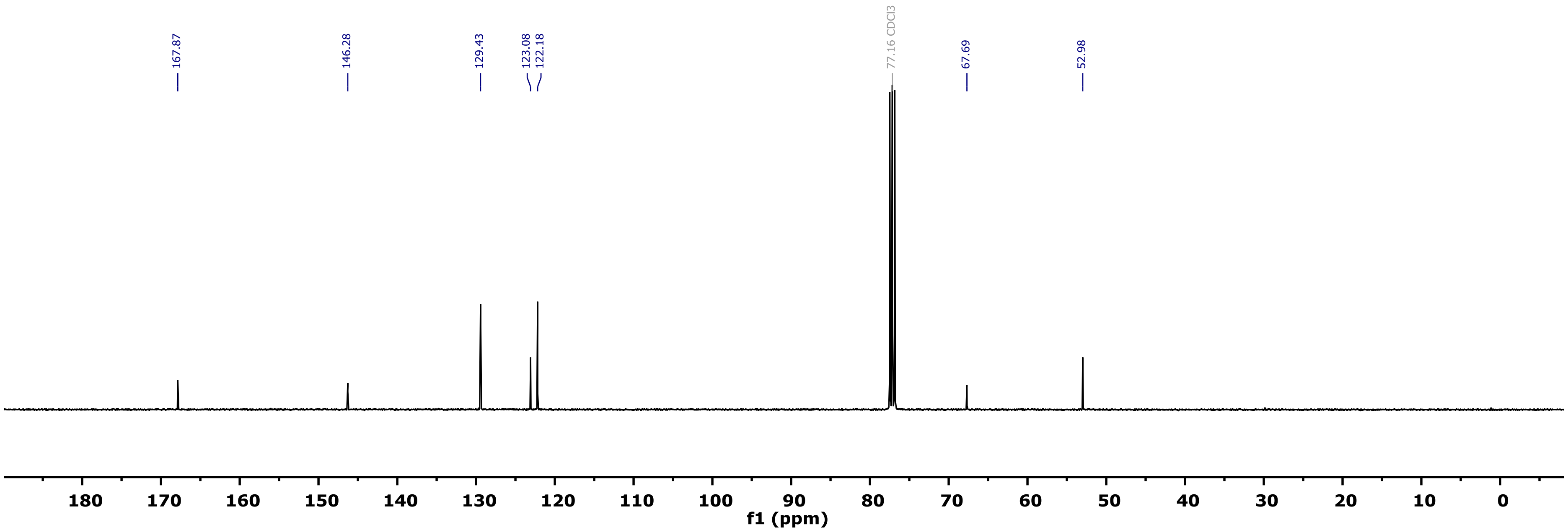

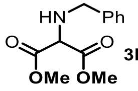

3k

^{1}H NMR (400 MHz, CDCl$_3$)

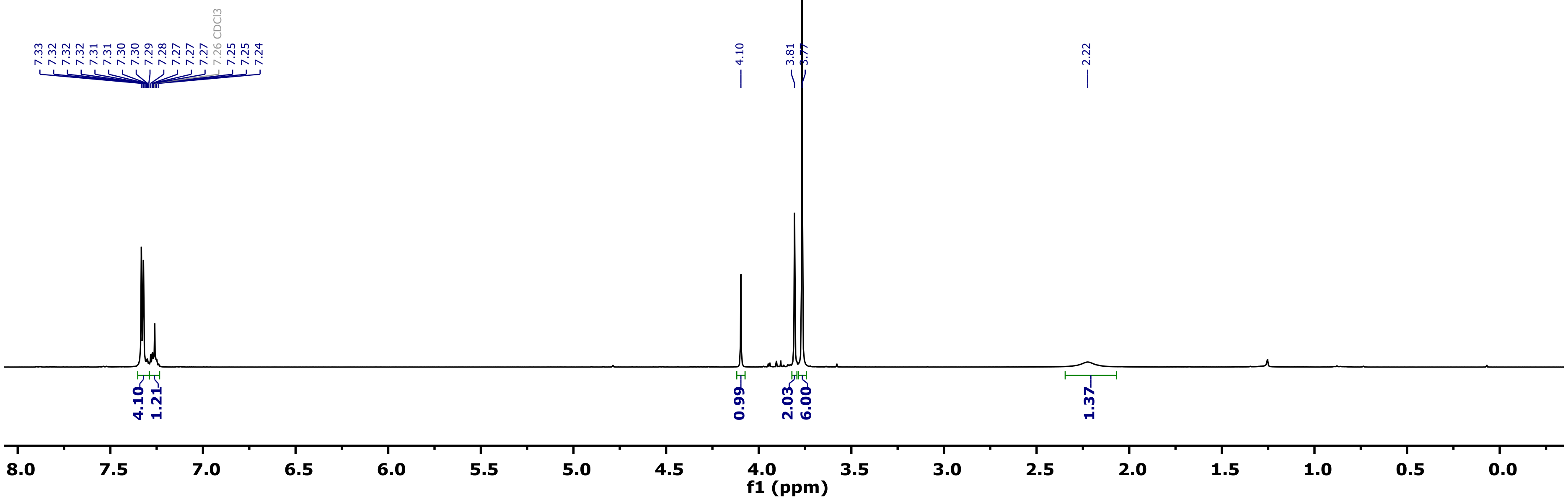

$^{13}C\{H\}$ NMR (101 MHz, $CDCl_3$)

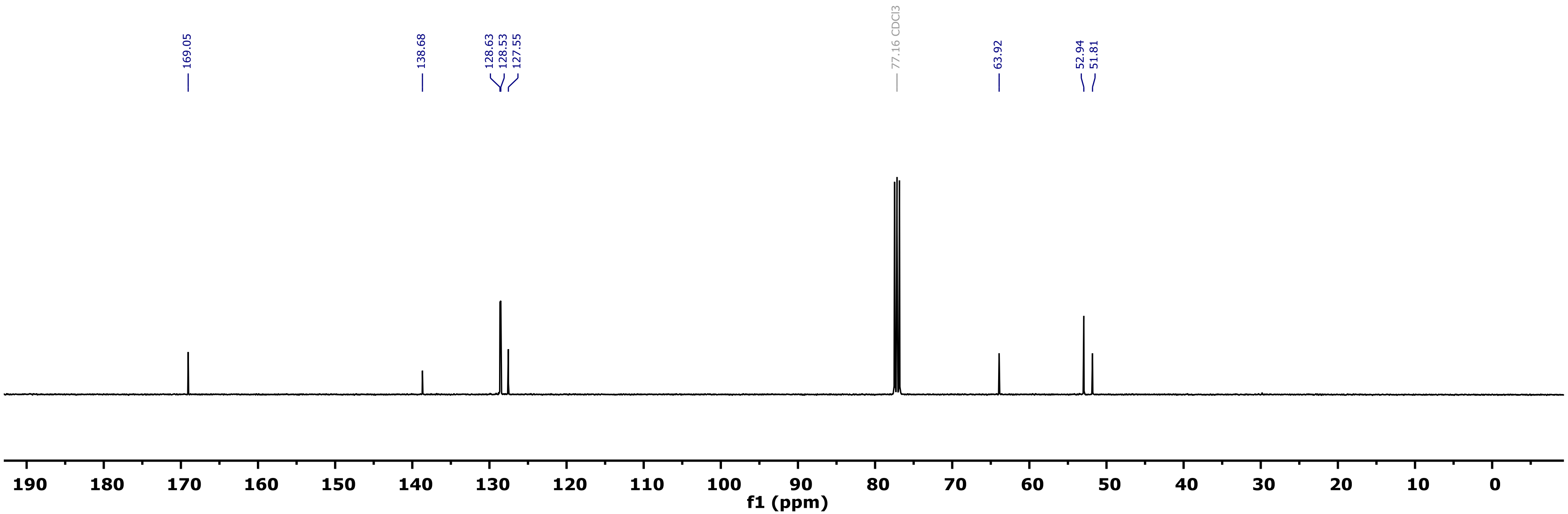

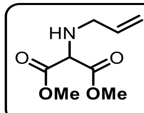

3l

¹H NMR (400 MHz, $CDCl_3$)

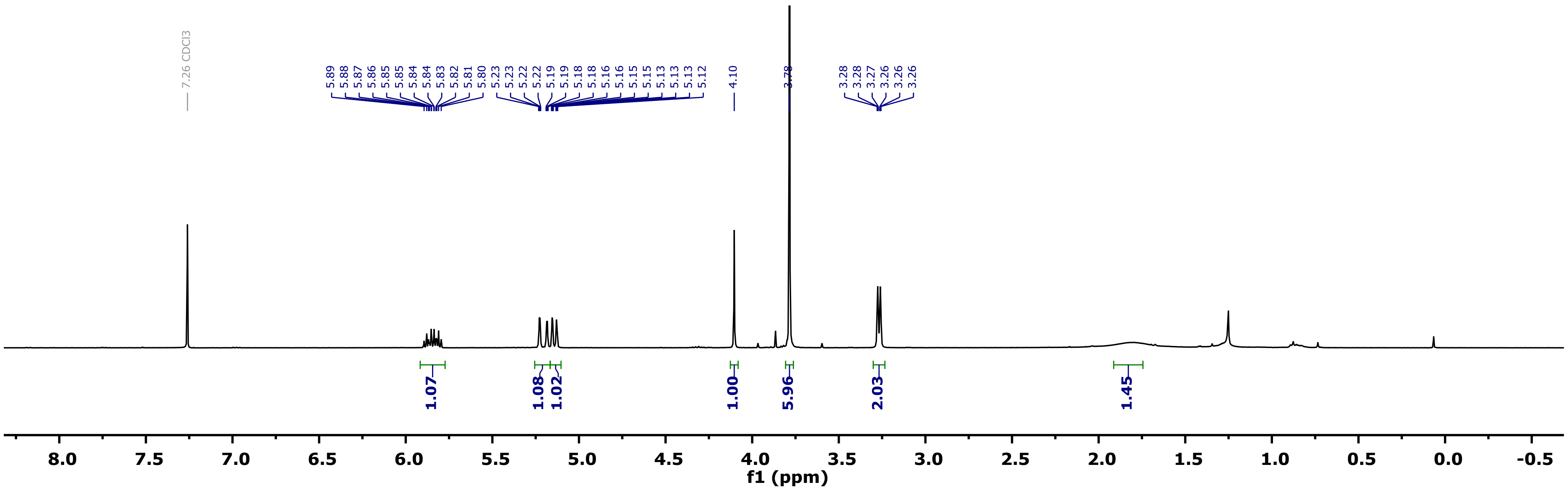

$^{13}C\{H\}$ NMR (101 MHz, $CDCl_3$)

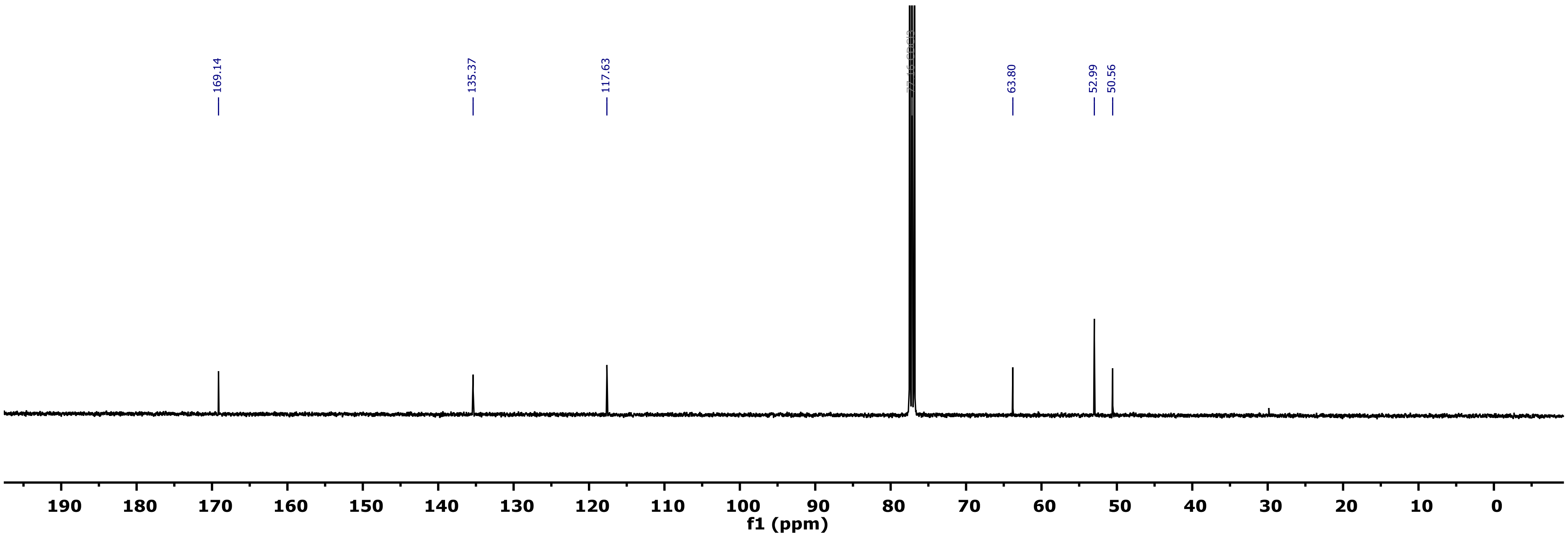

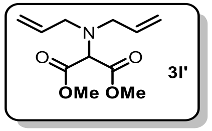

^{1}H NMR (400 MHz, $CDCl_3$)

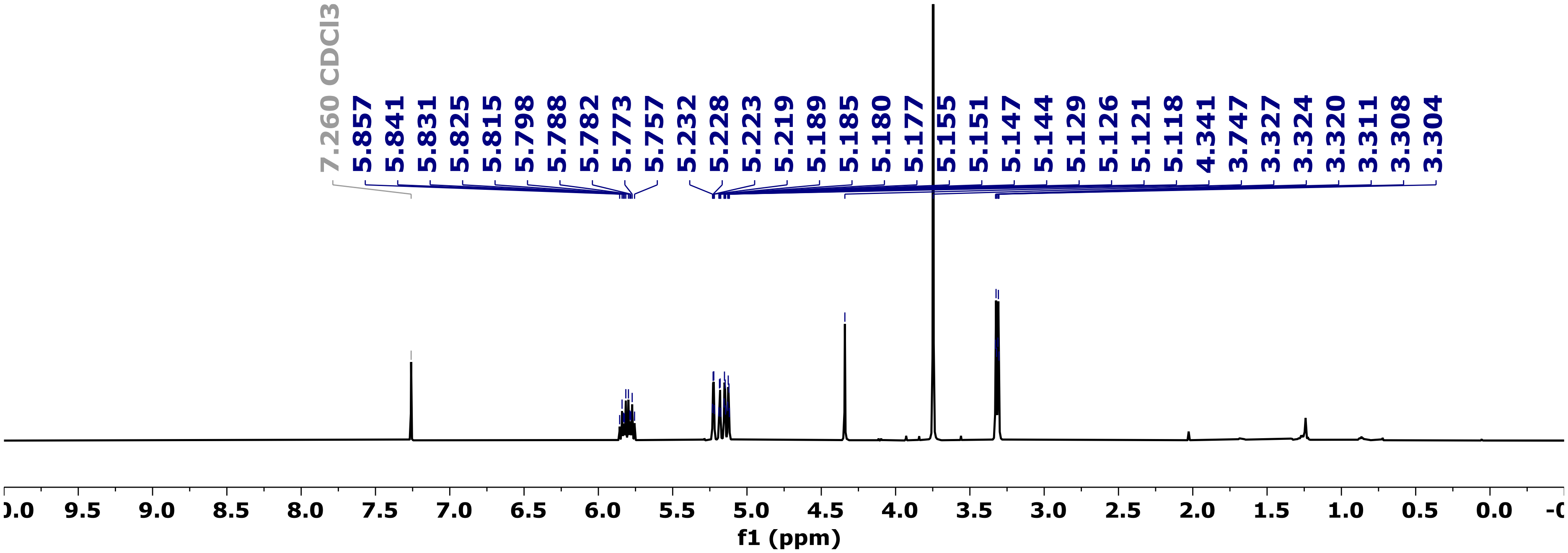

$^{13}C\{H\}$ NMR (101 MHz, $CDCl_3$)

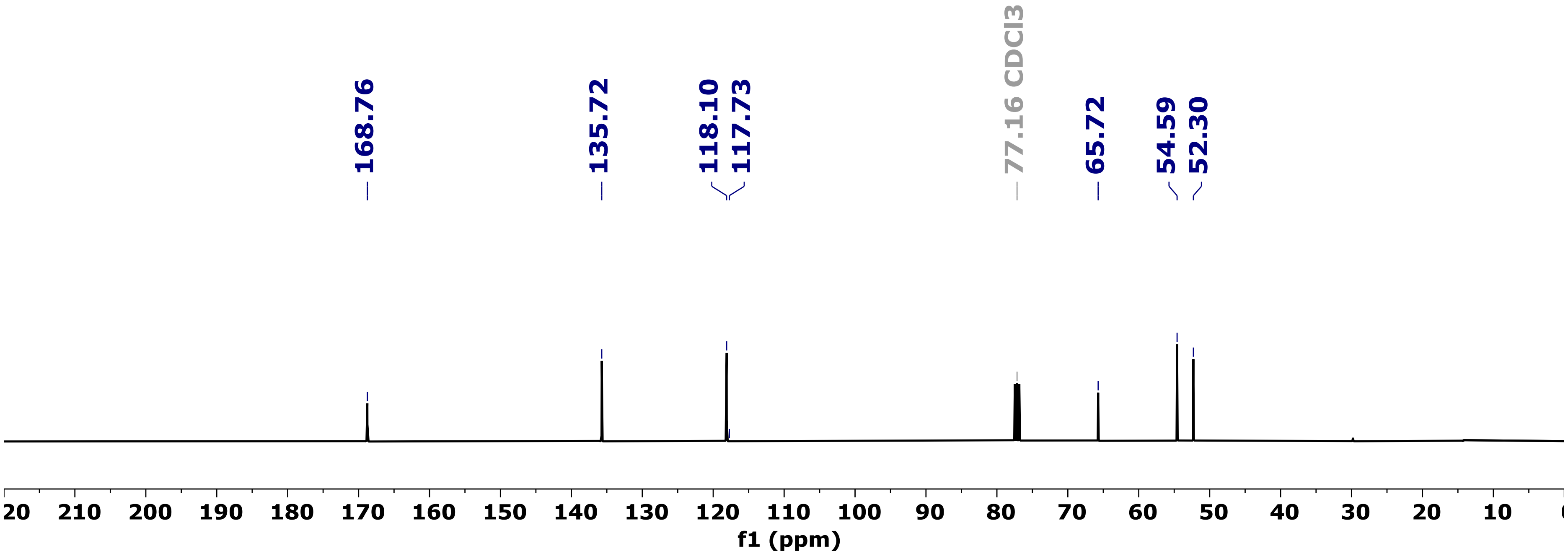

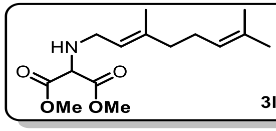

3l''

^{1}H NMR (400 MHz, $CDCl_3$)

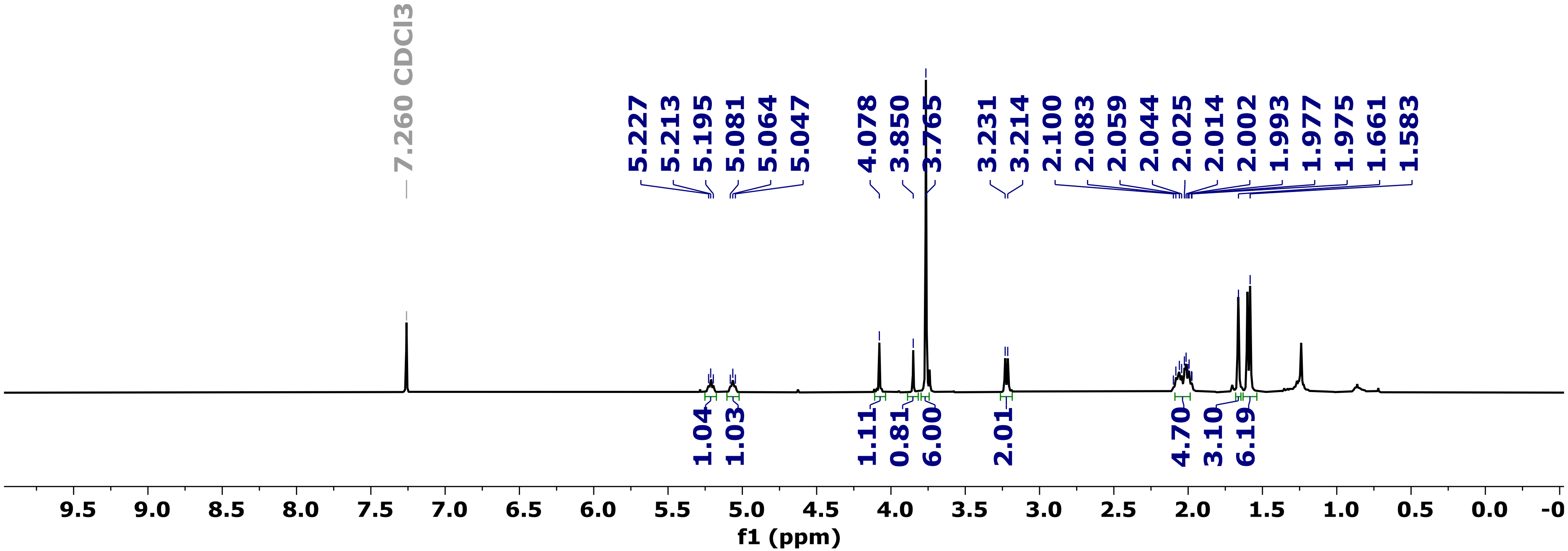

$^{13}C\{H\}$ NMR (101 MHz, $CDCl_3$)

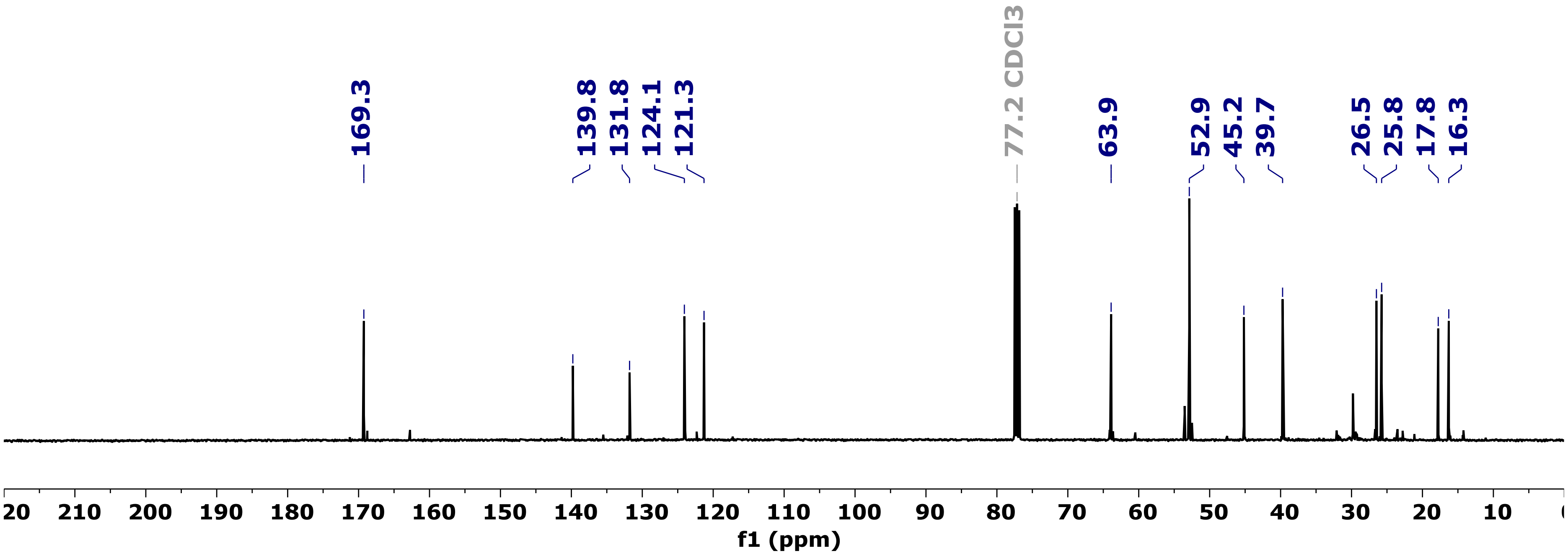

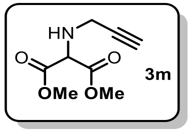

^{1}H NMR (400 MHz, $CDCl_3$)

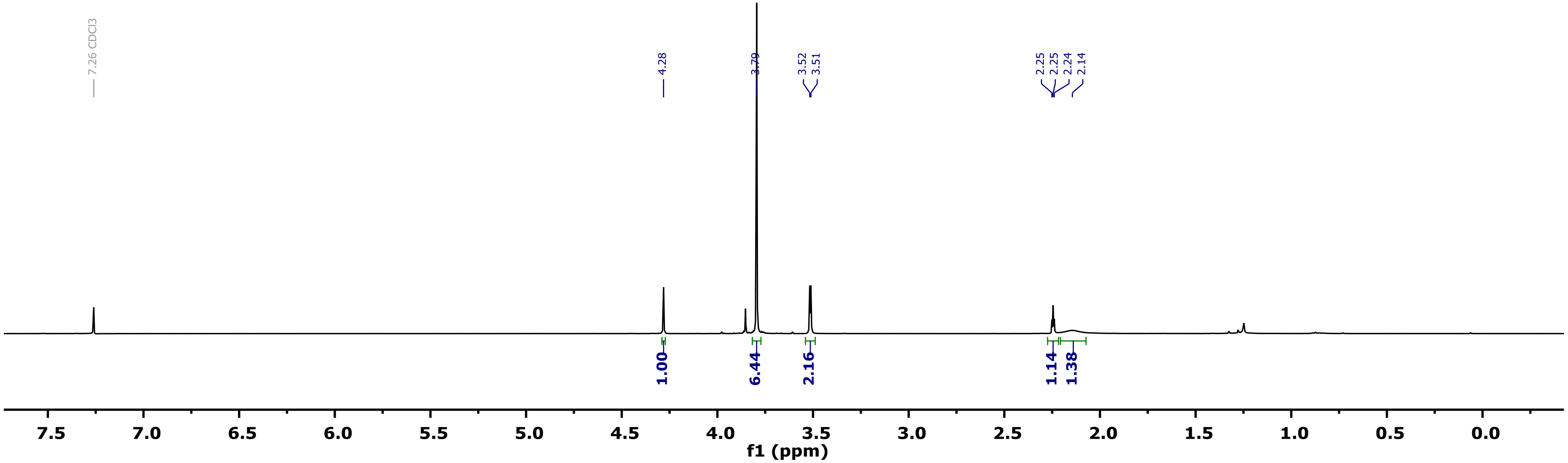

$^{13}C\{H\}$ NMR (101 MHz, $CDCl_3$)

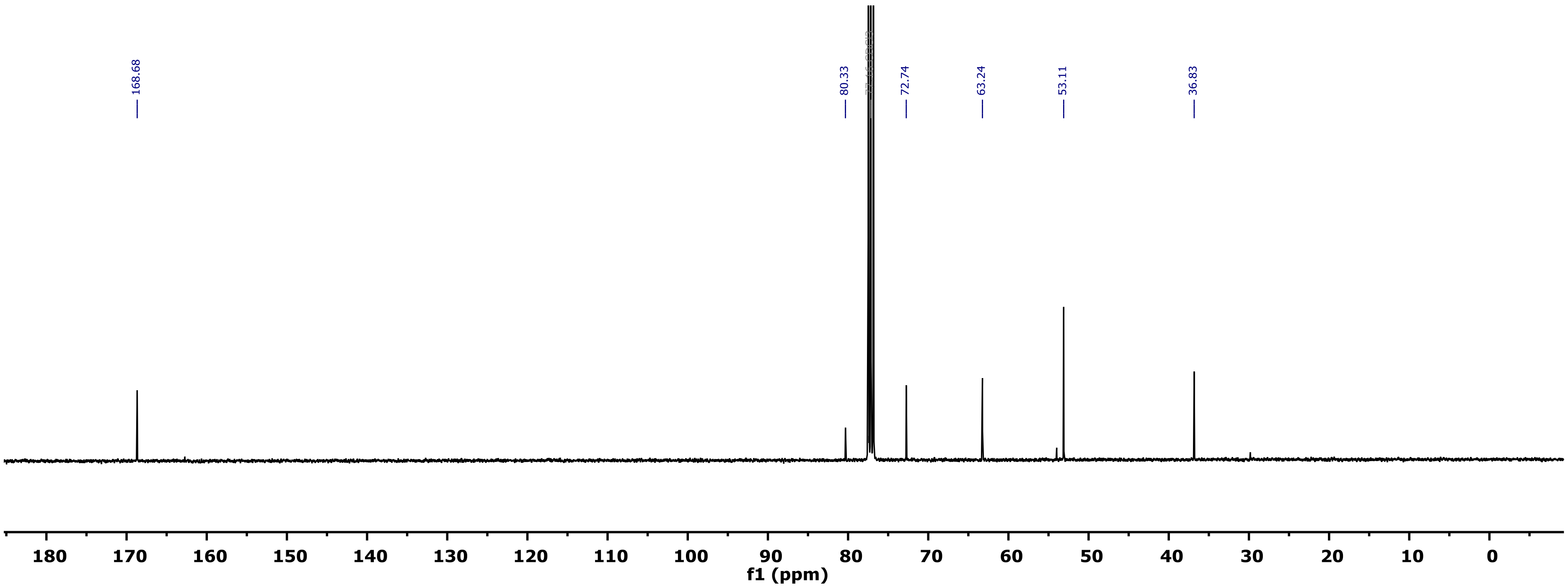

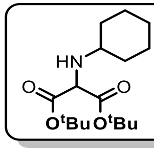

3n'

^1H NMR (400 MHz, $CDCl_3$)

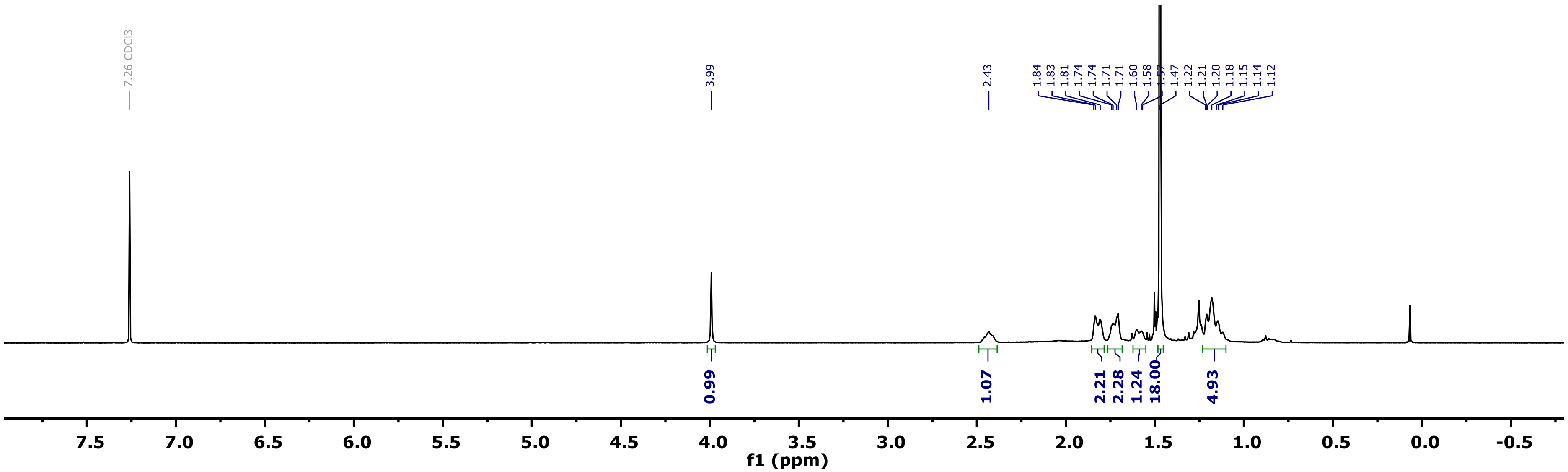

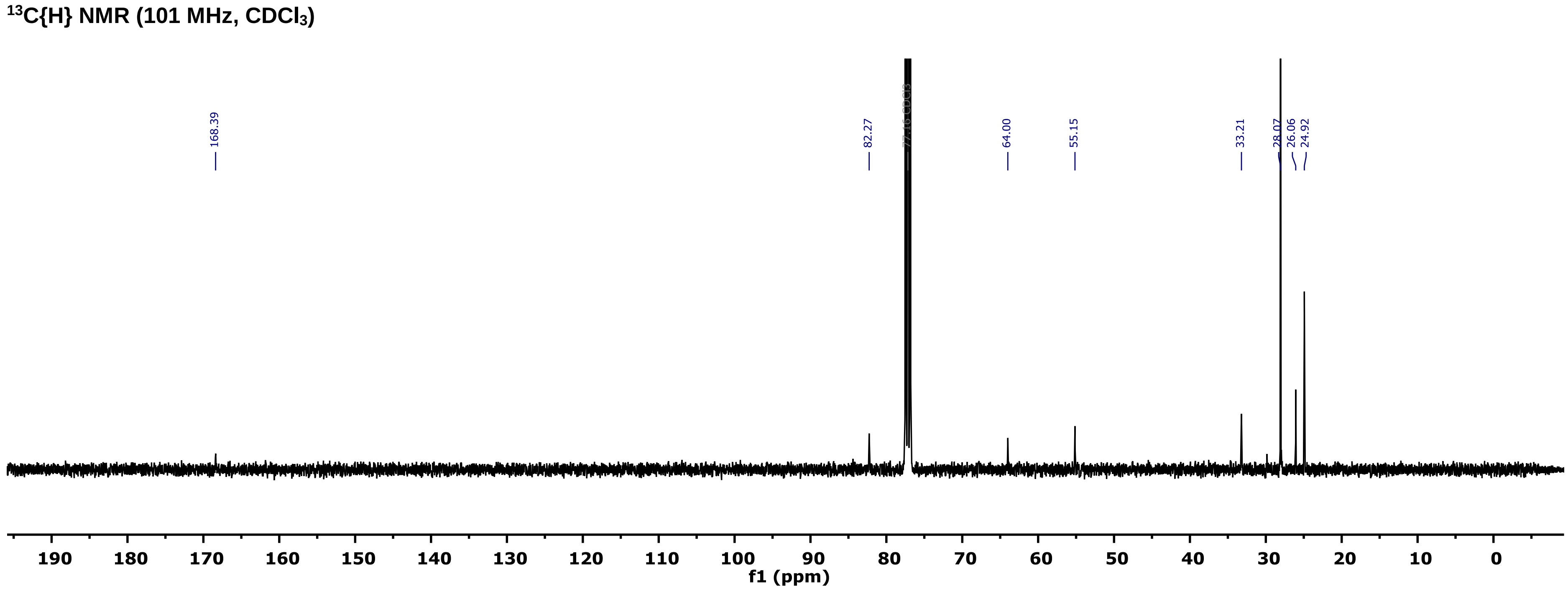

$^{13}C\{H\}$ NMR (101 MHz, $CDCl_3$)
168.39
82.27
77.16 CDCl3
64.00
55.15
33.21
28.07
26.06
24.92
190
180
170
160
150
140
130
120
110
100
90
80
70
60
50
40
30
20
10
0
f1 (ppm)

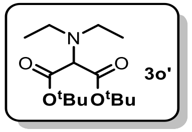

3o'

^1H NMR (400 MHz, $CDCl_3$)

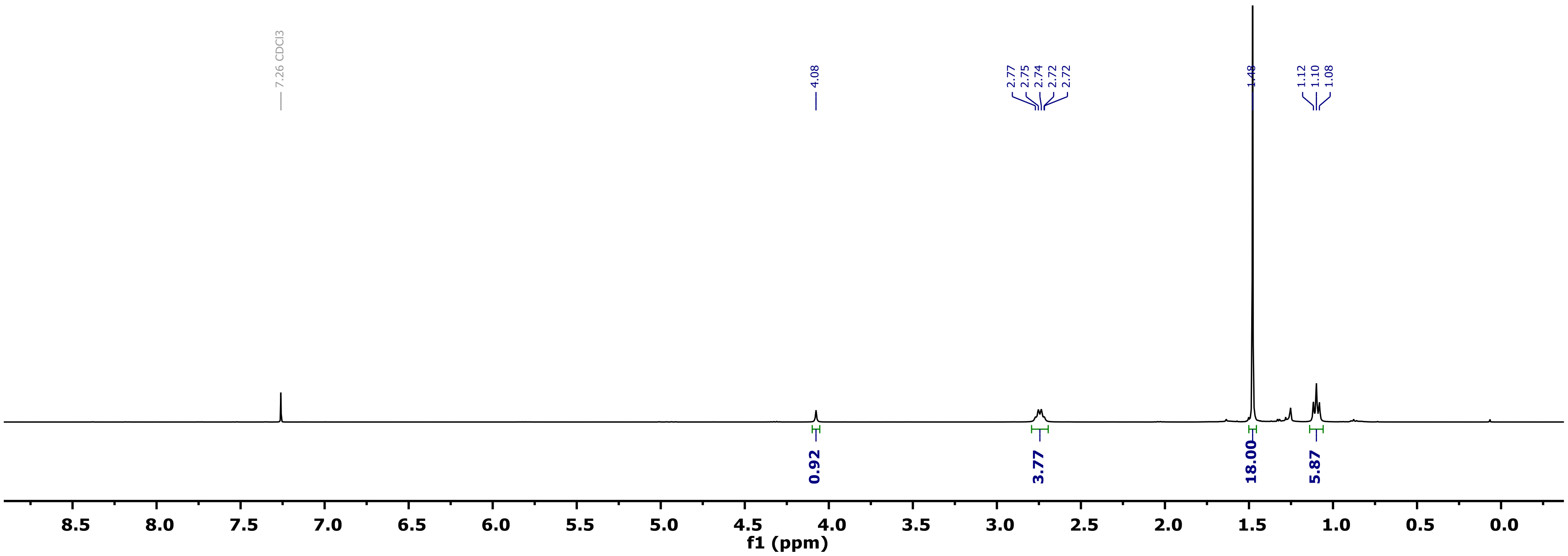

$^{13}C\{H\}$ NMR (101 MHz, $CDCl_3$)

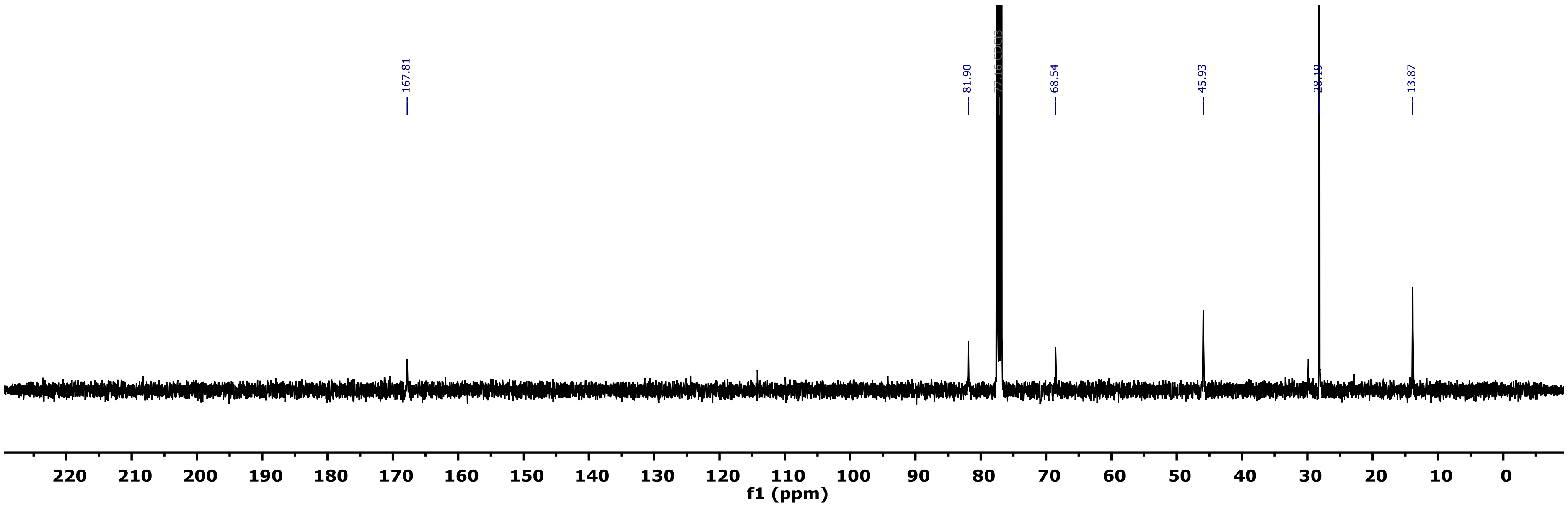

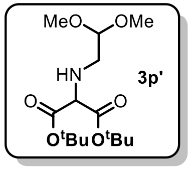

^{1}H NMR (400 MHz, $CDCl_3$)

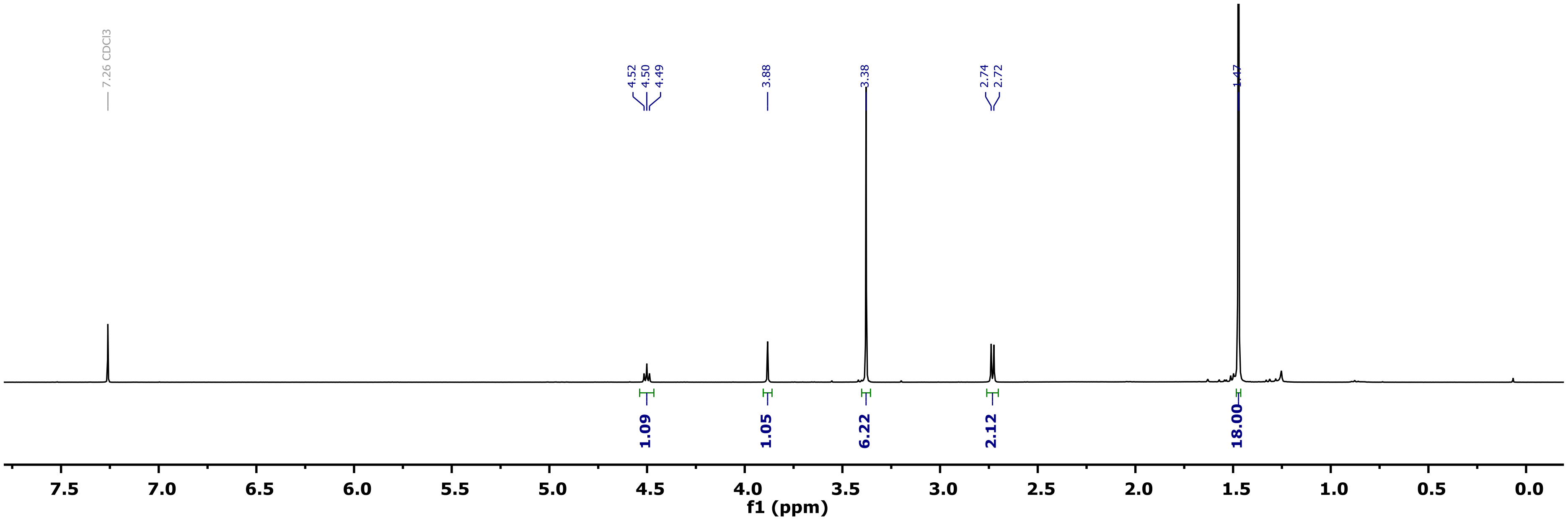

$^{13}C\{H\}$ NMR (101 MHz, $CDCl_3$)

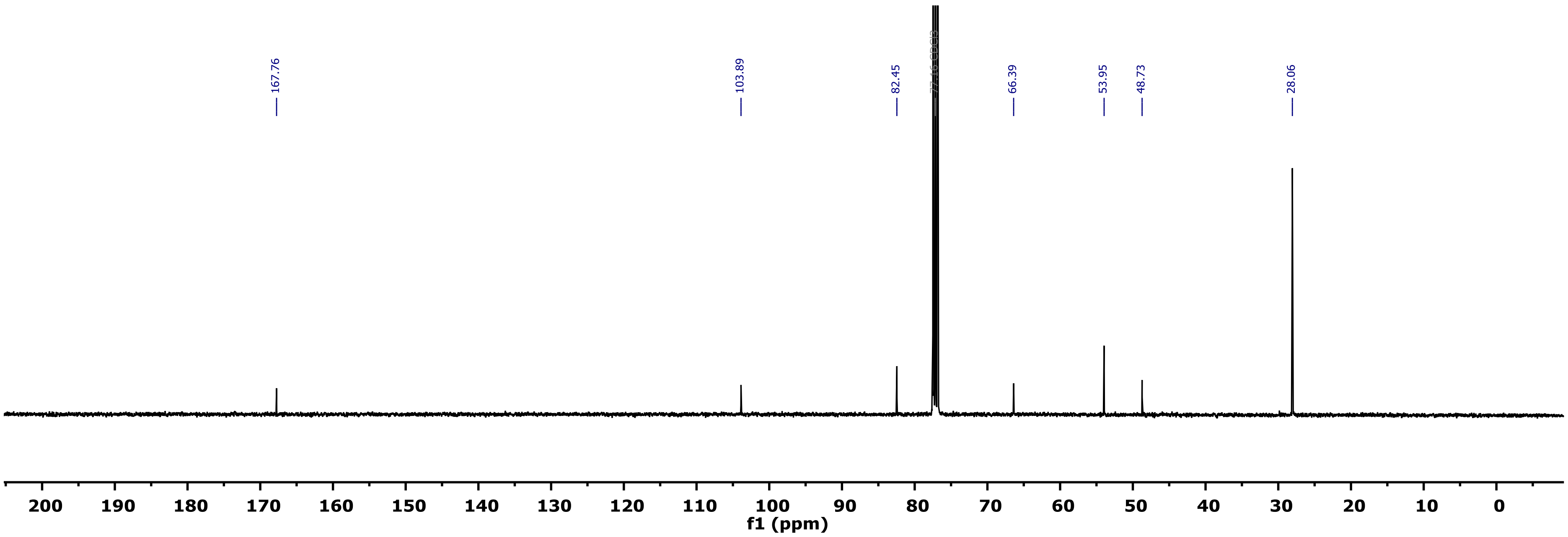

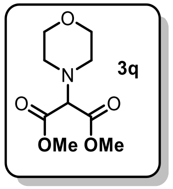

^{1}H NMR (400 MHz, $CDCl_3$)

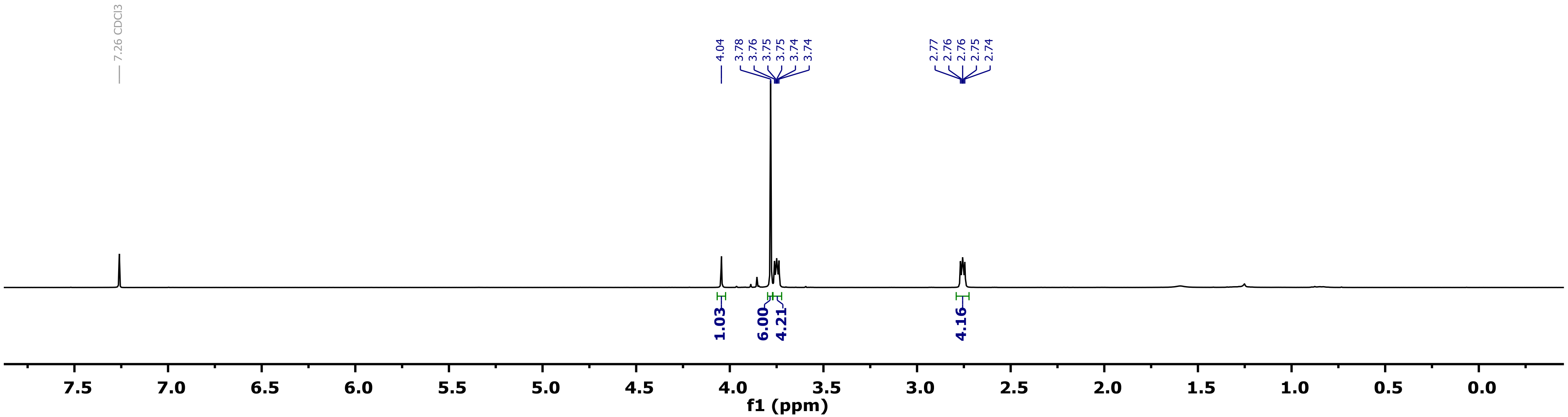

$^{13}C\{H\}$ NMR (101 MHz, $CDCl_3$)

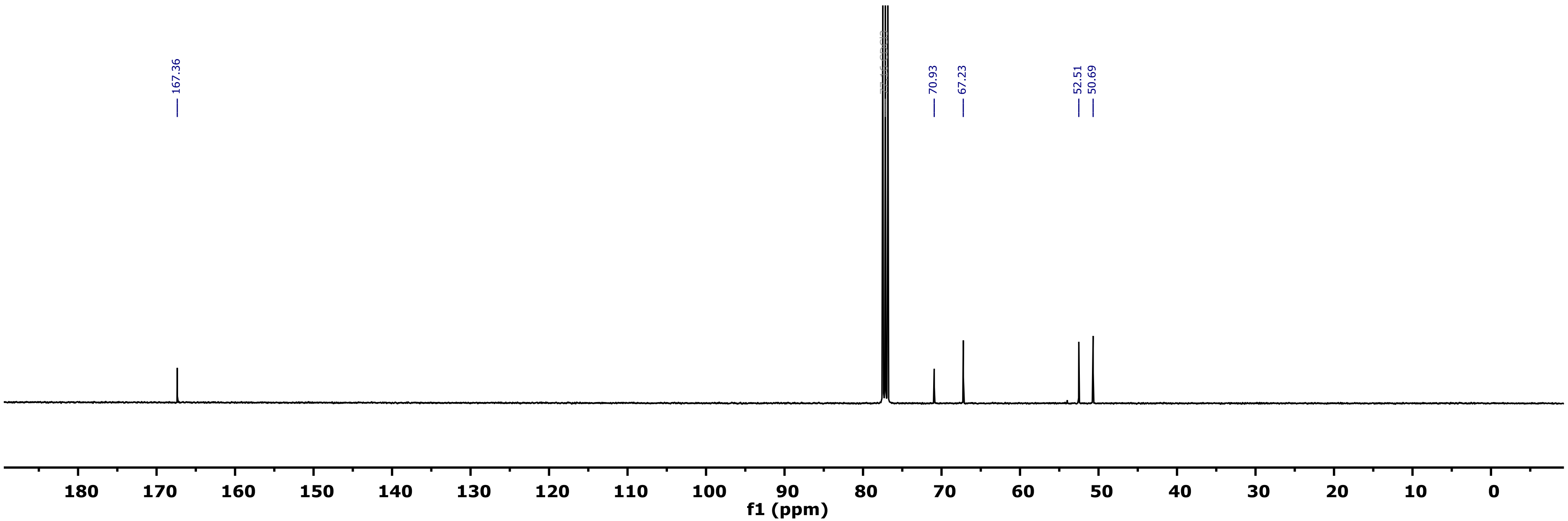

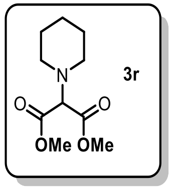

^{1}H NMR (400 MHz, $CDCl_3$)

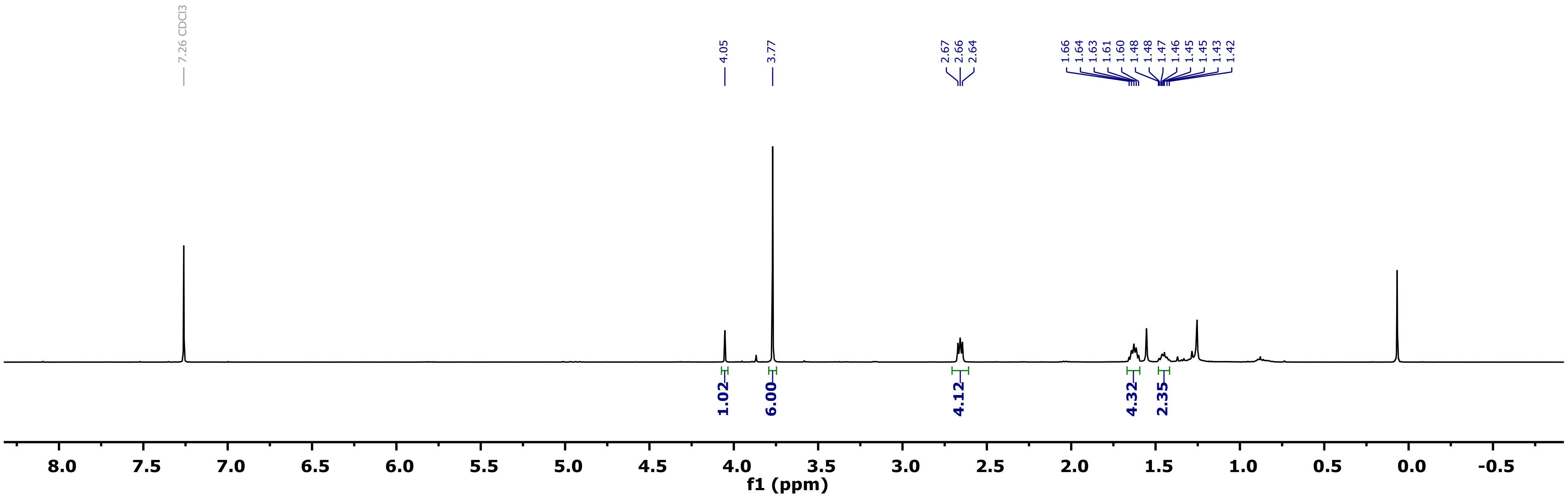

$^{13}C\{H\}$ NMR (101 MHz, $CDCl_3$)

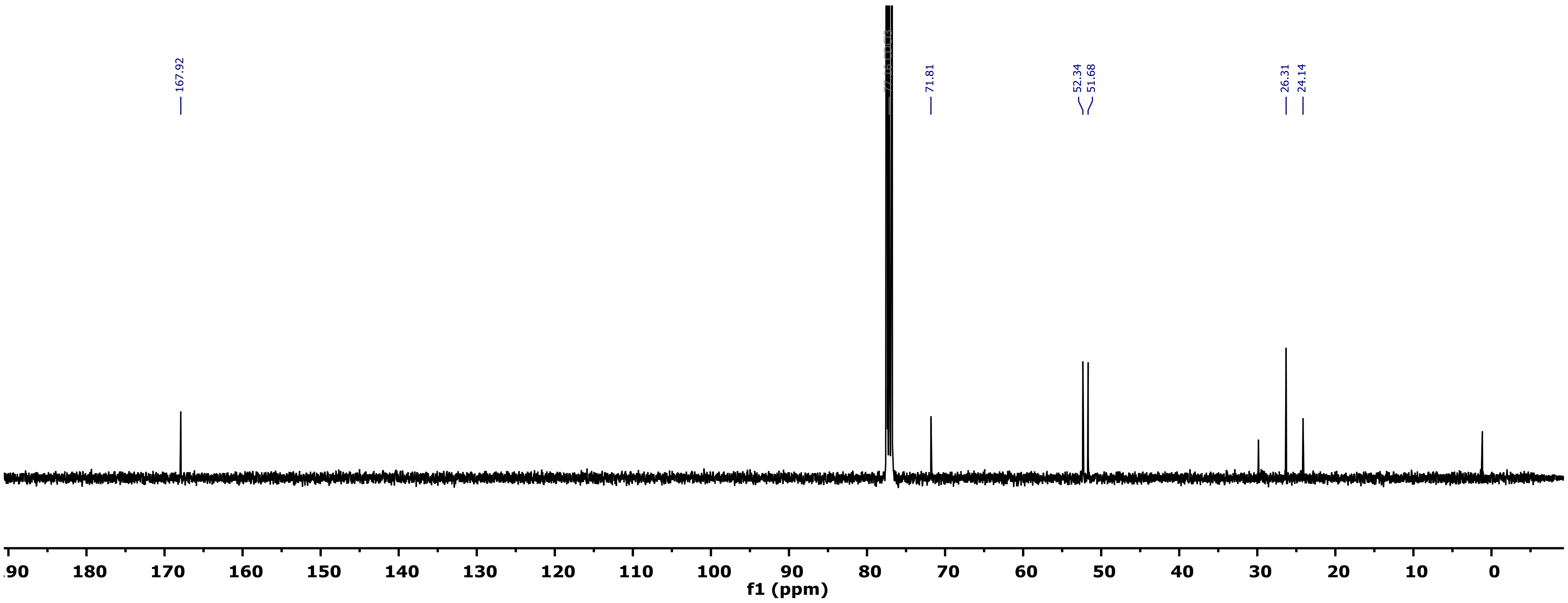

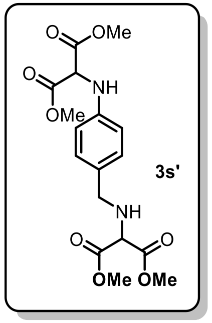

^{1}H NMR (400 MHz, $CDCl_3$)

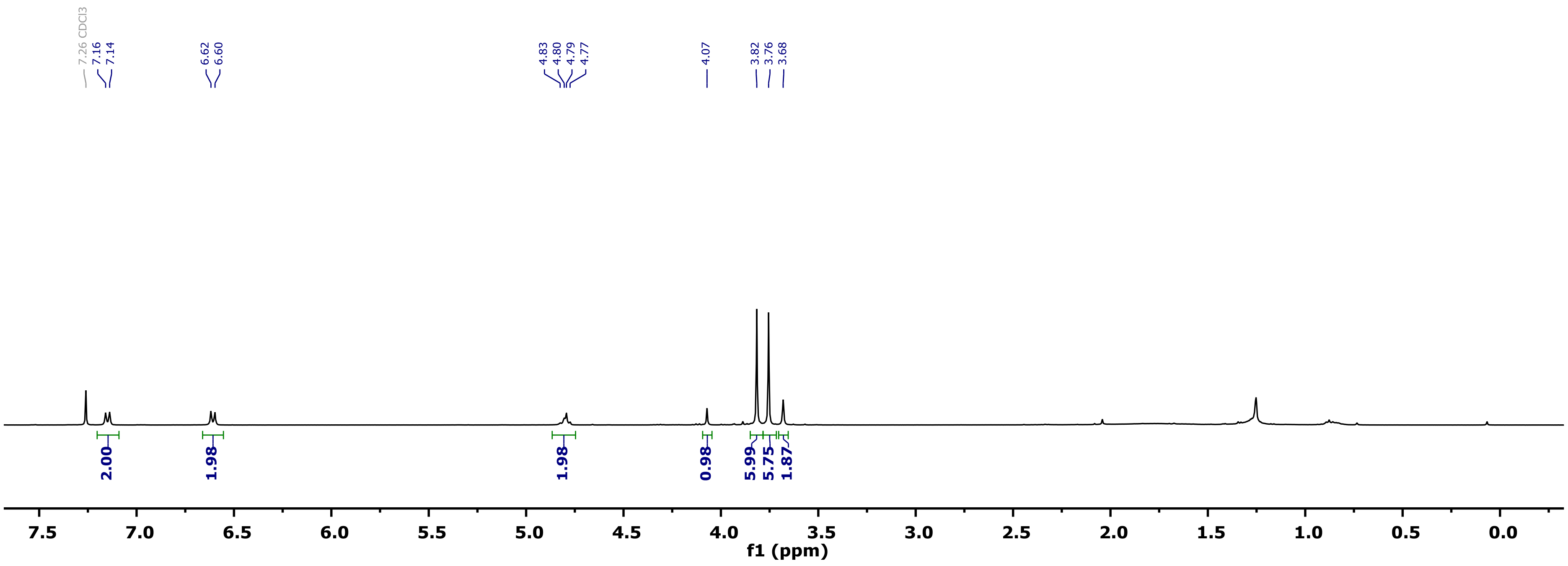

$^{13}C\{H\}$ NMR (101 MHz, $CDCl_3$)

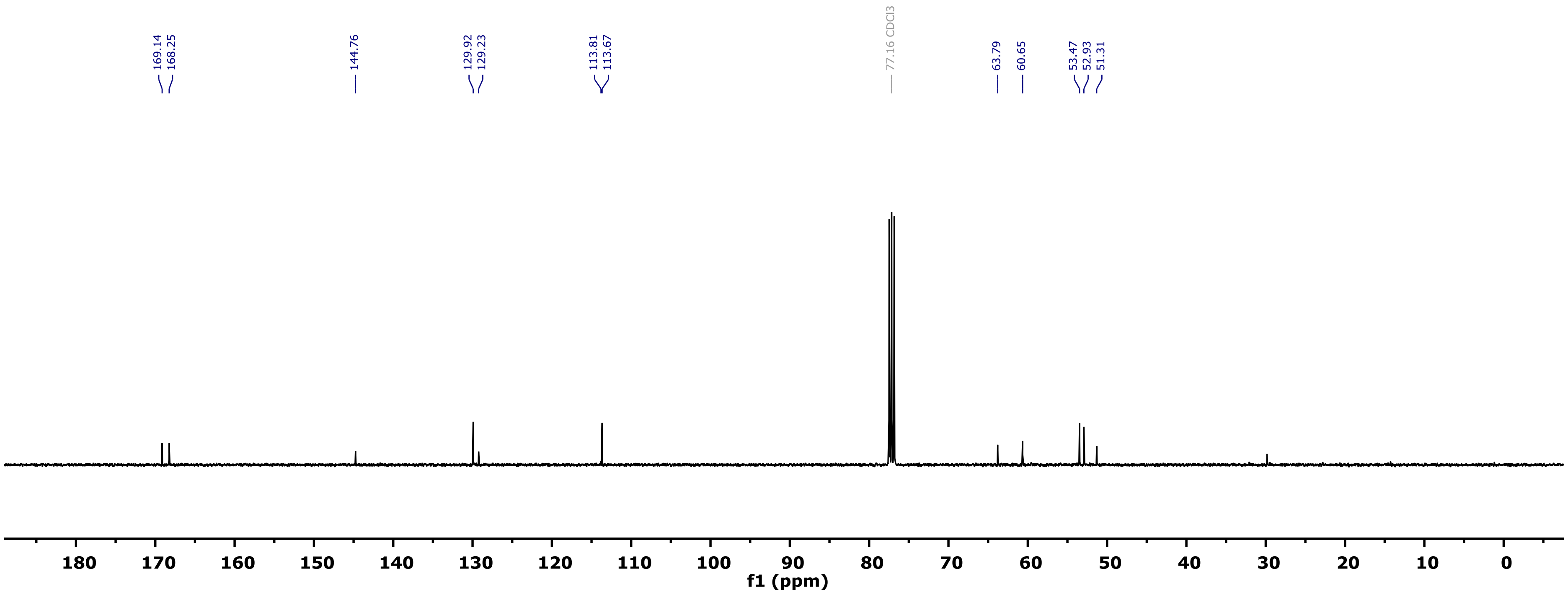

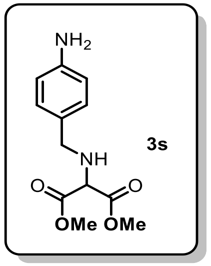

^{1}H NMR (400 MHz, $CDCl_3$)

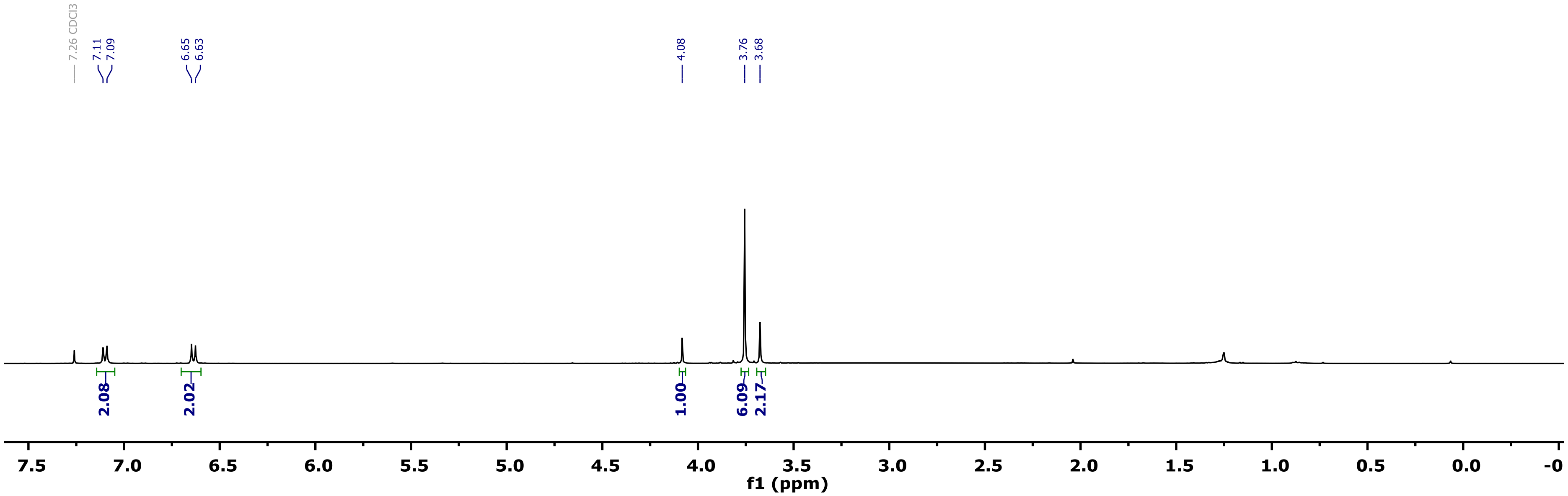

$^{13}C\{H\}$ NMR (101 MHz, $CDCl_3$)

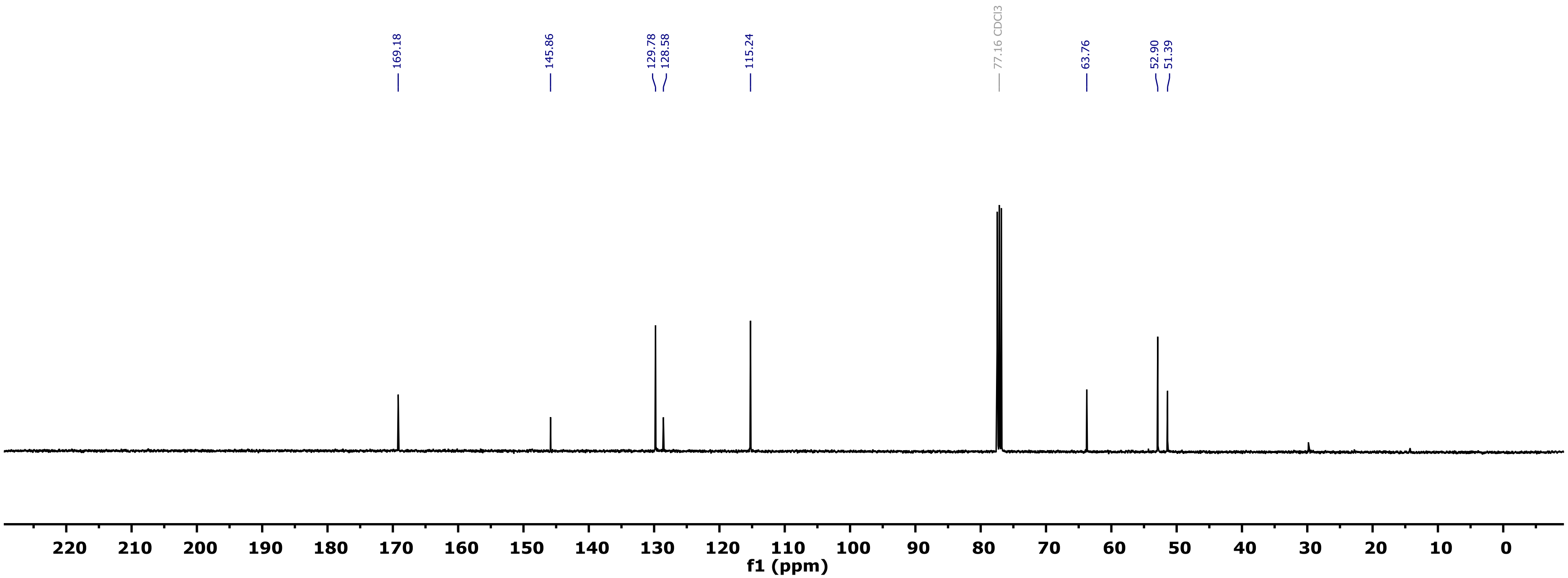

2D NMR SELECTIVE NOESY at 3.68 ppm ($CDCl_3$)

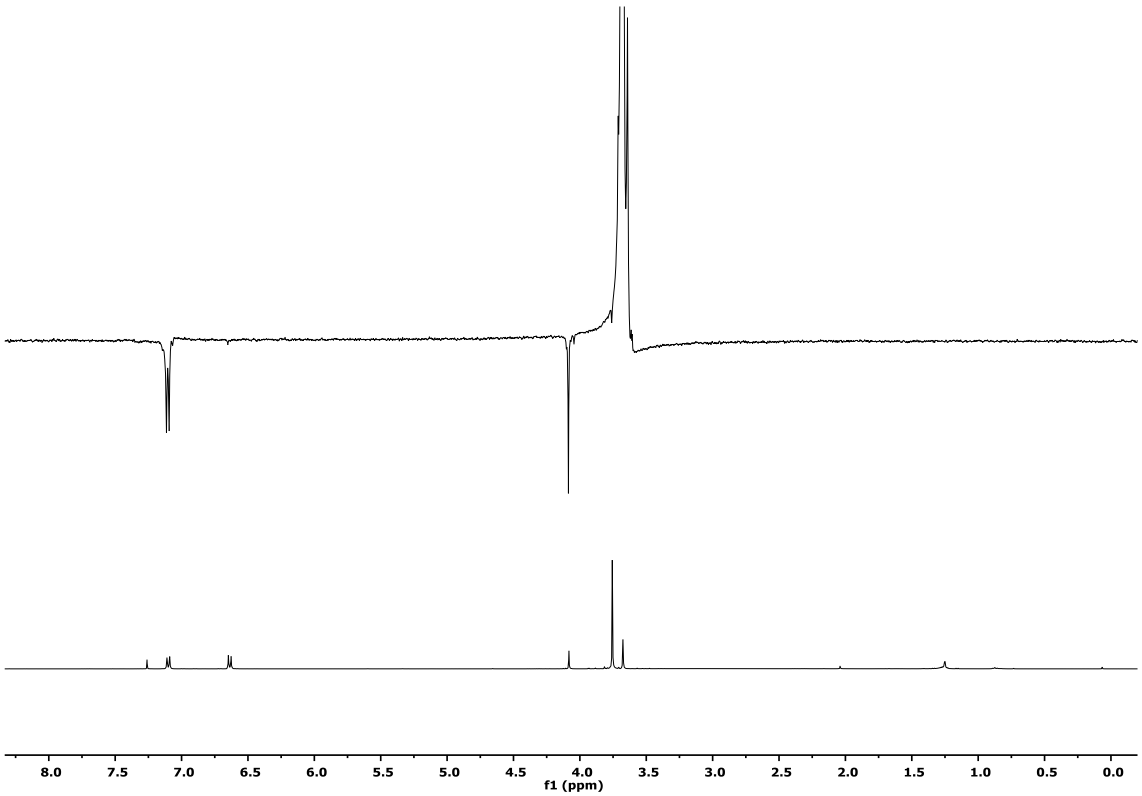

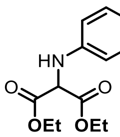

3t

^{1}H NMR (400 MHz, $CDCl_3$)

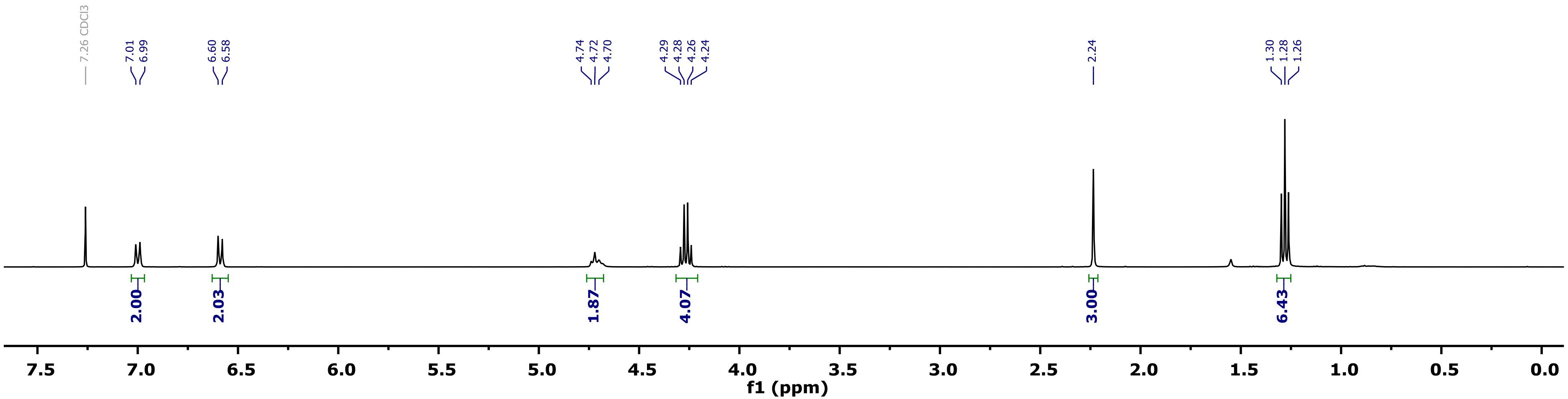

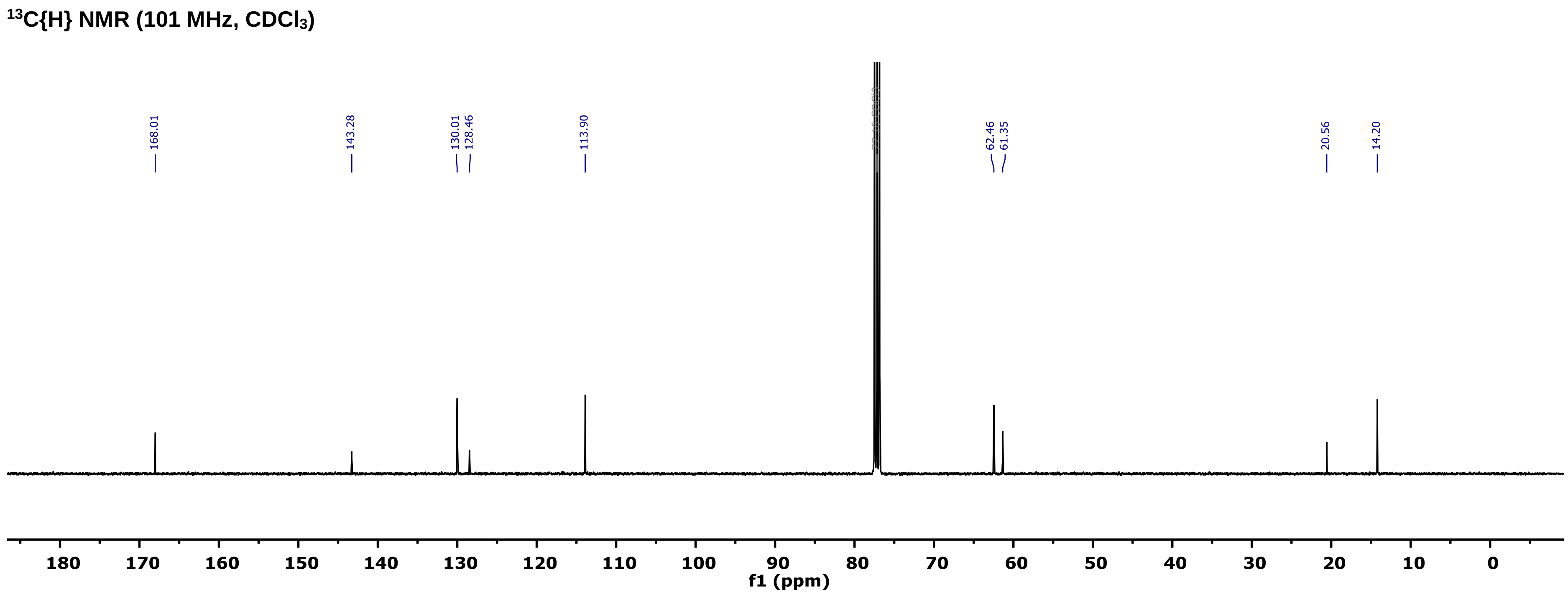
$^{13}C\{H\}$ NMR (101 MHz, $CDCl_3$)
168.01
143.28
130.01
128.46
113.90
62.46
61.35
20.56
14.20
180 170 160 150 140 130 120 110 100 90 80 70 60 50 40 30 20 10 0
f1 (ppm)

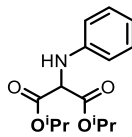

3u

^{1}H NMR (400 MHz, $CDCl_3$)

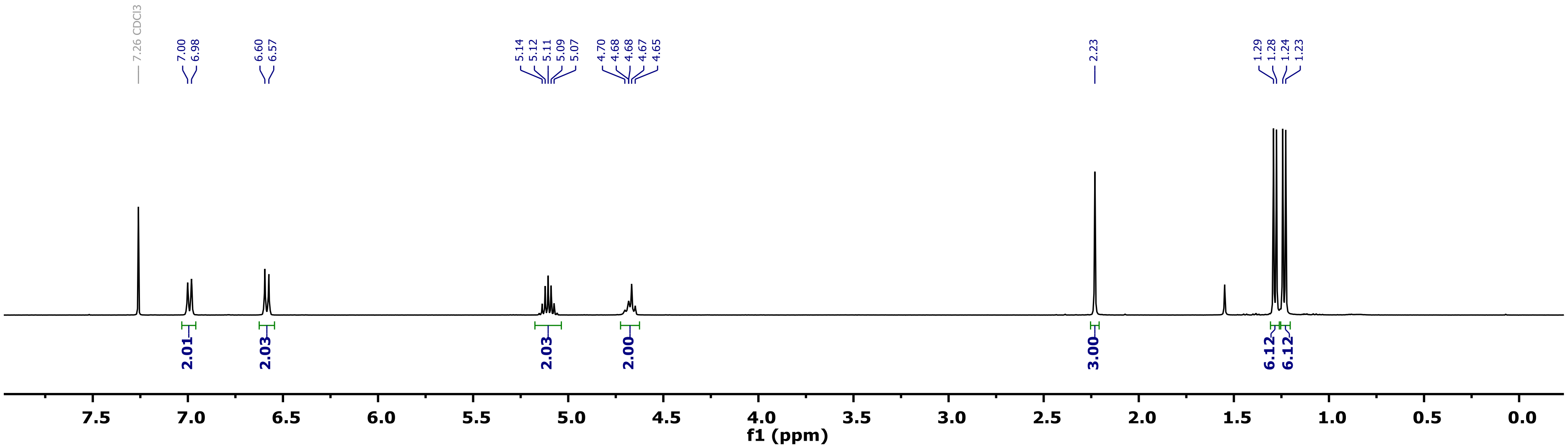

$^{13}C\{H\}$ NMR (101 MHz, $CDCl_3$)

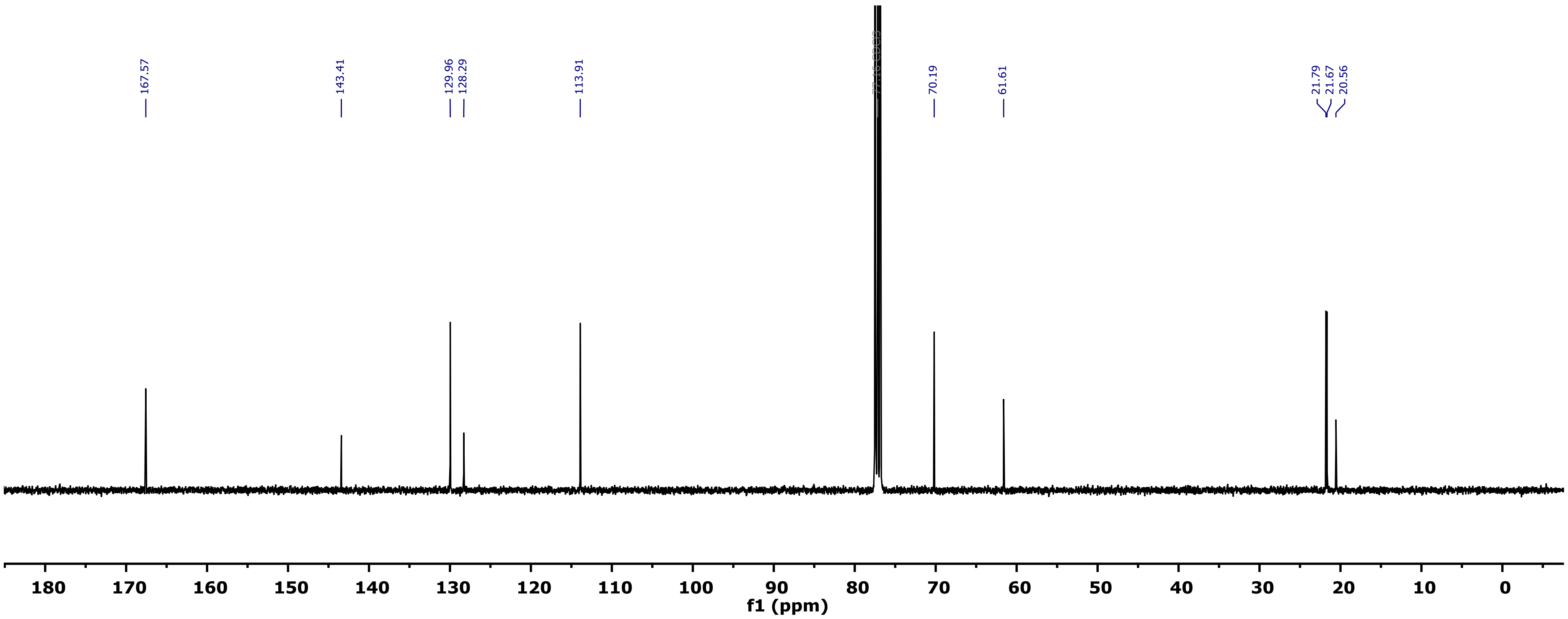

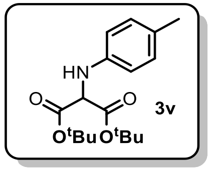

^{1}H NMR (400 MHz, $CDCl_3$)

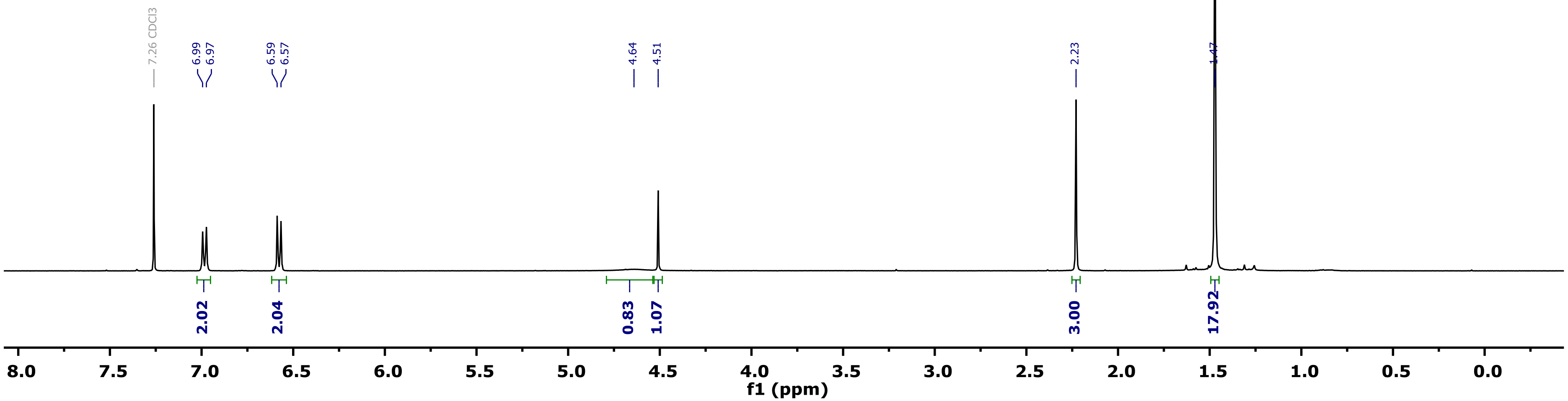

$^{13}C\{H\}$ NMR (101 MHz, $CDCl_3$)

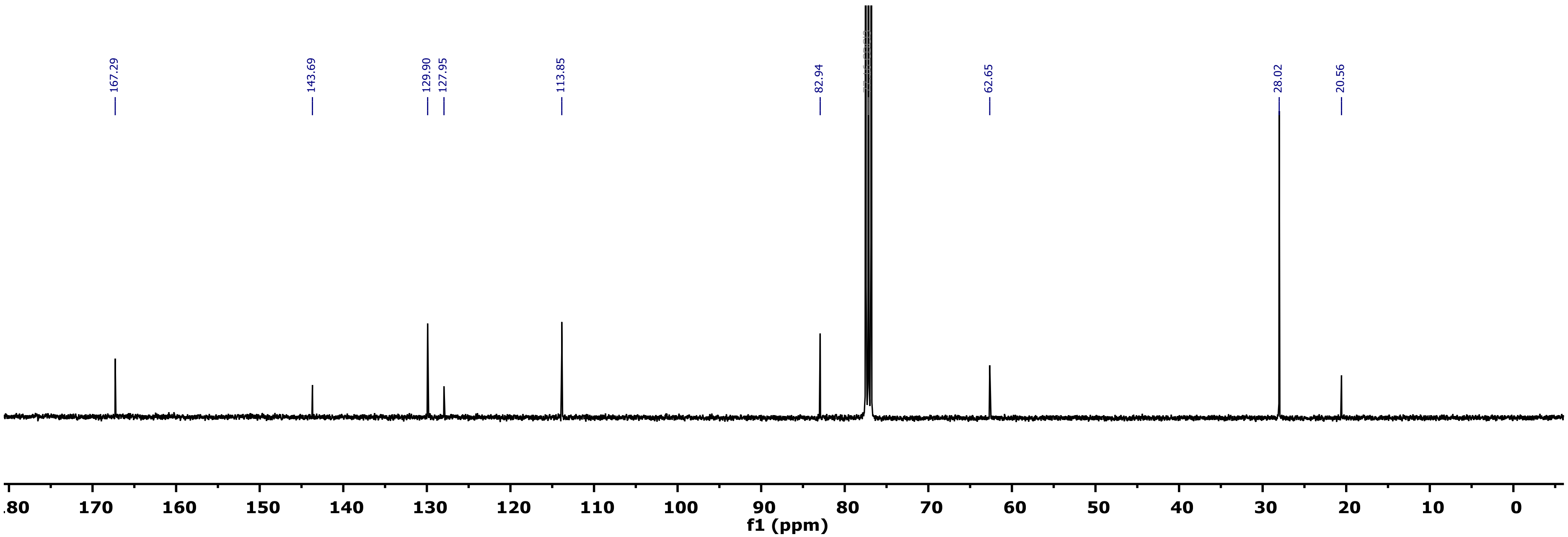

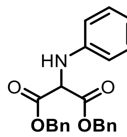

3w

^{1}H NMR (400 MHz, CDCl$_3$)

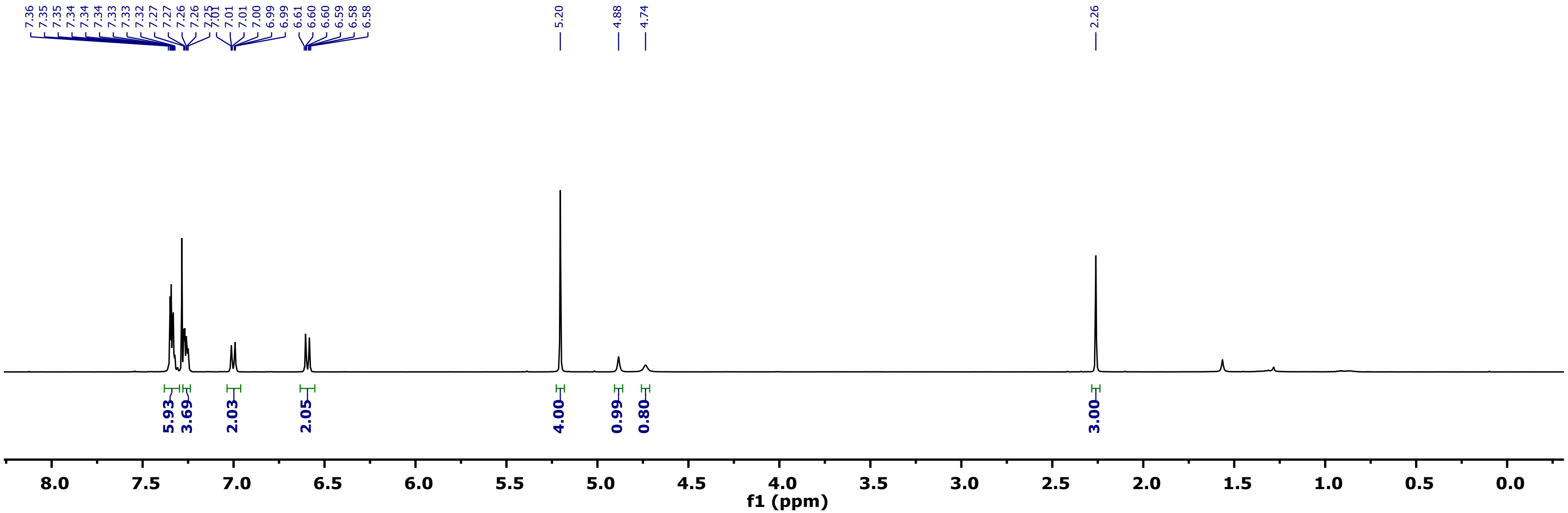

$^{13}C\{H\}$ NMR (101 MHz, $CDCl_3$)

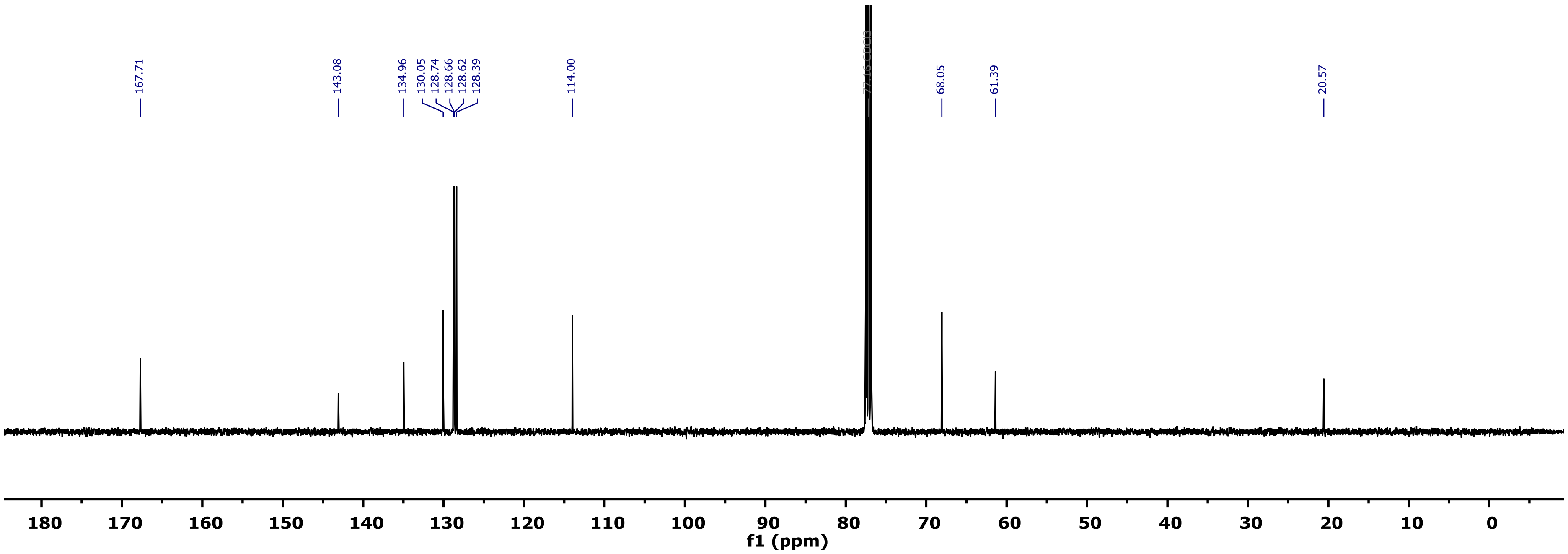

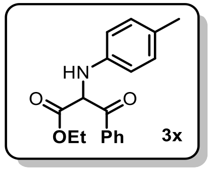

^{1}H NMR (400 MHz, $CDCl_3$)

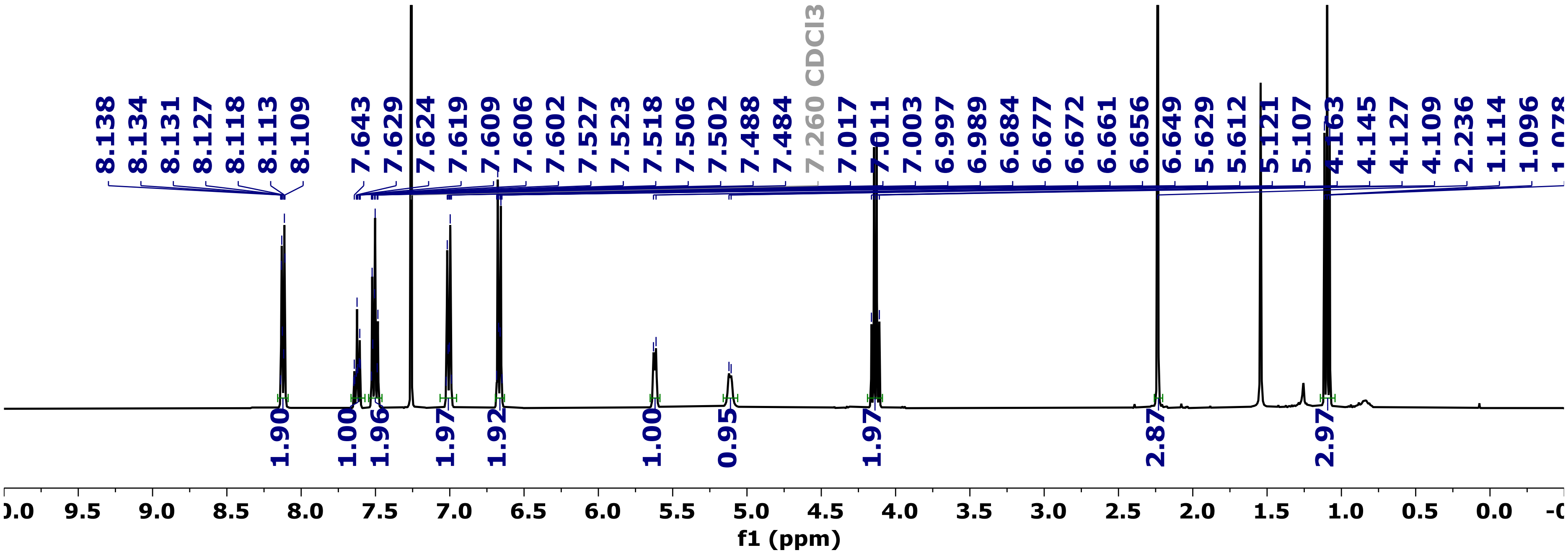

$^{13}C\{H\}$ NMR (101 MHz, $CDCl_3$)

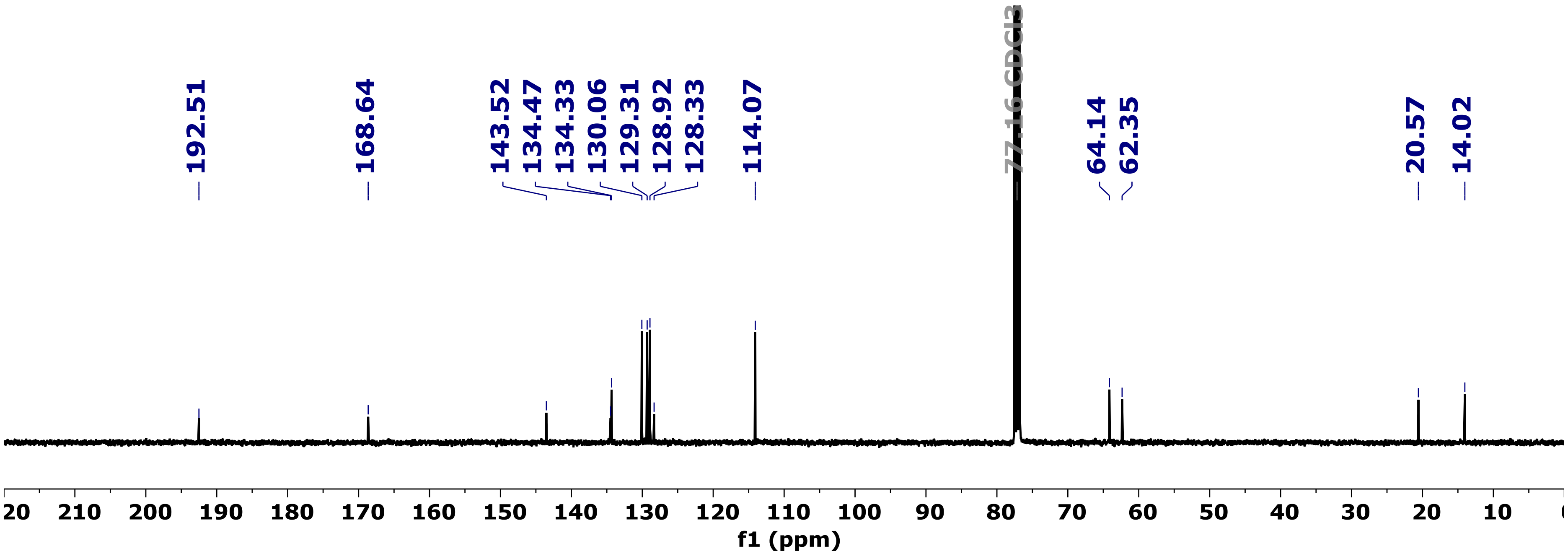

Supplement: Supplementary file 1 [file ol5c02000_si_001.pdf]
